# Identification of the miRNAome of early mesoderm progenitor cells and cardiomyocytes derived from human pluripotent stem cells

**DOI:** 10.1038/s41598-018-26156-3

**Published:** 2018-05-23

**Authors:** Ximena Garate, Alejandro La Greca, Gabriel Neiman, Carolina Blüguermann, Natalia Lucía Santín Velazque, Lucía Natalia Moro, Carlos Luzzani, Maria Elida Scassa, Gustavo Emilio Sevlever, Leonardo Romorini, Santiago Gabriel Miriuka

**Affiliations:** LIAN-CONICET, FLENI - Ruta 9 km 52.5, Belen de Escobar, Provincia de Buenos Aires Argentina

## Abstract

MicroRNAs are small non-coding RNAs involved in post-transcriptional regulation of gene expression related to many cellular functions. We performed a small-RNAseq analysis of cardiac differentiation from pluripotent stem cells. Our analyses identified some new aspects about microRNA expression in this differentiation process. First, we described a dynamic expression profile of microRNAs where some of them are clustered according to their expression level. Second, we described the extensive network of isomiRs and ADAR modifications. Third, we identified the microRNAs families and clusters involved in the establishment of cardiac lineage and define the mirRNAome based on these groups. Finally, we were able to determine a more accurate miRNAome associated with cardiomyocytes by comparing the expressed microRNAs with other mature cells. MicroRNAs exert their effect in a complex and interconnected way, making necessary a global analysis to better understand their role. Our data expands the knowledge of microRNAs and their implications in cardiomyogenesis.

## Introduction

Human embryonic development is tightly regulated in order to achieve a highly complex cell and tissue specialization. Starting from pluripotent stem cells at early stages, this regulation is able to yield unique cell functions in each organ. Among the main players in this regulated process are microRNAs, a well-characterized family of short, single stranded RNA sequences working by fine tuning the expression of key developmental genes^1,2^. Knockout animal models of the main regulatory proteins of microRNAs processing are non-viable and die *in utero* at early embryo stages^3–5^. There is an accumulated evidence about the role of microRNAs in pluripotent stem cells that expands and supports their importance in early embryogenesis^6^.

By convention, short-noncoding-RNAs are defined as those RNA molecules shorter than 200 nucleotides, and are composed of many different functional units. A fraction of them are known to regulate long-coding mRNA function, including microRNAs, short endogenous RNAs, and piRNAs. They consist on short single-stranded non-coding RNA molecules of approximately 22 bases. The mechanisms involved in microRNA genesis and gene regulation has been extensively studied^7^. Briefly, they arise from a longer structure, called the hairpin, of approximately 70 bases, which is processed in the nucleus and cytoplasm to yield the mature, functional units. MicroRNAs are transcribed to a non-mature form and sequentially processed by a set of common enzymes, including Drosha and DGCR8, both present in the nucleus as part of the microprocessor complex. The pre-microRNA produced by this complex is exported to the cytoplasm by Exportin5, where Dicer further processes them into a mature, active microRNA. These microRNAs are loaded to the RISC protein complex where they act by targeting the complementary 3′UTR region of mRNAs, either blocking their translation or inducing its degradation^8–10^. These canonical pathways have been extensively described, although now many alternative processing pathways have been recognized^11^.

Due to recent advancements in high throughput technologies and development of more sophisticated algorithms for the analysis of sequencing data, minor microRNA sequence variations have been established. They differ in internal single bases and/or in length from the canonical sequences recorded in miRbase (www.mirbase.org) and they are known as isomiRs. Several mechanisms explain isomiR biogenesis, ranging from imprecise cleavage sites of Drosha or Dicer in the microRNA maturation process, up to RNA editing by nucleotidyl transferases and exoribonucleases^12^. Variations could be present in both the microRNA 3′ or 5′-ends, although the latter are less frequent due to the possible changes in the seed sequence, and hence, in their complementary targets. However, 3′-end alterations have also been implicated in microRNA functionality, as they are thought to alter their biostability. The relevance of isomiRs is still debated but there is increasing evidence that they might have an important role in cell identity and behavior as they have specific expression patterns which may lead to a bigger impact in the mRNA regulatory network^12,13^. It has been proposed that the variations in sequence composition and length not only affect the mRNA target repertoire of the microRNAs, but also their stability and lifespan. Moreover, the isomiRs could interact with their homologue microRNAs, modulating their function and leading to a more efficient regulatory pattern^14^.

The potent ability of microRNAs in cell function regulation is given by a complex network formed by each individual microRNA. Potency is given by a double mechanism: on the one hand, a microRNA can target many mRNAs; whereas on the other hand, the 3′UTR of a mRNA can be targeted by many microRNAs. The whole mass of microRNAs then form a complex network that has been called the *miRNAome* of a cell, where there is a current set of microRNAs articulated in a given time to establish a specific cell phenotype. Small RNA-seq allows a detailed analysis of the microRNAs present at a given time. However, proper interpretation of these results require a deep analysis taking into account all the microRNAs and their variants, such as the isomiRs. With this perspective we analyzed the miRNAome of cardiac differentiation from pluripotent stem cells. We aimed to identify microRNAs networks involved in the regulation of this differentiation process. Instead of stressing the results of unique individual microRNAs, we proposed an analysis based on microRNA genome clusters and families.

## Results

### RNA-Seq results: A general miRNAome at each stage of differentiation

**Figure 1:**
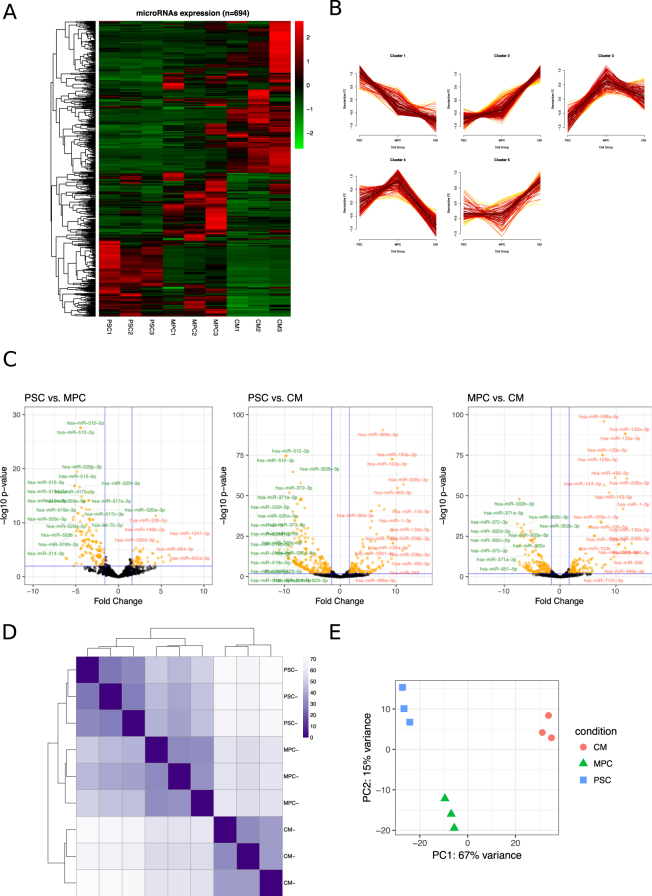
Expression of microRNAs associated with cardiac differentiation. (**A**) Heatmap shows the expression level of microRNAs in three replicates of PSC (Pluripotent Stem Cells), MPC (Mesoderm Progenitor Cells) and CM (Cardiomyocytes) microRNAs with a number of reads greater than a mean of 5 in at least one cell population. (**B**) Five distinguishable groups of microRNAs (clusters) based on a fuzzy clustering algorithm are represented. Each cluster includes a group of microRNAs that have the same dynamic expression in the three cell populations. (**C**) Pairwise analysis of microRNAs expression between cell populations is plotted in the Volcano plots (log2 FC ≥ 3 and p ≤ 0.01). Red and green microRNAs represent the most significant up and down-regulated values, respectively. (**D**) Heatmap showing differences between PSC, MPC and CM in each replicate. Higher intensity represents lower variance between the cell populations. (**E**) 2D-PCA based on the proportion of 700 microRNA expression level applied to all the replicates of the three cell populations. The PCA plot shows a highly significant discrimination of the expressed microRNAs between cell populations.

MicroRNA expression was analyzed by small RNA-sequencing in three biological replicates of three cell populations: pluripotent stem cells (PSC), primitive differentiated mesoendoderm progenitor cells (MPC^15^), and cardiomyocytes (CM) (Supplemental Fig. 1). On average, approximately 42% of the total sequences were mapped to microRNAs, while the remaining reads were related to other non-coding RNAs, including rRNA, snRNA, snoRNA, and lincRNA (Supplemental Fig. 2). We found 694 microRNAs expressed in at least one of these cell populations (arbitrarily based on the expression of at least 5 reads in each replicate, and an average of 5 or more in at least one cell group). This is a significant part of the approximately 2800 human mature microRNAs described in mirBase database (Fig. 1A; Supplemental File 1 (all microRNAs) and 2 (expressed microRNAs)). The most highly expressed microRNAs in PSC and CM are shown in Tables 1 and 2 respectively. The microRNA expression pattern significantly varied across cell populations. Although intra-group expression levels certainly showed some variations from sample to sample, a consistent expression pattern can be observed in the heatmap where some microRNAs increased or decreased throughout differentiation. We sought to further characterize this dynamic behavior during the three stages of differentiation by performing a fuzzy hierarchical analysis^16,17^. Fuzzy plots obtained by this method can be seen in Fig. 1B. After optimizing cluster adjusting (Supplemental Fig. 3) we found five different expression patterns of microRNAs. First, some microRNAs increased early once the differentiation had initiated and continued increasing up to the later cardiac stage. Second, some microRNAs increased at the later stage. Third, some other microRNAs decreased at the end of differentiation. Fourth, some rapidly decreased once differentiation was initiated. Finally, a group of microRNAs increased during the MPC stage, but decreased at the cardiac stage. MicroRNAs represented in each of these clusters can be found in the Supplemental File 3.

**Table 1:** Most highly expressed microRNA in PSC compared to CM.

microRNA	PSC1	PSC2	PSC3	CM1	CM2	CM3	log2FC
hsa-miR-302b-3p	314847	376199	427597	1969	1901	1363	−8.55
hsa-miR-302a-5p	163784	213498	141767	3357	2231	835	−6.98
hsa-miR-372-3p	109988	17394	43904	126	209	224	−9.15
hsa-miR-302d-3p	22718	33794	18820	376	353	223	−7.08
hsa-miR-302a-3p	17769	15461	20310	211	166	131	−7.70
hsa-miR-302c-3p	17042	15771	16136	196	118	102	−7.70
hsa-miR-92a-3p	10865	16838	8793	5889	4798	4973	−1.96
hsa-miR-92a-3p	10523	16060	8442	5446	4487	4575	−1.96
hsa-miR-20a-5p	9663	9099	10467	1153	1791	2088	−3.44
hsa-miR-363-3p	10751	8047	9329	1227	674	1023	−4.09

**Table 2:** Most highly expressed microRNA in CM compared to PSC.

microRNA	PSC1	PSC2	PSC3	CM1	CM2	CM3	log2FC
hsa-miR-143-3p	966	524	457	158524	134668	113107	6.93
hsa-miR-100-5p	655	276	584	87971	98908	37292	6.47
hsa-miR-30d-5p	4215	3747	3322	26154	30870	30846	2.13
hsa-miR-99a-5p	43	69	30	20866	20682	10214	7.84
hsa-miR-22-3p	2084	3294	1339	8564	13724	22444	1.83
hsa-miR-125a-5p	603	1108	831	6504	13054	22746	3.08
hsa-miR-27b-3p	1191	861	906	12334	9900	10577	2.67
hsa-miR-125b-5p	125	168	91	5410	12738	13794	5.51
hsa-miR-125b-5p	121	162	88	5282	12550	13631	5.51
hsa-miR-499a-5p	25	29	27	7328	10437	11278	7.62

We performed a differential expression analysis between each cell population. As expected, many microRNAs were differentially expressed in each cell group. Volcano plots were generated by comparing the three cell populations in pairwise (Fig. 1C; Supplemental Files 4–6). CM population showed the largest microRNA expression difference when comparing both PSC and MPC. There was a greater overlapping in the microRNA repertoire between PSC and MPC as they are closer in the developmental time course. For example, mir-302 family was highly expressed in PSC but maintained an upregulated expression at the mesoderm stage, with a significant decrease thereafter. These differences among groups were also seen in a distance heatmap (Fig. 1D). In agreement with the volcano plots, differences were more pronounced at the later stage of differentiation (cardiac stage) than between PSC and MPC. Finally, a principal component analysis (PCA) showed a marked independence of each cell group (Fig. 1E), stressing the significant differences among cell populations and validating the replicates.

### isomiR analysis

**Figure 2:**
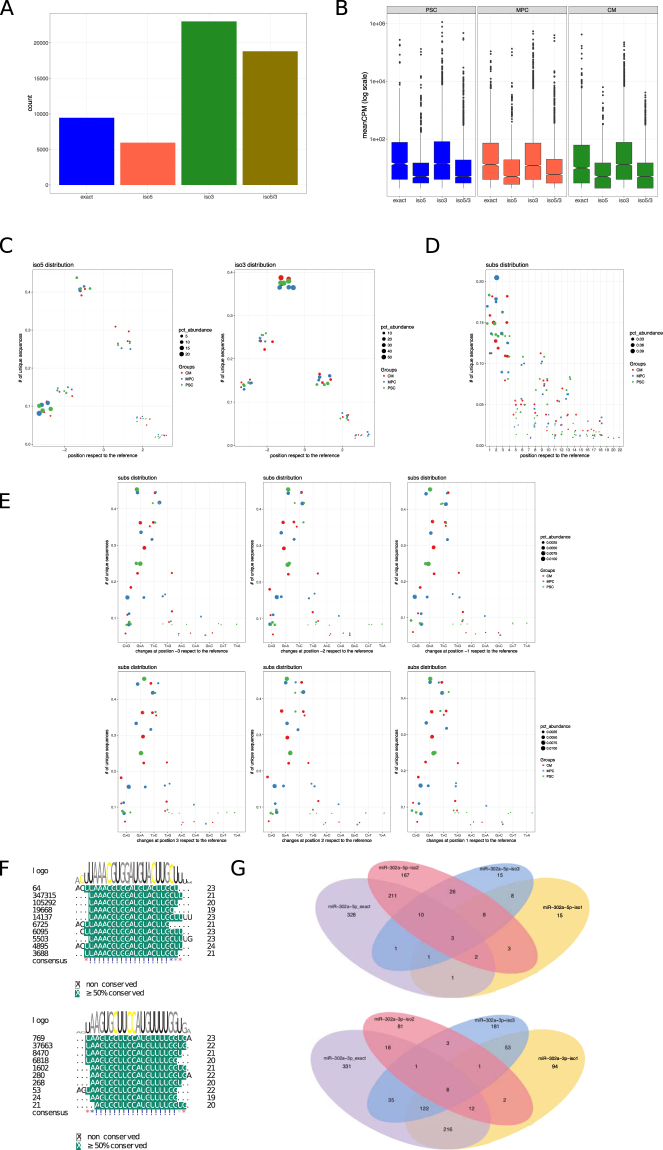
Heterogeneity and abundance of isomiRs associated with cardiac differentiation. (**A**) Bar graph showing the number of isoforms found by miraligner. Of note, there is a significant abundance of isomiRs in the samples, and particularly with variations in both extremes. (**B**) Expression levels of each isoforms. Even though 5-end isomirs are expressed at a lower level, some of them are highly expressed in the three cell groups. (**C**) Dot plots show the distribution of the isomiRs according to their abundance and the position of sequence modifications. Left dot plot corresponds to 5′-end isomiRs and right dot plot to 3′-end isomiRs. (**D**) IsomiRs with sequence substitutions are shown in the dot plot according to the abundance and the position of the substitution. Cell populations are discriminated by different colors. (**E**) Multiple alignment between mir-302a-3p/5p and their isomiRs are shown. Read counts of each isomiR is presented at the left of the sequence and sequences with a common seed are grouped together and named as iso-1, iso-2 and iso-3 for each miR302a. (**G**) Targets were predicted for each isomiR and microRNA and the overlapping between them is shown in the Venn Diagram graphs. For the analysis we considered targets with a score above 0.8.

miRDeep2 software does not report variants from the published canonical microRNA sequences of mirBase, although it provides a pdf file for each microRNA detailing the aligned sequences (see Supplemental File 7 as an example). Clearly, many reads present a sequence that is slightly different from the exact microRNA. However, any sequence with more than one mismatch or a sequence with a larger extension than two bases in the 5′-end and more than five bases in the 3′-end are discharged from the analysis, and all the sequences that fulfill with the parameters are recorded in the alignment phase as part of the same microRNA molecule. There is accumulating evidence that suggest that these minor modifications in the 5′- and 3′-arms may be functional, and more importantly, their gene targets may or may not overlap with the canonical microRNA targets, hence it has been suggested that a proper analysis of the miRNAome should include the study of isomiRs^18^. In the last years several packages have been developed for the analysis of isomiRs. We used the miraligner/isomiR package to detect these isoforms in our samples as it has been shown to be highly accurate^19,20^, although we also ran the analysis using Chimira and Isomir-SEA^21,22^.

miraligner/isomiRs packages searches for matchings of the exact microRNA sequences and distinguished those with modifications in the 5′-end and in the 3′-end (up to 3 extensions or deletions in the extremes). Figure 2A shows the non-filtered count of exact sequences and those with modifications in either one or both ends. Overall, 6,359 sequences were detected (Supplemental File 8). After filtering by a mean of 5 or more in at least one cell group (as in the previous analysis), there were 2809 sequences. Modifications were frequent, and even the exact sequence was in fact less numerous than the modified one. This finding was consistent when the analysis was run with other softwares, with even more modifications beyond position 3 (Supplemental Fig. 4).

We then examined the expression levels of the isomiRs in comparison to exact microRNAs. Figure 2B shows that many isomiRs were highly expressed, even to the level of the exact microRNAs. There were no differences between the expression levels among cell groups (p = 0.18 by ANOVA). Figure 2C shows the position of the isomiRs modification along the microRNA sequences. As it can be seen, there were no differences among cell groups. When considering modifications at the 5p-end, most changes occurred at position -3 (that is, deletion of 3 bases). In the case of the 3p-end, most modifications occurred at position -1. In any case, modifications were frequent at all positions. Nucleotide substitutions (“SNPs”) occurred at a low percentage. However, it is interesting to note that substitutions were more frequent at the 5p-end extreme (precisely at the seed) (Fig. 2D). Moreover, these nucleotide substitutions involved a particular subset of them, including C > G, G > A and T > C (Fig. 2E). Both findings did not occur at random, and hence, we believe that they correspond to real base substitution and not sequencing errors.

The highly expressed isomiRs could have an impact on the regulation of gene expression producing an important transcriptomic effect, particularly those with variations in the 5′-end. As an example, we described an interesting (and extreme) case of a seed-modificated isomiR. mir-302 family members are highly expressed microRNAs in PSC. We noticed a higher expression of several isomiRs related to hsa-mir-302a, particularly at the 5′-end. Figure 2F shows the alignment of isomiRs and exact sequences, as well as the expression level. As can be noted, the seed is significantly changed in these isomiRs. The potential targets of these sequences were analyzed (Fig. 2G) by prediction using the DIANA tools (MR-microT software^23^,). Interestingly, for the miR-302a-3p there were several common targets between one of the isomiRs and the microRNA exact sequence, but many were exclusive for each isoform (94 for 302a3p-iso1, 81 for 302a3p-iso2 and 181 for 302a3p-iso3). Similarly, isoforms of the miR-302a-5p had several unique targets (242) that were not present in the repertoire of targets regulated by the exact microRNA. To explore whether the isomiRs regulate genes from different biological processes, we analyzed the gene ontology terms (GO) associated with targets specific to the exact microRNA and targets common to both microRNA and isomiRs. Surprisingly, the analysis of the GO terms including all predicted targets (from both microRNA and isomiRs), revealed an enrichment in new biological processes not found by the analysis of the exact microRNA targets alone (p < 0.01), such as the Wnt signaling pathway, calcium modulating pathway, or regulation of cellular component size (Supplemental Fig. 5). Hence, isomiRs potentially expand and strengthen the regulatory network of the microRNAs. Finally, we evaluated the correlation between the target affinity scores provided by MR-microT of the exact microRNA and isomiRs. A linearity in this regression means that there is a correlation between the probability of a gene to be regulated by both microRNA and respective isomiR. Interestingly, we observed that none of the analyzed pairs showed a high correlation, implying that even though the microRNA and isomiRs might have some overlap in the regulated genes they present different target affinities, hence denoting that they potentially exert their inhibitory effects in an additive and complementary manner more than in a redundant way (Supplemental Fig. 6).

**Figure 3:**
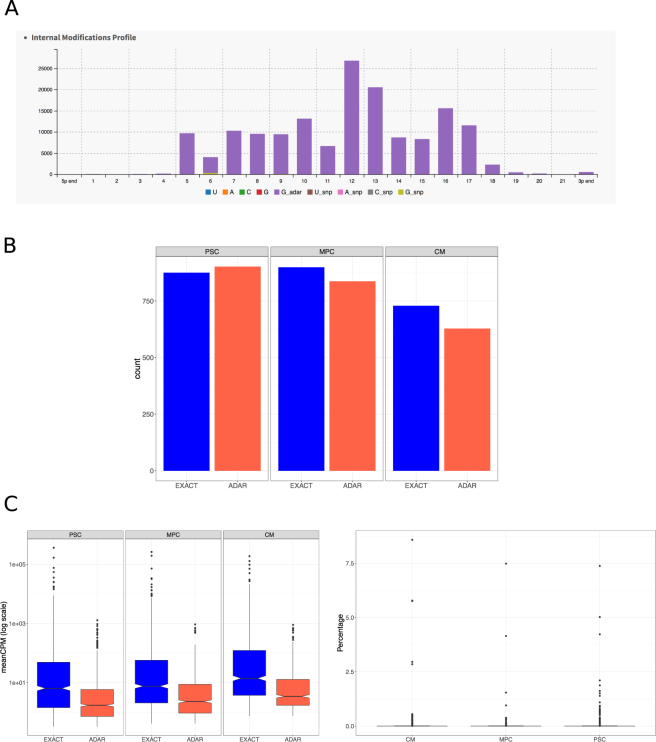
Adenosine-to-Inosine (ADARs) modifications in mature microRNAs. (**A**) ADARs occurs all along the sequence of microRNAs, including part of the seed sequence. Of note, they barely occur in the microRNA extremes. (**B**) Counts of all microRNAs with detected ADARs. There is a slight but statistically significant drop in the number of microRNAs with detected ADARS in CM compared to PSC. (**C**) Expression level of the ADAR modifications. On the left panel, overall expression (in CPM) of ADARs is compared against microRNA expression. There is no significant changes along differentiation. On the right panel, ADAR expression is depicted as percentage of expression of the mature microRNA. Overall, ADARs are a low percentage of mature forms, except for a few cases.

MicroRNA modifications also occur by a different mechanism. A-to-I edition or ADAR (*adenosine deaminase acting on RNA*) are identified in short RNAs. These modifications are believed to alter microRNA degradation^24,25^. We used the Chimira software from the EBI institute^21^ to identify ADAR modifications and found consistent alterations in the microRNA sequence. ADAR modifications were globally found across all microRNA hairpins, in a similar fashion as isomiRs, on each extreme as well as in the internal structure of the mature microRNA (Fig. 3A). Proportionally, we found a significantly greater number of ADAR modifications in the PSC population compared to CM (p = 0.014, Fig. 3B). When we analyzed the expression of the ADAR-modified microRNAs, they were similarly expressed across differentiation (Fig. 3C). However, the expression level was low, as it has been described previously for ADARs (Fig. 3D)^25^.

Altogether, these results suggest that isomiRs may have a functional significance in pluripotent stem cells and during the cardiac differentiation. Their equivalent number of reads to the exact sequence microRNAs and their different targeting specificities support this concept. With the study and incorporation of the isomiRs in the analysis, we determined a miRNAome during cardiac differentiation far richer and more complex than previously thought.

### microRNA genomic cluster and family analyses

Even though unique microRNAs are associated with specific cell functions, it is increasingly clear that miRNAome acts in concert. Non-physiological experiments may mask the real role of each microRNA in the differentiation process, such as a forced increased expression of a single microRNA to supra-physiological levels. We think a better understanding of the function of the miRNAome is then given by analyzing groups of microRNAs as a whole. microRNAs are usually grouped based on two features: genomic clustering, based on the genomic proximity (arbitrarily defined in mirBase as those microRNAs located within 10,000 bases), and seed sequence, based on sequence similarity. In many cases the genomic distribution of the microRNAs is not random and it has been found that some of them group in clusters in specific genomic regions and aggregate in islands of microRNAs. It is likely that these islands share regulatory machineries, although little is known about them. However, all the microRNAs that share the same seed sequence constitute a microRNA family, and are expected to regulate similar gene targets. We therefore analyzed the expression profiles of the microRNAs during cardiac differentiation considering clusters and families.

**Figure 4:**
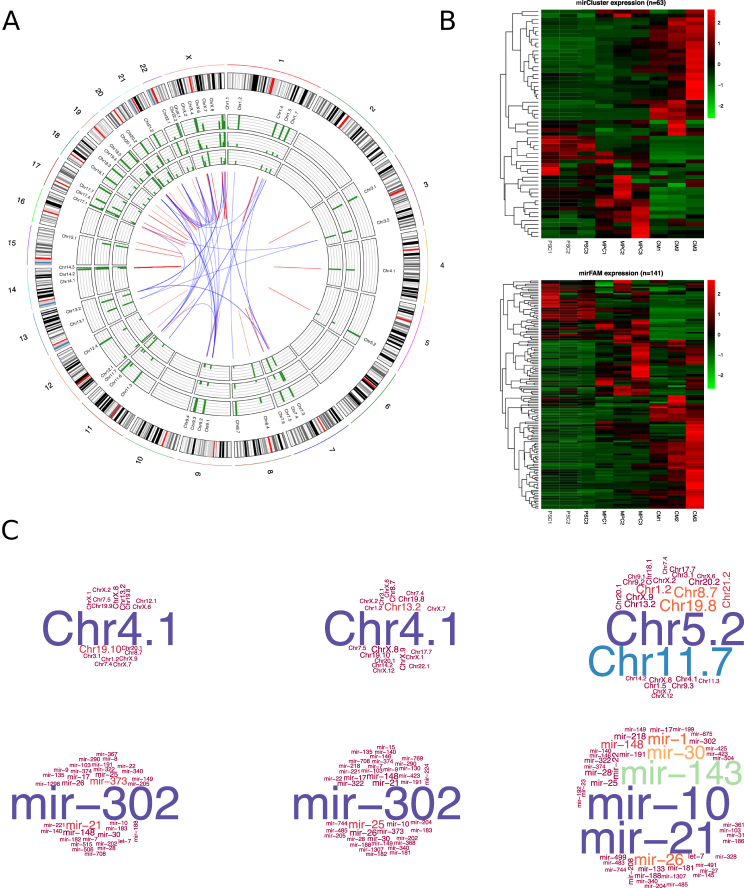
Analysis of microRNA expression based on Clusters and Families. (**A**) The Circos plot represents the genome localization of the microRNA clusters. In the outer circle are represented all the chromosomes as a reference for the position of the clusters. The expression level of microRNA clusters in PSC, MPC and CM are shown in the external, middle and inner circles, respectively. In the center red and blue lines represent interconnections of families and clusters, for clusters with microRNAs of only one family or members of more than one family, respectively. (**B**) Expression level of microRNA clusters and microRNA families are shown in the heatmaps upper and lower, respectively. The analysis was done based on the microRNA expression of the three biological replicates of PSC, MPC and CM. (**C**) The list of microRNA clusters and families identified in the three cell populations are represented in the top and bottom word clouds, respectively. Bigger letter represents the most highly expressed clusters/families.

A first insight of these analysis is shown in Fig. 4A. The Circos plot shows the genomic distribution of the microRNA clusters. Each ring contains bar graphs corresponding to the average expression of the microRNAs clusters of PSC (inner circle), MPC (middle circle) and CM (outer circle). In the center region, the linking lines represent the association between clusters and families. As it can be seen, many clusters are composed of microRNAs from different families (blue crossing-lines in the center). For example, four families (mir-17, mir-19, mir-25 and mir-363) are located at three different clusters (at cluster 7.4, cluster 13.2, and cluster X.8). Many other clusters are composed by a single family (red lines), as for example the mir-302 family/cluster (cluster 4.1).

By comparing heatmaps of both cluster and family groups, it can be seen that they were quite similar (Fig. 4B). This finding highlights the fact that clusters and families share a similar dynamic behavior during differentiation, even though they may be spread along the complete genome. Expression level for clusters and families are shown in the Supplemental Files 9 and 10. The analysis of similarities between cell populations by a sample distance heatmap (Supplemental Fig. 7A), showed a high similarity between the cluster and family. We also performed principal component analysis on the microRNA profiles to assess the differences and similarities between the three cell populations and these results support the concept that microRNA families and clusters are very similar, therefore being more adequate to discriminate between cell populations than individual microRNAs (Supplemental Fig. 7B).

We generated word clouds with cluster and family expression for a graphical representation (Fig. 4C). The well-characterized miR-302 family (mainly represented by miR-302a, miR-302b, miR302c, miR302d) is overwhelmingly expressed in both PSC and MPC. There are also some other families in the PSC population, such as miR-290, miR-373, miR-21 and miR-25. In the case of MPC, the most distinctive family apart from the miR-302, is the miR-25 (miR-25, miR-92a and miR-92b). Finally, the most expressed cardiac families are miR-21, miR-10 (miR-10a, miR-10b, miR-99a, miR-99b, miR-100, miR-125a and miR-125b), miR-143 (composed only by miR-143), miR-30 (miR-30a, miR-30b, miR-30c and miR-30d), miR-26 (miR-26a and miR-26b) and miR-1 (miR-1 and miR-206). Interestingly, except for the well-known cardiac microRNA miR-1, the miR-21 and miR-143 families, the rest of them have not been associated before with the cardiac lineage. The cluster word clouds complemented the family word clouds; hence, Chr4.1 (where mir-302 family is located) was highly represented in PSC and MPC. Chr5.2 (mir-143 and mir-145) and Chr11.7 (part of the let-7 family) were highly represented in CM.

**Figure 5:**
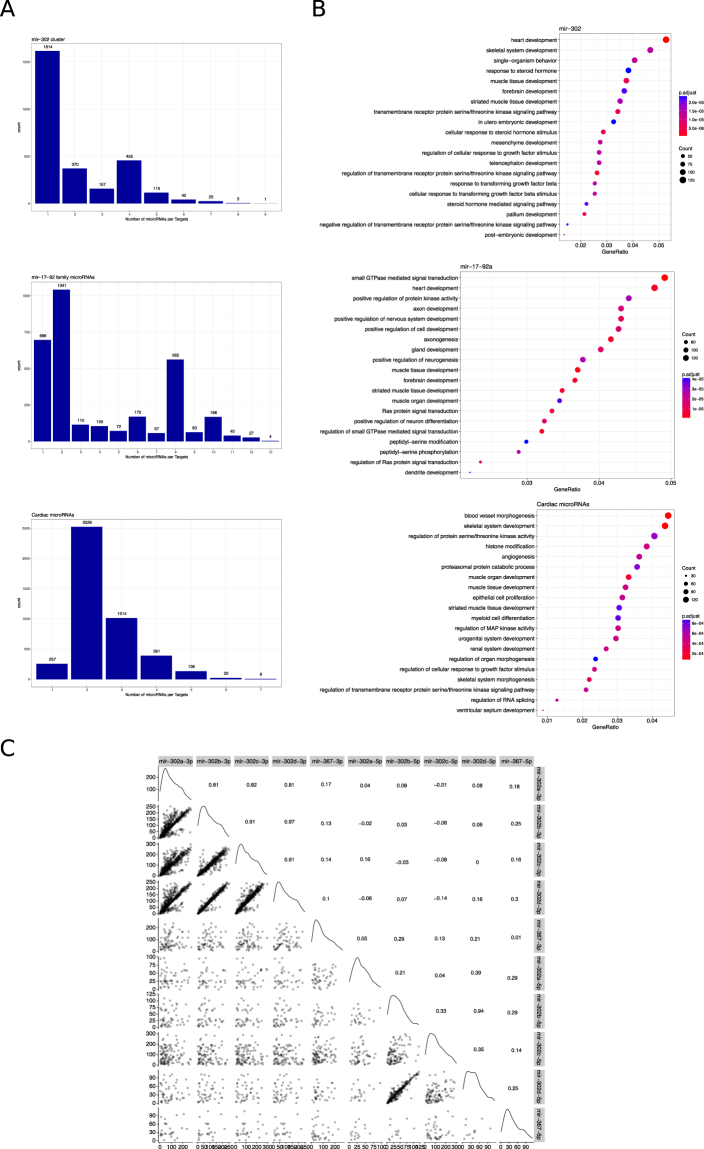
Target genes analysis from microRNA clusters and families. (**A**) Graph bars depict the number of genes regulated by the number of microRNAs from clusters Chr 4.1 (miR-302), cluster miR-17-92 and cardiac microRNAs (miR-1, miR-133, miR-208, miR-490, miR-206, miR-499 and miR-143). (**B**) Gene Ontology (GO) terms from each group of microRNAs are shown in the dot plots. Dot size represents the number of genes included in each GO term and the colorimetric scale is based on the p-value. Gene Ratio represents the number of genes per the total number of genes in each GO term. (**C**) Correlation between the scores of the predicted targets were analyzed in pairwise for each member of the miR-302 cluster and represented in the scatter plots. Correlation scores are shown in the upper right portion of the graph and in the diagonal line are shown the density plots.

We then studied the predicted targets by gene ontology of three cluster/families of microRNAs specific to each stage of the differentiation process to evaluate the pathways they might be regulating during cardiac differentiation. We chose the miR-302 family, miR-17/92 family and a group of microRNAs highly expressed in CM population. Figure 5A shows the number of predicted targets that are regulated by one or more microRNAs from each group. Many genes are under the control of more than one microRNA, which illustrates the cooperative role that microRNAs have in gene silencing. We next analyzed the Gene Ontology (GO) terms associated to the predicted target genes to study the putative molecular processes regulated by these groups of microRNAs (Fig. 5B). For both miR-302 and mir-17-92a families many GO terms were related to developmental processes (see complete lists in Supplemental Files 11 and 12). It is worth to note that heart development ranked at the top in both families. As both microRNAs families were highly expressed in PSC, it was expected that these regulated GO pathways were expected. When microRNAs where individually analyzed, however, GO terms did not clearly relate to embryonic development (see an example in Supplemental Fig. 8), emphasizing that the addition of all family members predicts a highly significant effect over embryonic development, particularly cardiac differentiation. We then analyzed the GO terms associated to targets of the microRNA families highly expressed in CM, including miR-1 (mir-1 and mir-206), miR-133 (miR-133a and b), miR-208 (miR-208a and b), miR-490, miR-499 and miR-143. The analysis revealed that these microRNAs might be downregulating genes related to different cell lineages derived from the mesoderm germ layer, such as striated muscle development, skeletal system development and urogenital system development (see complete list in Supplemental File 13). This is in line with the concept that microRNAs act as key regulators during early development and orchestrate the gene expression that leads to the establishment of cell identity, and in the specific case of CM, inhibiting the development of other mesoderm cells.

Additionally, we studied the correlation between the target affinity scores of the miR-302 cluster/family for their common targets (see above). In Fig. 5C the upper part of the matrix shows the correlation scores between pairwise comparisons of miR-302 members. As it can be observed, -3p microRNAs had the highest correlation between them than -5p microRNAs, except for miR-302b-5p and miR-302d-5p. This observation implies that members of this cluster act cooperatively in the control of gene expression, but redundancy between them is not equal for all the members of the cluster. This latter observation suggests that despite the overlapping of gene targets, each microRNA from miR-302 cluster interacts with different affinities to their target RNAs. Similar findings for the mir-17-92a cluster/family and the cardiac microRNAs can be seen in Supplemental Fig. 9. Hence, these findings support the previous concept that embryonic microRNA cluster/families team up to inhibit different developmental pathways.

### Cardiac associated microRNAs

**Figure 6:**
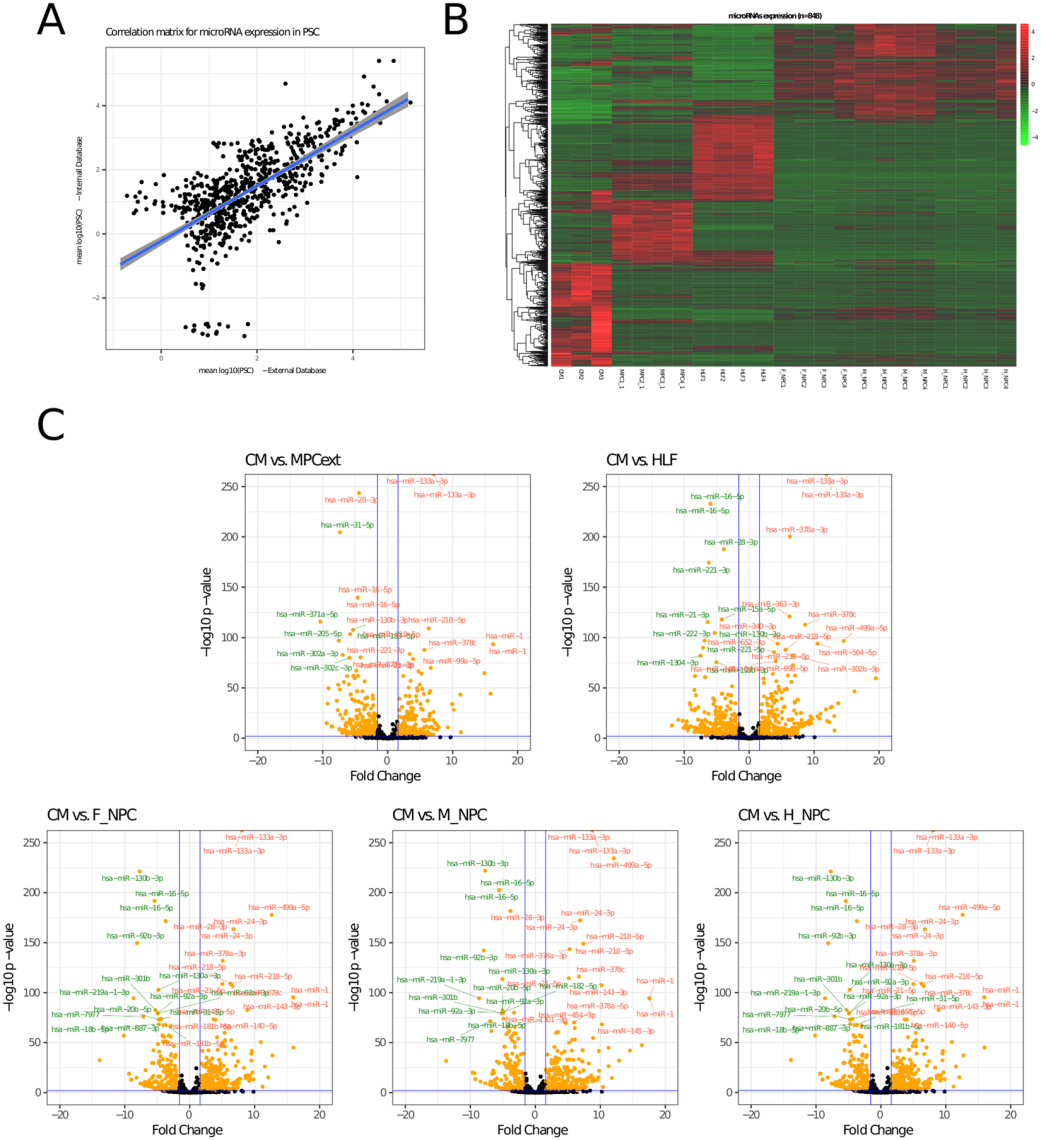
Comparison of microRNA expression with external data bases. (**A**) Scatter plot of microRNA expression in PSC (internal database, y axis) and an external database (x axis). (**B**) Heatmap depicts expression level of microRNAs from 6 cell lines including CM, Mesenchymal progenitor cells (MPC), Human lung fibroblasts (HLF), Forebrain Neural progenitor cells (F_NPC), Midbrain Neural progenitor cells (M_NPC) and Hindbrain Neural progenitor cells (H_NPC) with their respective replicates. (**C**) Differential expression of microRNAs between CM and the non-cardiac cell lines (external data) are depicted in the Volcano plots. Blue lines delineate the cutoff for microRNAs significantly upregulated (red) and downregulated (green) with a log2 FC ≥ 3 and p ≤ 0.01. Names show the most extreme microRNAs.

Comparing microRNAs expressed in cardiac cells to pluripotent stem cells does not confirm that those differentially expressed microRNAs are exclusively of cardiac cells. Hence, we compared the miRNAome of cardiac cells against other differentiated cells. An external small-RNA-Seq database was analyzed with the same methods (GEO accession number GSE68189)^26^. These samples were mostly from the same embryonic stem cell background (H9), and includes mesenchymal stem cells derived from PSC and three neural differentiated cells (fore-, mid-, and hind-brain). This analysis also included a cell line derived from human embryonic lung fibroblasts. There was a correlation of the miRNAome between PSC samples, hindered by some microRNAs with low expression in either group (Fig. 6A). A heatmap of the microRNA expression profiles of all cell populations showed a marked difference between the differentiated cells (Fig. 6B). As it can be seen, a microRNA expression pattern was distinctive. Pairwise comparisons could be observed in the volcano plots (Fig. 6C). Supplemental Files 14–18 list the microRNAs down-regulated and up-regulated in each comparison between differentiated cells. Finally, we analyzed the expression of microRNAs comparing all cell lines simultaneously in order to build a cardiac miRNAome. In Table 3 we show three different microRNA groups. The first two columns show the top ranking microRNAs with highest absolute expression in CM. The third and fourth columns show that those microRNAs are significantly up regulated in CM compared to all other cell lines analyzed, including PSC and differentiated cells. Finally, the last two columns show those microRNAs uniquely expressed in CM (that is, zero expression was found in any other cell population).

**Table 3:** miRNAome expressed in cardiac cells.

microRNA (all)	mean	microRNA - DE	mean (DE)	microRNA - unique	mean (Unique)
hsa-miR-21-5p	199059	hsa-miR-143-3p	135432	hsa-miR-1-3p	48933
hsa-miR-143-3p	135432	hsa-miR-100-5p	74723	hsa-miR-499a-5p	9598
hsa-miR-100-5p	74723	hsa-miR-1-3p	48933	hsa-miR-208a-3p	4940
hsa-miR-1-3p	48933	hsa-miR-30d-5p	29289	hsa-miR-675-5p	686
hsa-miR-30d-5p	29289	hsa-miR-99a-5p	17254	hsa-miR-208b-3p	621
hsa-miR-148a-3p	28649	hsa-miR-22-3p	14910	hsa-miR-133b	348
hsa-miR-26a-5p	24070	hsa-miR-125a-5p	14101	hsa-miR-133a-5p	199
hsa-miR-26a-5p	24021	hsa-miR-27b-3p	10936	hsa-miR-585-3p	175
hsa-miR-99b-5p	21955	hsa-miR-125b-5p	10647	hsa-miR-1248	104
hsa-miR-99a-5p	17254	hsa-miR-499a-5p	9680	hsa-miR-509-5p	88

This table summarizes the most important microRNAs describing the cardiac cell population. First two columns describes the most expressed microRNAs and its readings, taken from the general analysis. Third and fourth columns (DE, differential expression) describes those microRNAs most significantly differentially expressed compared to other cell populations. Finally, fifth and sixth columns (unique) describes those microRNAs with no expression in any other cell population analyzed.

## Discussion

Cardiac differentiation from human pluripotent stem cells presents dynamic changes in the landscape of microRNA expression. We analyzed the microRNA signature in order to establish the identity of pluripotent stem cells (PSC), early mesoderm progenitor cells (MPC) and cardiomyocytes by small RNA-sequencing. Our analysis revealed that almost 700 microRNAs are differentially expressed in the three cell populations and they have specific expression patterns according to the differentiation stage. Therefore, each cell population has a characteristic miRNAome that has an important regulatory role in the establishment of cardiac lineage. Two additional concepts were explored in this paper. First, families and clusters of microRNAs work in harmony through the dynamic stages of differentiation. Second, minor but numerous isoforms of the microRNAs may take a significant role in the regulation of this differentiation.

MicroRNAs form a complex regulatory network important in the control of gene expression during human embryonic development. There is increasing evidence of the role they have in the post-transcriptional regulation of key developmental genes^27,28^. The complexity that characterizes the way microRNAs work is partially explained by the fact that one mRNA is simultaneously targeted by numerous microRNAs and each microRNA regulates several mRNA at the same time. Moreover, the high sensitivity in new sequencing technologies allowed the detection of microRNA isoforms named isomiRs. These variations imply an extra level of regulatory complexity as each microRNA can have a myriad of isomiRs that might strengthen and expand the regulation of the target RNA messengers. The isomiRs were initially considered errors from either the sequencers or softwares used in the analysis of the results^12^, whereas today there is evidence that supports their functional relevance^29,30^. We described an extensive network of isomiRs during cardiac differentiation. Most abundant isomiRs were those with variations in the 3′-end. Nonetheless, we also identified several isomiRs with changes in the sequence at the 5′-end. These modifications in the 5′-end presumably have an impact in mRNA recognition. Despite this, it has been proposed that heterogeneity in the 3′-end might have important implications in the microRNA stability and AGO loading affinity^12^, hence having a potential effect in the microRNA regulatory activity. Importantly, we found no evidence that differentiation affects isomiRs formation from the exact sequences. We then analyzed in more detail the microRNA miR-302a and their isomiRs, revealing the case of a particular microRNA being less abundant than its isomiRs and with a repertoire of predicted targets considerably different for most of the discovered 5′-isomiRs. We identified 415 novel predicted targets for the 5′-isomiRs of the miR-302a-3p and 242 for the 5′-isomiRs of the miR-302a-5p, both highly expressed in the PSC. Furthermore, the Gene Ontology analysis of common and different transcripts targeted uncovered new cellular processes that are implicated principally in cell differentiation, which would not have been identified if the 5′-isomiRs were not considered in the analysis. Our data is in line with the work^13^ that demonstrates that the incorporation of the isomiRs increases the accuracy of target prediction.

MiRNAome analysis has generally overemphasized individual microRNAs expression profiles. However, the sequence of many microRNAs are very similar, particularly in the seed region, which has been used to group them into families. Many microRNAs are also typically found in genomic clusters, arbitrarily defined as microRNAs separated by no more of 10,000 bases. We then believe that these features should be considered when analyzing a miRNAome, as families and clusters may exert their function over common genes or common molecular pathways. Therefore, we determined the dynamic expression profiles of 63 microRNA clusters and 141 microRNAs families that contribute to cell fate decision during cardiac differentiation. The most expressed microRNA family in PSC and MPC was miR-302, which also corresponds to a cluster we named Chr4.1 (first one found in Chormosome 4). This family/cluster has been extensively studied in previous works^31,32^ and it is of great importance in the maintenance and induction of pluripotency. In addition, there is another highly regulated cluster known as C19MC located at chromosome 19 (Chr19.10). This cluster is one of the largest found cluster in primates and has been suggested in a recent work to regulate apoptosis in pluripotent stem cells^33^. This cluster includes the family miR-515 but also miR-373 and miR-290. Both families are also up regulated in PSC. Another interesting upregulated cluster in both PSC and MPC is located on chromosome 13 (Chr13.2), which includes members of the families miR-17, miR-19 and miR-25. These microRNAs have an important role in normal tissue development but also an oncogene role in different cell types^34,35^. Another family expressed in MPC that stands out from the rest is the miR-26 family (miR-26a and b), which is also related to tumorigenesis^36^. These groups of microRNAs could be associated with the epithelial to mesenchymal transition occurring in the development of the mesoderm progenitor cell, which is found in developing tumors. Lastly, we looked at the up-regulated microRNA clusters and families in CM. The five clusters with higher expression level in CM are Chr5.2, Chr11.7, Chr19.8, Chr8.7 and Chr1.2. By large, these clusters are formed by members of the miR-143 family, let-7 family and miR-30 family. Consistently with previous works, miR-143 regulates smooth muscle differentiation and let-7 family decreases with early differentiation and is up regulated in adult cardiac tissue^37,38^.

Gene ontology analysis of the clusters/families in the cell groups showed that there are shared processes regulated such as heart development or muscle development, and most GO terms are related to embryonic development. Notably, there is a progressive narrowing (in terms of embryo development) on the range of cellular processes regulated once the cells have reached a more mature stage. In PSC there are more GO terms related to a wider variety of cell lineages like heart, skeletal system, muscle tissue, forebrain, striated muscle tissue, *in utero* embryonic development, mesenchymal, telencephalon and pallium development. On the contrary, GO terms related to genes silenced in MPC and CM are mostly general cellular processes, neural and muscle development. This observation suggests that once the cells have reached a mature phenotype,as in the case of a cardiac phenotype, the up-regulated microRNAs control gene expression of very specific pathways. Moreover, the score correlation of predicted targets of the family miR-302 illustrates the synergistic effect that these microRNAs have. Finally, it is remarkable that the individual GO analysis of a microRNA does not usually show enough gene targets of embryo development. It is necessary to include all family members (as in the case of mir-302) in order to reach significance in the developmental pathways. Altogether, these results suggest that microRNAs act cooperatively in silencing key genes during cardiac differentiation, emphasizing the importance of a genome-wide analysis, in addition to considering the expression profile of families and clusters of microRNAs.

Finally, we compared our results with published data of microRNAs in different cell types to narrow the list of microRNAs that specifically contribute to the miRNAome of the CM (Box 1). Our analysis showed that there are 848 microRNAs differentially expressed between the six cell types. In line with previous observations, each cell type has a specific signature of microRNAs implying the importance of microRNAs in cell fate decision. Lastly, in our analysis we were able to identify a group of microRNAs that are significantly up regulated in cardiac cells and a short series of microRNAs that are unique to CM. This finding is relevant, as they confer CM with a specific signature. Some of these microRNAS have been previously reported and are well-known in the context of cardiac differentiation, such as hsa-mir-1-3p, hsa-mir-499-5p, and the mir-208 and mir-133 families^39,40^. However, other microRNAs have not been described in cardiac differentiation, including hsa-mir-675-5p, hsa-mir-585-3p, hsa-mir-1248 and hsa-mir-509-5p. To what extent these microRNAs are relevant in cardiac differentiation still remains to be determined.

In conclusion, we determined the microRNAs that are involved in cell lineage specification during human pluripotent stem cells differentiation towards mesoderm and cardiac cells. These results expand the knowledge of microRNAs and their isomiRs in the regulation of genes and molecular mechanisms important during cell fate decision. Due to the high complexity of the miRNAome, a genome-wide approach is mandatory for a better comprehension of the role these small non-coding RNAs have and functional studies that include all microRNAs members of families/clusters will be necessary to further understand their proper relevance in the differentiation process.

## Methods

### Cell Culture and cell differentiation

Human embryonic stem cells (H9-hTnnTZ-pGZ-D2) were obtained from WiCell and maintained in co-culture with irradiated primary mouse embryonic fibroblasts (MEFs) in the presence of FGF (8 ng/ml; Thermo Fisher Scientific) under conditions described by the supplier, as previously reported^41^. This cell line has been genetically modified in WiCell to express both GFP and Zeocin resistance under the promoter of cardiac troponin T. All experiments were carried out in accordance with relevant guidelines and regulations. All cultures were maintained at 37 °C in a saturated atmosphere of 95% air and 5% CO2. To induce mesoderm differentiation and obtain the early mesoderm progenitor (MPC) we followed a previously described protocol by Evseenko *et al*. (Supplementary Fig. S1A)^15^. Briefly, cells grown in iMEFs were detached using TrypLE^TM^ Select (1X; Thermo Fisher Scientific) and cultured on 6-well plates pre coated with Geltrex Matrix (Diluted 1:1000 from 15 mg/ml; Thermo Fisher Scientific) with mTesR (StemCells Technologies). Cells were grown in these conditions until they were 60–80% confluent (typically 3 days). To induce differentiation medium was replaced with StemPro®-34 SFM (Thermo Fisher Scientific) supplemented with the following proteins: human bone morphogenic protein 4 (BMP4), human vascular endothelial growth factor (VEGF) and fibroblast growth factor 2 (bFGF) (all at 10 ng/ml; Thermo Fisher Scientific) with the inclusion of Activin A only at the first day (10 ng/ml). At day 3.5 after mesoderm induction, the MPC were isolated by fluorescence-activated cell sorting (FACS) using PerCP/Cy5.5 anti-human CD326 and APC anti-human CD56 antibodies from Biolegend. For CM differentiation, embryoid bodies (EB) formation was forced by centrifugation of hESC (H9-hTnnTZ-pGZ-D2) in 96-well plates V-bottom with mTeseR supplemented with the growth factor BMP-4 (10 ng/ml; Thermo Fisher Scientific). After 24hs, EB were cultured in suspension with StemPro®-34 SFM (Thermo Fisher Scientific) supplemented with BMP-4 (10 ng/ml), bFGF (5 ng/ml) and Activin A (3 ng/ml) during 4 days. At day 4, EBs were collected and passed to adherent plates precoated with gelatin (0,1%; Sigma Aldrich) and the inhibitor Wnt response factor (IWR-1) was added to the medium (5 microM; Sigma-Aldrich). Between day 5 and day 21 cells were cultured in StemPro®-34 SFM supplemented with VEGF (5 ng/ml), bFGF (10 ng/ml) and IWR-1 (5 microM). At day 11 CMs were purified with two pulses of Zeocin for 72hs (75 microg/ml), allowing 2–3 days to recover between pulses. At day 21 the percentage of cells GFP+ was analyzed by Flow Cytometry. At this stage, cardiomyocytes are differentiated up to the point of presenting spontaneous beating activity.

### Small RNA-Sequencing samples preparation

The small RNA molecules were extracted with mirVana^TM^ miRNA Isolation Kit (Ambion, Thermo Fisher Scientific) following manufacturer’s protocols. Small RNA libraries were prepared with 200 ng of RNA and using the NEBNext Small RNA Library Prep Set with modified adaptors and primers compatible for Illumina (New England Biolabs) and cleanup and size selection (50pb) was made by gel electrophoresis following manufacturer’s protocols. Sequencing was carried out at the TCGB Resources - UCLA Path & Lab Med using an Illumina HiSeq. 2500, in one lane and single read sequencing. Sequencing datasets are deposited in GEO (GSE108021).

### Small RNA-sequencing results analysis

Processing and annotation of sequences based on identity to known transcribed RNAs or as novel miRNAs was performed by Novogenix BSU using the CAP-miRSeq (v1.1) pipeline^42^. Briefly, pretrimmed and posttrimmed reads were screened for quality, specificity of mapping, and contaminant sequences using FASTQC. Then prior to alignment, low-quality bases, homopolymer sequences, sequences matching the first 3 bp, and the reverse complement of the adaptor sequences for Illumina were trimmed using Cutadapt (v1.13) with a maximum error rate of 0.1%. The trimmed reads were then aligned using Bowtie and HTSeq^43,44^ for the reads counting and then the reads were mapped to the human genome reference (GRCh38) with miRDeep2 mapper and to miRbase (v21) with miRDeep2 module which allowed us to identify known and novel microRNAs^45^. Normalization of read number was carried out as Counts per Million within each sample. For differential expression analysis we used R package DESeq. 2, using raw data as inputs. IsomiR analysis was done mainly with miraligner/isomiR (v1.6)^20^, but also with the isomiR-SEA^22^ and Chimira web interface^21^. Gene target prediction of microRNA was done with miRNAtap from Bioconductor package, which includes the prediction algorithms of DIANA, Miranda, PicTar, TargetScan and miRDB. Targets of isomiRs were predicted using MR-microT from DIANA Tools^46^. Gene Ontology analysis was done with enrichGO from ClusterProfiler package in R^47^.

### Accession code

RNA-sequencing results can be found in GEO with the accession number: GSE108021.

## Electronic supplementary material

Supplemental Figures

Supplemental file 1

Supplemental file 2

Supplemental file 3

Supplemental file 4

Supplemental file 5

Supplemental file 6

Supplementary file 7

Supplemental file 8

Supplemental file 9

Supplemental file 10

Supplemental file 11

Supplemental file 12

Supplemental file 13

Supplemental file 14

Supplemental file 15

Supplemental file 16

Supplemental file 17

Supplemental file 18

Supplemental Video 1
