# Supplementary material for: Identification of the miRNAome of early mesoderm progenitor cells and cardiomyocytes derived from human pluripotent stem cells
Source: Sci Rep. 2018 May 23;8:8072. doi: 10.1038/s41598-018-26156-3 (PMC5966391; doi:10.1038/s41598-018-26156-3)
Supplement: Supplementary file 1 — Supplemental Figures [file 41598_2018_26156_MOESM1_ESM.pdf]

Identification of the miRNAome of early mesoderm progenitor cells and cardiomyocytes derived from human pluripotent stem cells

Ximena Garate[1], Alejandro La Greca[1], Gabriel Neiman[1], Carolina Blüguermann[1], Natalia L. Santín Velazque[1], Lucía N. Moro[1], Carlos Luzzani[1], Elida Scassa[1], Gustavo E. Sevlever[1], Leonardo Romorini[1], and Santiago G. Miriuka[1*]

[1]LIAN-CONICET, FLENI - Ruta 9 km 52.5 - Belen de Escobar, Provincia de Buenos Aires, Argentina
[*]smiriuka@fleni.org.ar

ABSTRACT

1 Supplemental Figures

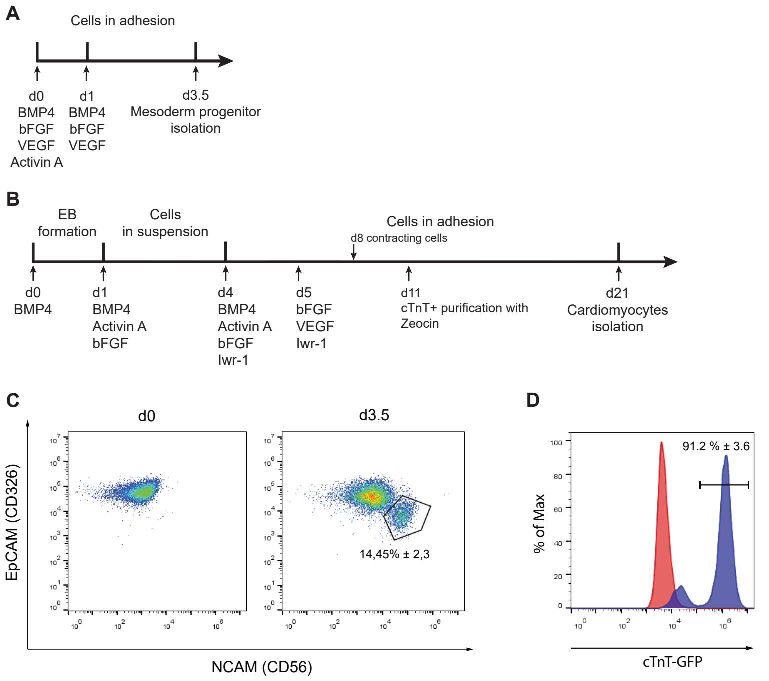

Figure 1. Supplemental Figure 1: Mesoderm and cardiac differentiation protocols of pluripotent stem cells. (A) Schematic of optimized PSC differentiation towards mesoderm progenitor cells (MPC). (B) Cardiac differentiation protocol scheme from PSC. EB, Embryoid bodies. (C) Flow cytometry of typical NCAM+/EpCAM- population (MPC) in cells differentiated from PSC at day 0 (left) and at day 3.5 (right) of differentiation. Mean % of three replicates $\pm$ SEM. (D) Flow cytometry of typical c-TnT+ (CM) at day 21 of differentiation from PSC, post Zeocin selection. Left peak represents c-TnT expression at day 0 of differentiation protocol (PSC population). Mean % of three replicates $\pm$ SEM.

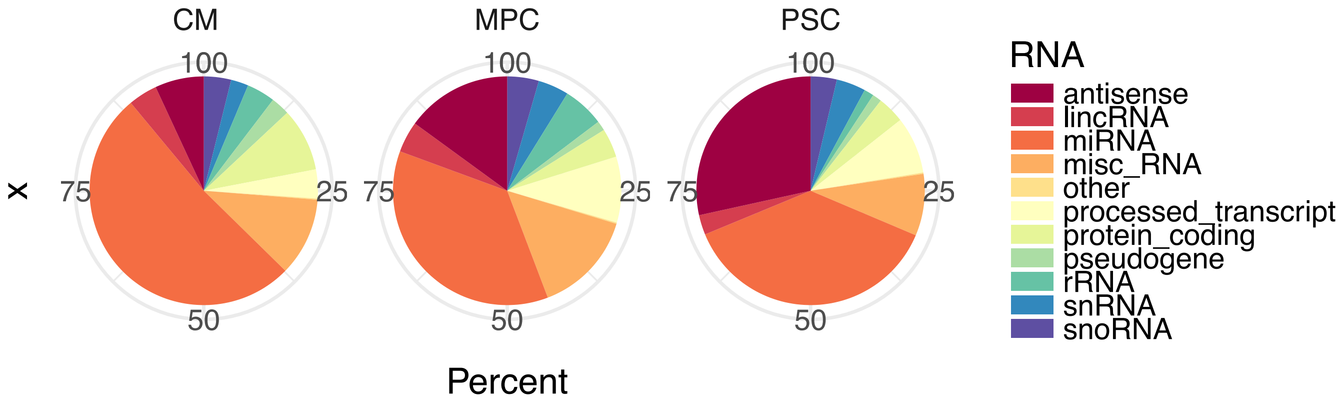

Figure 2. Supplemental figure 2: Abundance of the RNA molecules identified. Pie charts depicts the overall contribution of the different RNA molecules detected by high-throughput small RNA sequencing in three cell populations, PSC, MPC and CM. % represent the mean of three replicates for each cell population.

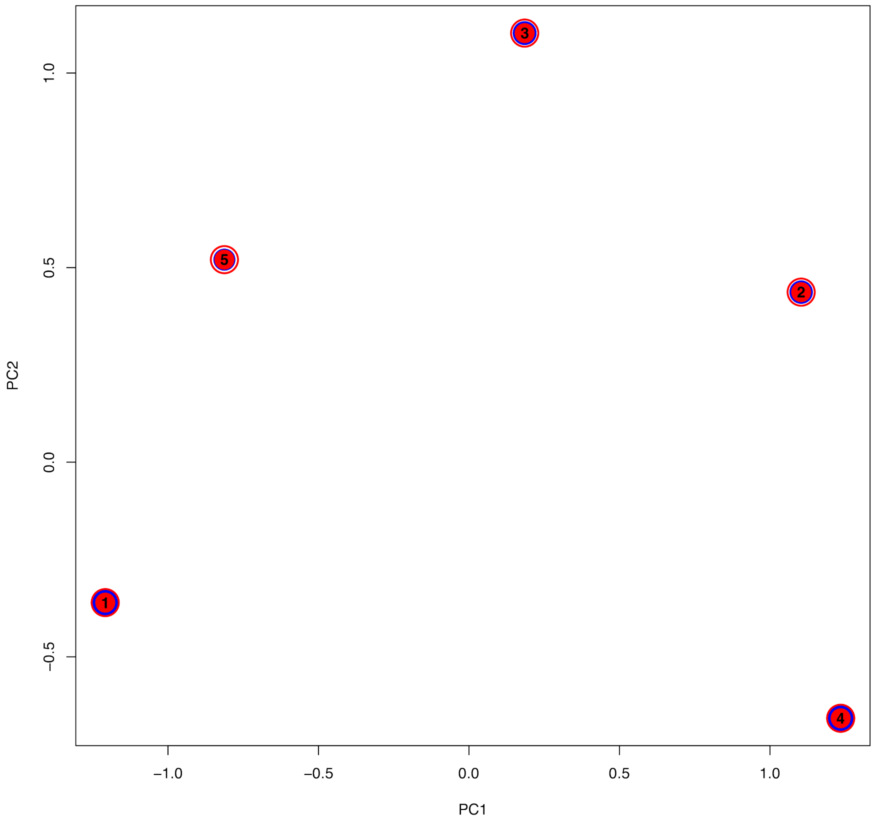

Figure 3. Supplemental Figure 3: Fuzzy plot groups distribution. 2D-PCA plot shows the relation between the microRNAs groups obtained with the fuzzy plots cluster adjusted. Each red dot represents one fuzzy group composed by microRNAs with a similar expression dynamic profile during cardiac differentiation.

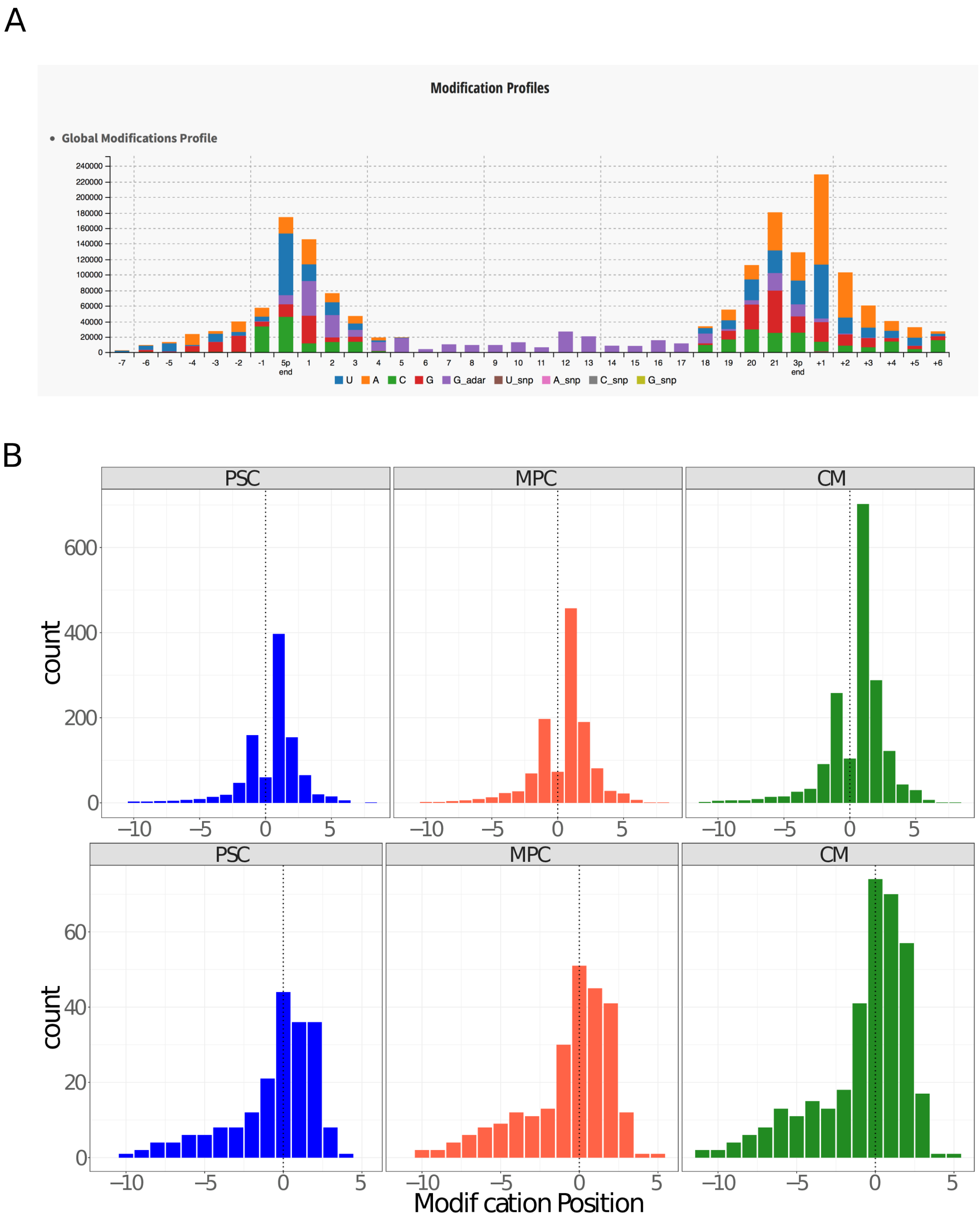

Figure 4. Supplemental Figure 4: Analysis of isomiRs using different softwares. (A) Graph bar shows the global modifications in the isomiRs sequences determined by the Chimira software. Each color represents the type of modification and the number of reads are represented according to the sequence position. (B) Graph bar depicts the number of isomiRs according to the sequence position of the modifications identified by using the Isomir-SEA software. Upper panel represents the 5'-end modifications and the lower panel the 3'-end variations determined for the three cell populations, PSC, MPC and CM. Position 0 corresponds to the nucleotide 22.

A

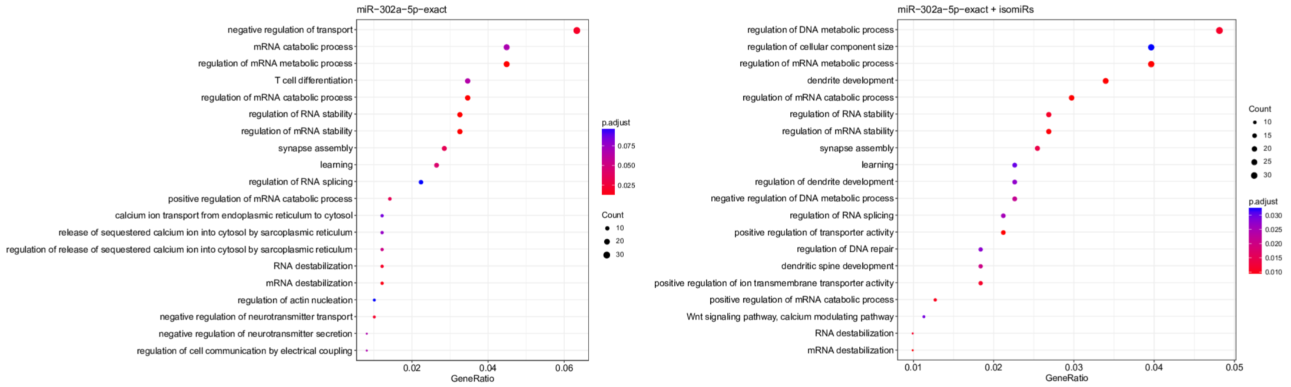

B

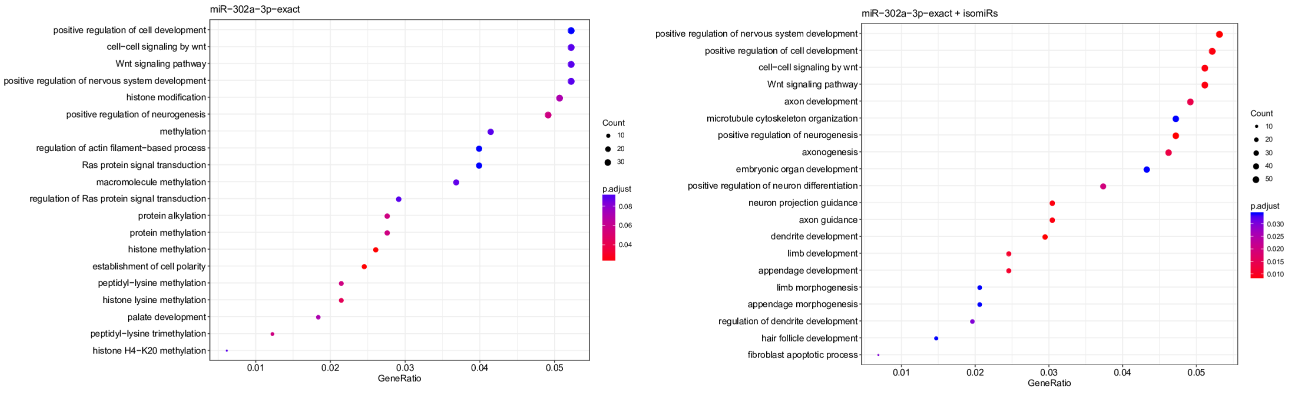

Figure 5. Supplemental figure 5: Gene Ontology analysis of predicted targets of miR-302a and their isomiRs. (A) The dot plot shows the most significant GO terms obtained when analyzing targets of the miR-302a-5p-exact (left) and targets of miR-302a-5p-exact plus isomiRs (right). (B) Most significant GO terms associated with targets predicted for miR-302a-3p-exact (left) and for miR302a-3p-exact plus isomiRs (right).

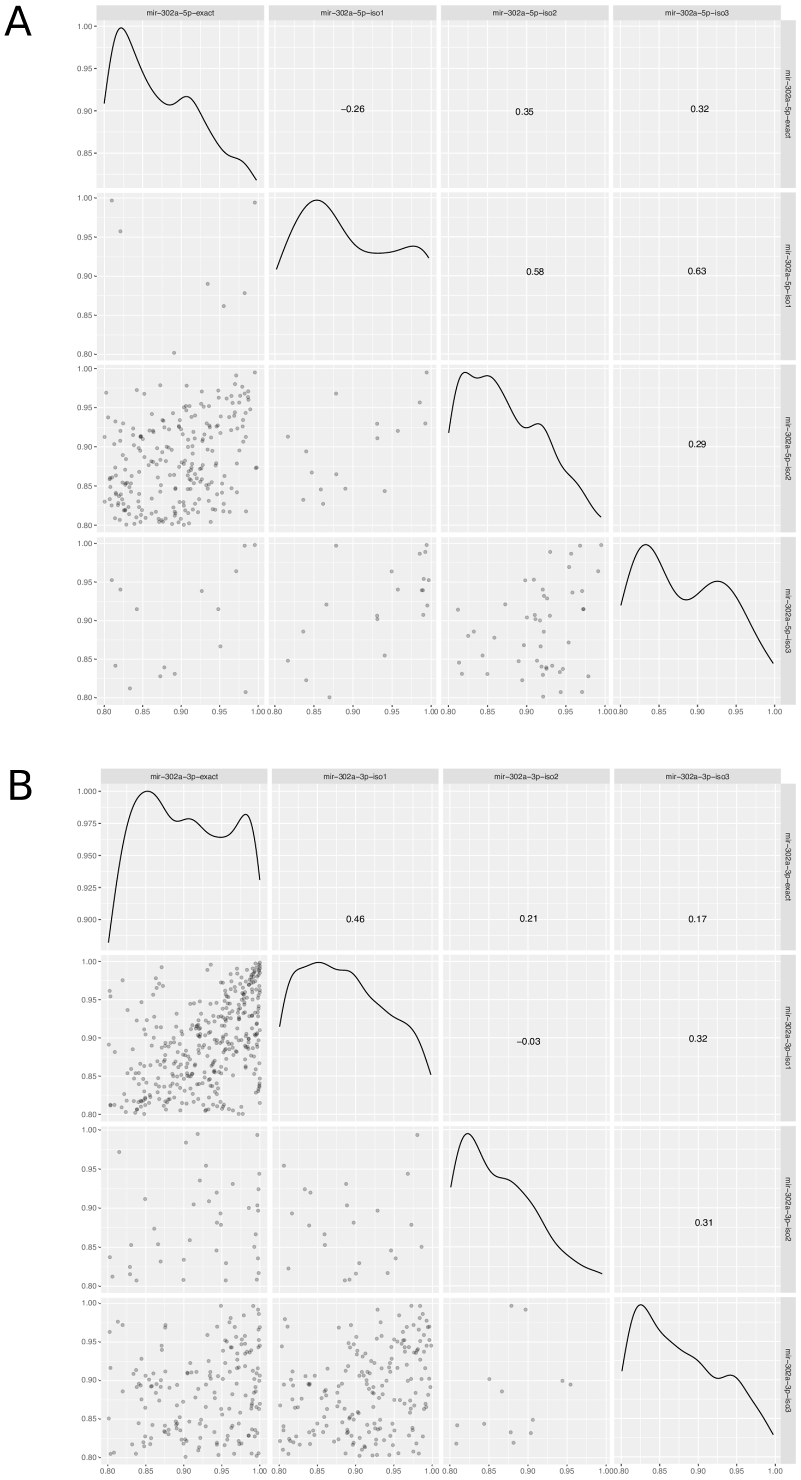

Figure 6. Supplemental Figure 6: Correlation analysis between targets of miR-302a and their isomiRs. (A) The scatter plots show pairwise analysis of the correlation between the scores of the predicted targets of miR-302a-5p and its isomiRs, and miR-302a-3p and its isomiRs (B). Correlation scores are shown in the upper right portion of the graph and in the diagonal line are shown the density plots

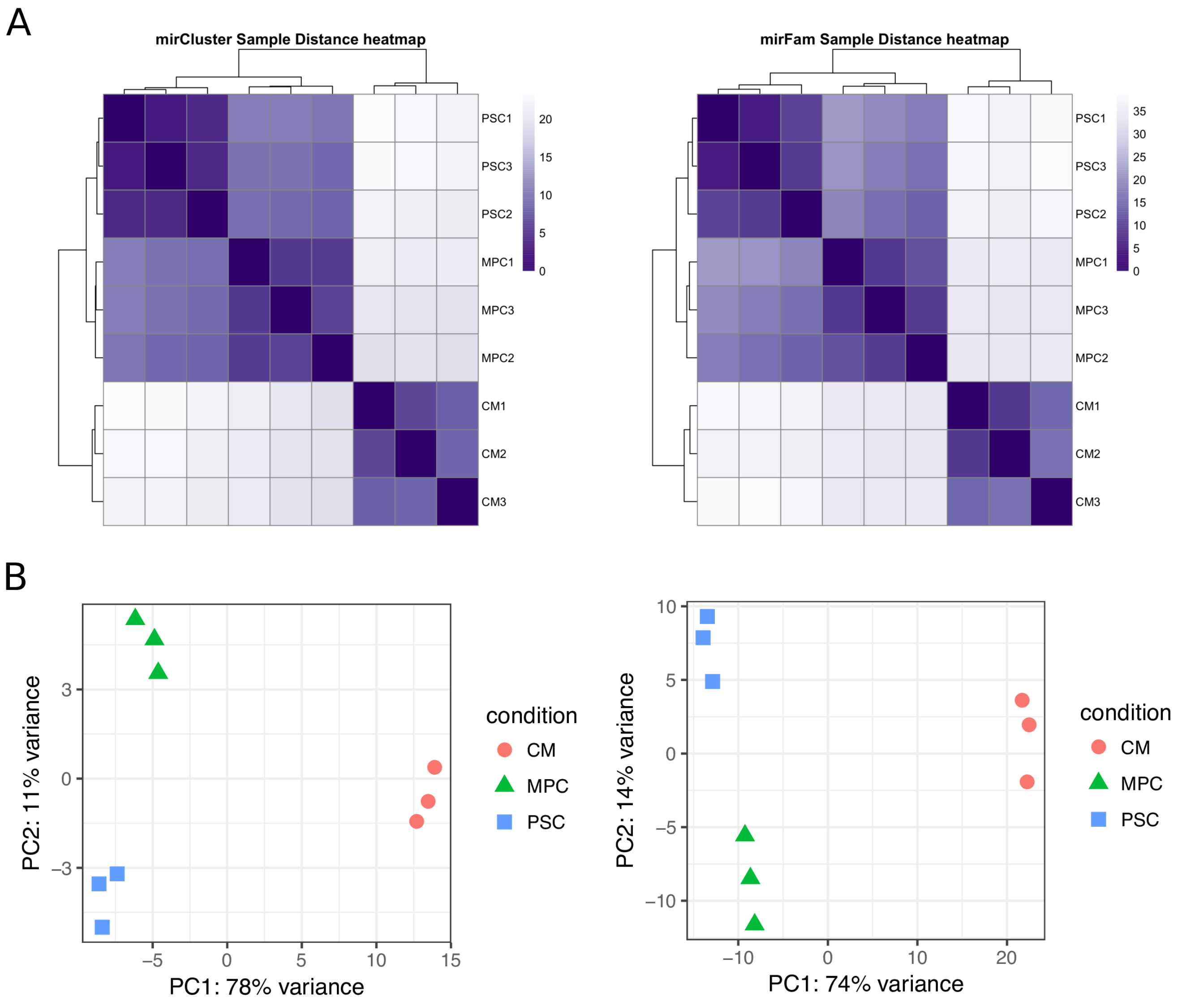

Figure 7. Supplemental figure 7: MicroRNAs family and cluster analysis. (A) The distance heatmaps depicts the differences between the three cell populations and their biological replicates based on the expression of microRNA clusters (left) and families (right). (B) 2D-PCA plots shows the distribution of the three cell populations and their respective biological replicates based on microRNAs clusters (left) and families (right) expression levels.

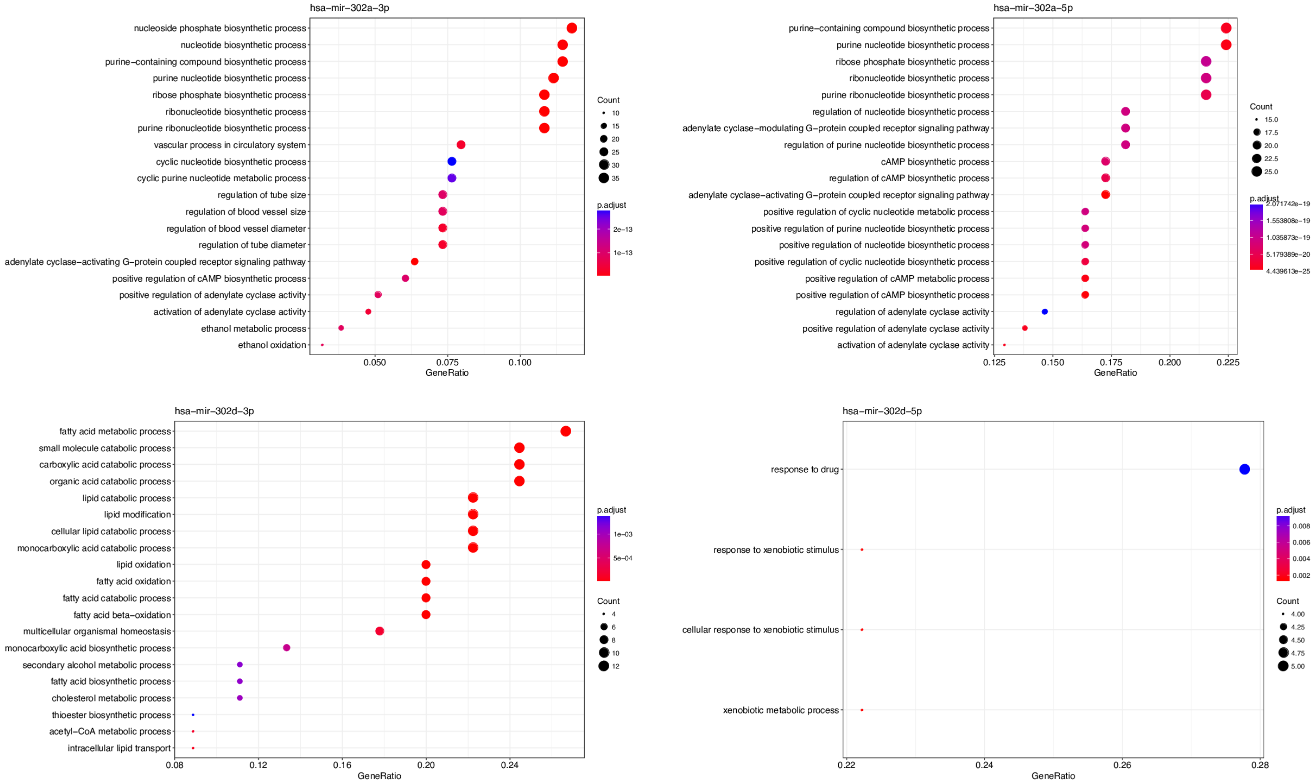

Figure 8. Supplemental figure 8: Gene ontology analysis of predicted targets of miR-302a-3/5p and miR-302d-3/5p. Dot plots shows the most significant GO terms determined for the predicted targets of miR-302a-3 (*upper-left*), miR-302a-5p (*upper-right*), miR-302d-3 (*lower-left*) and miR-302d-5p (*lower-right*).

A

B

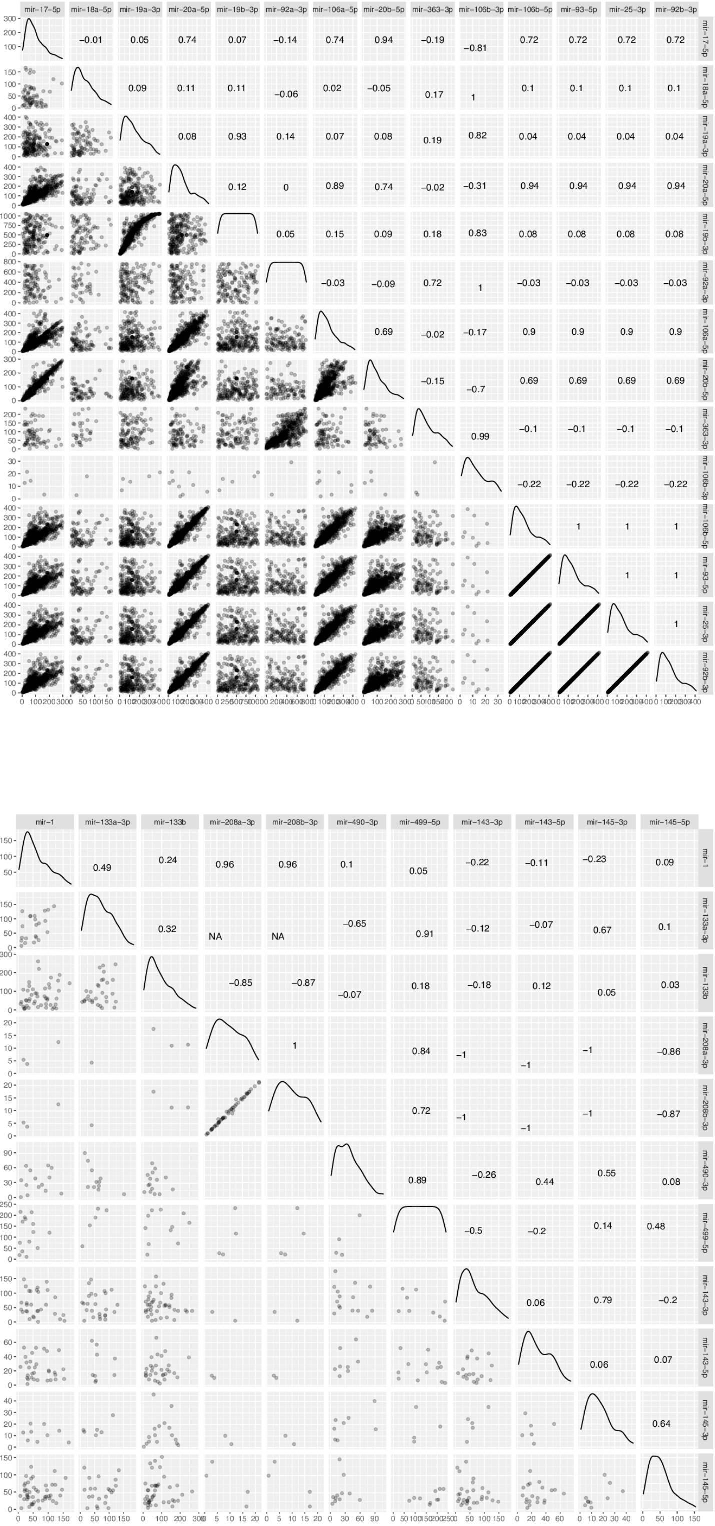

Figure 9. Supplemental figure 9: Target correlation analysis of mesoderm and cardiac microRNAs families/clusters. (A) The scatter plots show pairwise analysis of the correlation between the scores of the predicted targets for the miR-17/92a family/cluster and (B) for cardiac micoRNAs. Correlation scores are shown in the upper right portion of the graph and in the diagonal line are shown the density plots
