# Supplementary material for: Identification of the miRNAome of early mesoderm progenitor cells and cardiomyocytes derived from human pluripotent stem cells
Source: Sci Rep. 2018 May 23;8:8072. doi: 10.1038/s41598-018-26156-3 (PMC5966391; doi:10.1038/s41598-018-26156-3)
Supplement: Supplementary file 4 — Supplemental file 3 [file 41598_2018_26156_MOESM4_ESM.pdf]

Identification of the miRNAome of early mesoderm progenitor cells and cardiomyocytes derived from human pluripotent stem cells

Ximena Garate[1], Alejandro La Greca[1], Gabriel Neiman[1], Carolina Blüguermann[1], Natalia L. Santín Velazque[1], Lucía N. Moro[1], Carlos Luzzani[1], Elida Scassa[1], Gustavo E. Sevlever[1], Leonardo Romorini[1], and Santiago G. Miriuka[1*]

[1]LIAN-CONICET, FLENI - Ruta 9 km 52.5 - Belen de Escobar, Provincia de Buenos Aires, Argentina
[*]smiriuka@fleni.org.ar

ABSTRACT

[[1]]

	NAME	MEM.SHIP
hsa-let-7a-5p/hsa-let-7a-1	hsa-let-7a-5p/hsa-let-7a-1	0.9999504
hsa-let-7a-3p/hsa-let-7a-1	hsa-let-7a-3p/hsa-let-7a-1	0.9825007
hsa-let-7a-5p/hsa-let-7a-2	hsa-let-7a-5p/hsa-let-7a-2	0.9999527
hsa-let-7a-5p/hsa-let-7a-3	hsa-let-7a-5p/hsa-let-7a-3	0.9999625
hsa-let-7a-3p/hsa-let-7a-3	hsa-let-7a-3p/hsa-let-7a-3	0.9812492
hsa-let-7b-5p/hsa-let-7b	hsa-let-7b-5p/hsa-let-7b	0.9982536
hsa-let-7c-5p/hsa-let-7c	hsa-let-7c-5p/hsa-let-7c	0.9982753
hsa-let-7d-5p/hsa-let-7d	hsa-let-7d-5p/hsa-let-7d	0.9826983
hsa-let-7d-3p/hsa-let-7d	hsa-let-7d-3p/hsa-let-7d	0.9961365
hsa-let-7e-5p/hsa-let-7e	hsa-let-7e-5p/hsa-let-7e	0.9992938
hsa-let-7e-3p/hsa-let-7e	hsa-let-7e-3p/hsa-let-7e	0.9999566
hsa-let-7f-5p/hsa-let-7f-1	hsa-let-7f-5p/hsa-let-7f-1	0.9855286
hsa-let-7f-5p/hsa-let-7f-2	hsa-let-7f-5p/hsa-let-7f-2	0.9856969
hsa-let-7g-5p/hsa-let-7g	hsa-let-7g-5p/hsa-let-7g	0.9893510
hsa-miR-1-3p/hsa-mir-1-1	hsa-miR-1-3p/hsa-mir-1-1	0.9999209
hsa-miR-1-3p/hsa-mir-1-2	hsa-miR-1-3p/hsa-mir-1-2	0.9999213
hsa-miR-100-5p/hsa-mir-100	hsa-miR-100-5p/hsa-mir-100	0.9980722
hsa-miR-10a-5p/hsa-mir-10a	hsa-miR-10a-5p/hsa-mir-10a	0.9210199
hsa-miR-10a-3p/hsa-mir-10a	hsa-miR-10a-3p/hsa-mir-10a	0.9016453
hsa-miR-10b-5p/hsa-mir-10b	hsa-miR-10b-5p/hsa-mir-10b	0.9986130
hsa-miR-122-5p/hsa-mir-122	hsa-miR-122-5p/hsa-mir-122	0.9360558
hsa-miR-1226-5p/hsa-mir-1226	hsa-miR-1226-5p/hsa-mir-1226	0.9909916
hsa-miR-1226-3p/hsa-mir-1226	hsa-miR-1226-3p/hsa-mir-1226	0.9995260
hsa-miR-1228-5p/hsa-mir-1228	hsa-miR-1228-5p/hsa-mir-1228	0.9993874
hsa-miR-1229-3p/hsa-mir-1229	hsa-miR-1229-3p/hsa-mir-1229	0.9959891
hsa-miR-1246/hsa-mir-1246	hsa-miR-1246/hsa-mir-1246	0.8260523
hsa-miR-125a-5p/hsa-mir-125a	hsa-miR-125a-5p/hsa-mir-125a	0.9992489
hsa-miR-125b-5p/hsa-mir-125b-1	hsa-miR-125b-5p/hsa-mir-125b-1	0.9979783
hsa-miR-125b-1-3p/hsa-mir-125b-1	hsa-miR-125b-1-3p/hsa-mir-125b-1	0.9935424
hsa-miR-125b-5p/hsa-mir-125b-2	hsa-miR-125b-5p/hsa-mir-125b-2	0.9979635
hsa-miR-125b-2-3p/hsa-mir-125b-2	hsa-miR-125b-2-3p/hsa-mir-125b-2	0.9986774
hsa-miR-1269a/hsa-mir-1269a	hsa-miR-1269a/hsa-mir-1269a	0.8561939
hsa-miR-1269b/hsa-mir-1269b	hsa-miR-1269b/hsa-mir-1269b	0.9570958
hsa-miR-1271-5p/hsa-mir-1271	hsa-miR-1271-5p/hsa-mir-1271	0.9883782
hsa-miR-1277-5p/hsa-mir-1277	hsa-miR-1277-5p/hsa-mir-1277	0.9936900
hsa-miR-1277-3p/hsa-mir-1277	hsa-miR-1277-3p/hsa-mir-1277	0.9983574
hsa-miR-129-5p/hsa-mir-129-1	hsa-miR-129-5p/hsa-mir-129-1	0.9992174
hsa-miR-129-5p/hsa-mir-129-2	hsa-miR-129-5p/hsa-mir-129-2	0.9992174
hsa-miR-129-2-3p/hsa-mir-129-2	hsa-miR-129-2-3p/hsa-mir-129-2	0.9972829
hsa-miR-1290/hsa-mir-1290	hsa-miR-1290/hsa-mir-1290	0.9110183
hsa-miR-1301-3p/hsa-mir-1301	hsa-miR-1301-3p/hsa-mir-1301	0.9548124
hsa-miR-132-5p/hsa-mir-132	hsa-miR-132-5p/hsa-mir-132	0.9951338
hsa-miR-132-3p/hsa-mir-132	hsa-miR-132-3p/hsa-mir-132	0.9844168
hsa-miR-133a-5p/hsa-mir-133a-1	hsa-miR-133a-5p/hsa-mir-133a-1	0.9941540
hsa-miR-133a-3p/hsa-mir-133a-1	hsa-miR-133a-3p/hsa-mir-133a-1	0.9946858
hsa-miR-133a-5p/hsa-mir-133a-2	hsa-miR-133a-5p/hsa-mir-133a-2	0.9941540
hsa-miR-133a-3p/hsa-mir-133a-2	hsa-miR-133a-3p/hsa-mir-133a-2	0.9946858
hsa-miR-133b/hsa-mir-133b	hsa-miR-133b/hsa-mir-133b	0.9923439
hsa-miR-134-5p/hsa-mir-134	hsa-miR-134-5p/hsa-mir-134	0.9826546
hsa-miR-140-5p/hsa-mir-140	hsa-miR-140-5p/hsa-mir-140	0.9990580
hsa-miR-143-5p/hsa-mir-143	hsa-miR-143-5p/hsa-mir-143	0.9926450
hsa-miR-143-3p/hsa-mir-143	hsa-miR-143-3p/hsa-mir-143	0.9933674
hsa-miR-145-5p/hsa-mir-145	hsa-miR-145-5p/hsa-mir-145	0.9999794
hsa-miR-145-3p/hsa-mir-145	hsa-miR-145-3p/hsa-mir-145	0.9920946
hsa-miR-148b-5p/hsa-mir-148b	hsa-miR-148b-5p/hsa-mir-148b	0.9450881
hsa-miR-148b-3p/hsa-mir-148b	hsa-miR-148b-3p/hsa-mir-148b	0.9543848
hsa-miR-151a-5p/hsa-mir-151a	hsa-miR-151a-5p/hsa-mir-151a	0.9947049
hsa-miR-151b/hsa-mir-151b	hsa-miR-151b/hsa-mir-151b	0.9999978
hsa-miR-152-5p/hsa-mir-152	hsa-miR-152-5p/hsa-mir-152	0.9981693
hsa-miR-152-3p/hsa-mir-152	hsa-miR-152-3p/hsa-mir-152	0.9985665
hsa-miR-181a-5p/hsa-mir-181a-1	hsa-miR-181a-5p/hsa-mir-181a-1	0.9984285
hsa-miR-181a-3p/hsa-mir-181a-1	hsa-miR-181a-3p/hsa-mir-181a-1	0.9999303

hsa-miR-181a-5p/hsa-mir-181a-2	hsa-miR-181a-5p/hsa-mir-181a-2	0.9984296
hsa-miR-181a-2-3p/hsa-mir-181a-2	hsa-miR-181a-2-3p/hsa-mir-181a-2	0.9937515
hsa-miR-181b-5p/hsa-mir-181b-1	hsa-miR-181b-5p/hsa-mir-181b-1	0.9999922
hsa-miR-181b-5p/hsa-mir-181b-2	hsa-miR-181b-5p/hsa-mir-181b-2	0.9999867
hsa-miR-181b-2-3p/hsa-mir-181b-2	hsa-miR-181b-2-3p/hsa-mir-181b-2	0.7693284
hsa-miR-181c-5p/hsa-mir-181c	hsa-miR-181c-5p/hsa-mir-181c	0.9946840
hsa-miR-181c-3p/hsa-mir-181c	hsa-miR-181c-3p/hsa-mir-181c	0.7905607
hsa-miR-181d-5p/hsa-mir-181d	hsa-miR-181d-5p/hsa-mir-181d	0.8652201
hsa-miR-184/hsa-mir-184	hsa-miR-184/hsa-mir-184	0.9999582
hsa-miR-186-5p/hsa-mir-186	hsa-miR-186-5p/hsa-mir-186	0.9390749
hsa-miR-190a-5p/hsa-mir-190a	hsa-miR-190a-5p/hsa-mir-190a	0.9998709
hsa-miR-1914-5p/hsa-mir-1914	hsa-miR-1914-5p/hsa-mir-1914	0.9343663
hsa-miR-192-5p/hsa-mir-192	hsa-miR-192-5p/hsa-mir-192	0.9919734
hsa-miR-193b-5p/hsa-mir-193b	hsa-miR-193b-5p/hsa-mir-193b	0.9997841
hsa-miR-194-5p/hsa-mir-194-1	hsa-miR-194-5p/hsa-mir-194-1	0.9988581
hsa-miR-194-5p/hsa-mir-194-2	hsa-miR-194-5p/hsa-mir-194-2	0.9990409
hsa-miR-196b-5p/hsa-mir-196b	hsa-miR-196b-5p/hsa-mir-196b	0.9976961
hsa-miR-199a-5p/hsa-mir-199a-1	hsa-miR-199a-5p/hsa-mir-199a-1	0.9904254
hsa-miR-199a-3p/hsa-mir-199a-1	hsa-miR-199a-3p/hsa-mir-199a-1	0.9680151
hsa-miR-199a-5p/hsa-mir-199a-2	hsa-miR-199a-5p/hsa-mir-199a-2	0.9904516
hsa-miR-199a-3p/hsa-mir-199a-2	hsa-miR-199a-3p/hsa-mir-199a-2	0.9678453
hsa-miR-199b-3p/hsa-mir-199b	hsa-miR-199b-3p/hsa-mir-199b	0.9680151
hsa-miR-206/hsa-mir-206	hsa-miR-206/hsa-mir-206	0.9925924
hsa-miR-208a-5p/hsa-mir-208a	hsa-miR-208a-5p/hsa-mir-208a	0.9997386
hsa-miR-208a-3p/hsa-mir-208a	hsa-miR-208a-3p/hsa-mir-208a	0.9992939
hsa-miR-208b-3p/hsa-mir-208b	hsa-miR-208b-3p/hsa-mir-208b	0.9999602
hsa-miR-21-5p/hsa-mir-21	hsa-miR-21-5p/hsa-mir-21	0.9757286
hsa-miR-21-3p/hsa-mir-21	hsa-miR-21-3p/hsa-mir-21	0.8793216
hsa-miR-212-5p/hsa-mir-212	hsa-miR-212-5p/hsa-mir-212	0.9901955
hsa-miR-214-5p/hsa-mir-214	hsa-miR-214-5p/hsa-mir-214	0.9802152
hsa-miR-214-3p/hsa-mir-214	hsa-miR-214-3p/hsa-mir-214	0.9854718
hsa-miR-216b-5p/hsa-mir-216b	hsa-miR-216b-5p/hsa-mir-216b	0.9998922
hsa-miR-218-5p/hsa-mir-218-1	hsa-miR-218-5p/hsa-mir-218-1	0.9983980
hsa-miR-218-5p/hsa-mir-218-2	hsa-miR-218-5p/hsa-mir-218-2	0.9983913
hsa-miR-218-2-3p/hsa-mir-218-2	hsa-miR-218-2-3p/hsa-mir-218-2	0.9998571
hsa-miR-22-5p/hsa-mir-22	hsa-miR-22-5p/hsa-mir-22	0.9902441
hsa-miR-22-3p/hsa-mir-22	hsa-miR-22-3p/hsa-mir-22	0.9980497
hsa-miR-23a-3p/hsa-mir-23a	hsa-miR-23a-3p/hsa-mir-23a	0.9983113
hsa-miR-23b-5p/hsa-mir-23b	hsa-miR-23b-5p/hsa-mir-23b	0.7798598
hsa-miR-23b-3p/hsa-mir-23b	hsa-miR-23b-3p/hsa-mir-23b	0.9980011
hsa-miR-24-1-5p/hsa-mir-24-1	hsa-miR-24-1-5p/hsa-mir-24-1	0.9980660
hsa-miR-24-3p/hsa-mir-24-1	hsa-miR-24-3p/hsa-mir-24-1	0.9866530
hsa-miR-24-3p/hsa-mir-24-2	hsa-miR-24-3p/hsa-mir-24-2	0.9865743
hsa-miR-24-2-5p/hsa-mir-24-2	hsa-miR-24-2-5p/hsa-mir-24-2	0.9984300
hsa-miR-26b-5p/hsa-mir-26b	hsa-miR-26b-5p/hsa-mir-26b	0.9996652
hsa-miR-27a-3p/hsa-mir-27a	hsa-miR-27a-3p/hsa-mir-27a	0.9971615
hsa-miR-27b-3p/hsa-mir-27b	hsa-miR-27b-3p/hsa-mir-27b	0.9649021
hsa-miR-28-5p/hsa-mir-28	hsa-miR-28-5p/hsa-mir-28	0.9999299
hsa-miR-299-3p/hsa-mir-299	hsa-miR-299-3p/hsa-mir-299	0.9593262
hsa-miR-29b-3p/hsa-mir-29b-1	hsa-miR-29b-3p/hsa-mir-29b-1	0.7666241
hsa-miR-29b-3p/hsa-mir-29b-2	hsa-miR-29b-3p/hsa-mir-29b-2	0.7666241
hsa-miR-301a-3p/hsa-mir-301a	hsa-miR-301a-3p/hsa-mir-301a	0.8021942
hsa-miR-30a-5p/hsa-mir-30a	hsa-miR-30a-5p/hsa-mir-30a	0.9933927
hsa-miR-30a-3p/hsa-mir-30a	hsa-miR-30a-3p/hsa-mir-30a	0.7620991
hsa-miR-30b-5p/hsa-mir-30b	hsa-miR-30b-5p/hsa-mir-30b	0.9999795
hsa-miR-30b-3p/hsa-mir-30b	hsa-miR-30b-3p/hsa-mir-30b	0.9972608
hsa-miR-30c-5p/hsa-mir-30c-1	hsa-miR-30c-5p/hsa-mir-30c-1	0.9993108
hsa-miR-30c-1-3p/hsa-mir-30c-1	hsa-miR-30c-1-3p/hsa-mir-30c-1	0.9988592
hsa-miR-30c-5p/hsa-mir-30c-2	hsa-miR-30c-5p/hsa-mir-30c-2	0.9993228
hsa-miR-30d-5p/hsa-mir-30d	hsa-miR-30d-5p/hsa-mir-30d	0.8977645
hsa-miR-30d-3p/hsa-mir-30d	hsa-miR-30d-3p/hsa-mir-30d	0.9394137
hsa-miR-30e-5p/hsa-mir-30e	hsa-miR-30e-5p/hsa-mir-30e	0.9984503
hsa-miR-30e-3p/hsa-mir-30e	hsa-miR-30e-3p/hsa-mir-30e	0.9908981
hsa-miR-3200-3p/hsa-mir-3200	hsa-miR-3200-3p/hsa-mir-3200	0.9668929

hsa-miR-324-5p/hsa-mir-324	hsa-miR-324-5p/hsa-mir-324	0.9990771
hsa-miR-328-3p/hsa-mir-328	hsa-miR-328-3p/hsa-mir-328	0.9717812
hsa-miR-331-5p/hsa-mir-331	hsa-miR-331-5p/hsa-mir-331	0.9603835
hsa-miR-331-3p/hsa-mir-331	hsa-miR-331-3p/hsa-mir-331	0.9502842
hsa-miR-335-5p/hsa-mir-335	hsa-miR-335-5p/hsa-mir-335	0.8994658
hsa-miR-338-5p/hsa-mir-338	hsa-miR-338-5p/hsa-mir-338	0.9998207
hsa-miR-338-3p/hsa-mir-338	hsa-miR-338-3p/hsa-mir-338	0.9954096
hsa-miR-346/hsa-mir-346	hsa-miR-346/hsa-mir-346	0.9993994
hsa-miR-34a-5p/hsa-mir-34a	hsa-miR-34a-5p/hsa-mir-34a	0.7767643
hsa-miR-3591-5p/hsa-mir-3591	hsa-miR-3591-5p/hsa-mir-3591	0.9992665
hsa-miR-3607-5p/hsa-mir-3607	hsa-miR-3607-5p/hsa-mir-3607	0.8076347
hsa-miR-3607-3p/hsa-mir-3607	hsa-miR-3607-3p/hsa-mir-3607	0.9999999
hsa-miR-3609/hsa-mir-3609	hsa-miR-3609/hsa-mir-3609	0.9951440
hsa-miR-361-5p/hsa-mir-361	hsa-miR-361-5p/hsa-mir-361	0.9168785
hsa-miR-362-5p/hsa-mir-362	hsa-miR-362-5p/hsa-mir-362	0.8731926
hsa-miR-3653-5p/hsa-mir-3653	hsa-miR-3653-5p/hsa-mir-3653	0.9664676
hsa-miR-3653-3p/hsa-mir-3653	hsa-miR-3653-3p/hsa-mir-3653	0.9987977
hsa-miR-374a-5p/hsa-mir-374a	hsa-miR-374a-5p/hsa-mir-374a	0.9897203
hsa-miR-378a-5p/hsa-mir-378a	hsa-miR-378a-5p/hsa-mir-378a	0.9975819
hsa-miR-378a-3p/hsa-mir-378a	hsa-miR-378a-3p/hsa-mir-378a	0.9925168
hsa-miR-378b/hsa-mir-378b	hsa-miR-378b/hsa-mir-378b	0.9940784
hsa-miR-378c/hsa-mir-378c	hsa-miR-378c/hsa-mir-378c	0.9870931
hsa-miR-378d/hsa-mir-378d-1	hsa-miR-378d/hsa-mir-378d-1	0.9939011
hsa-miR-378d/hsa-mir-378d-2	hsa-miR-378d/hsa-mir-378d-2	0.9946275
hsa-miR-378f/hsa-mir-378f	hsa-miR-378f/hsa-mir-378f	0.9971287
hsa-miR-378g/hsa-mir-378g	hsa-miR-378g/hsa-mir-378g	0.9924512
hsa-miR-378i/hsa-mir-378i	hsa-miR-378i/hsa-mir-378i	0.9942946
hsa-miR-383-5p/hsa-mir-383	hsa-miR-383-5p/hsa-mir-383	0.9995056
hsa-miR-3912-3p/hsa-mir-3912	hsa-miR-3912-3p/hsa-mir-3912	0.9478309
hsa-miR-3938/hsa-mir-3938	hsa-miR-3938/hsa-mir-3938	0.9990292
hsa-miR-410-3p/hsa-mir-410	hsa-miR-410-3p/hsa-mir-410	0.9916150
hsa-miR-411-5p/hsa-mir-411	hsa-miR-411-5p/hsa-mir-411	0.7561420
hsa-miR-411-3p/hsa-mir-411	hsa-miR-411-3p/hsa-mir-411	0.9712818
hsa-miR-422a/hsa-mir-422a	hsa-miR-422a/hsa-mir-422a	0.9950909
hsa-miR-424-5p/hsa-mir-424	hsa-miR-424-5p/hsa-mir-424	0.9859566
hsa-miR-424-3p/hsa-mir-424	hsa-miR-424-3p/hsa-mir-424	0.8999363
hsa-miR-4301/hsa-mir-4301	hsa-miR-4301/hsa-mir-4301	0.9938973
hsa-miR-433-3p/hsa-mir-433	hsa-miR-433-3p/hsa-mir-433	0.8402681
hsa-miR-4473/hsa-mir-4473	hsa-miR-4473/hsa-mir-4473	0.9750258
hsa-miR-4485-3p/hsa-mir-4485	hsa-miR-4485-3p/hsa-mir-4485	0.9968247
hsa-miR-450a-5p/hsa-mir-450a-1	hsa-miR-450a-5p/hsa-mir-450a-1	0.9983070
hsa-miR-450a-5p/hsa-mir-450a-2	hsa-miR-450a-5p/hsa-mir-450a-2	0.9983070
hsa-miR-450a-2-3p/hsa-mir-450a-2	hsa-miR-450a-2-3p/hsa-mir-450a-2	0.9964856
hsa-miR-450b-5p/hsa-mir-450b	hsa-miR-450b-5p/hsa-mir-450b	0.9989068
hsa-miR-4532/hsa-mir-4532	hsa-miR-4532/hsa-mir-4532	0.9970331
hsa-miR-4662a-5p/hsa-mir-4662a	hsa-miR-4662a-5p/hsa-mir-4662a	0.9946371
hsa-miR-483-5p/hsa-mir-483	hsa-miR-483-5p/hsa-mir-483	0.9212795
hsa-miR-483-3p/hsa-mir-483	hsa-miR-483-3p/hsa-mir-483	0.8981758
hsa-miR-490-5p/hsa-mir-490	hsa-miR-490-5p/hsa-mir-490	0.9999556
hsa-miR-490-3p/hsa-mir-490	hsa-miR-490-3p/hsa-mir-490	0.9972247
hsa-miR-491-5p/hsa-mir-491	hsa-miR-491-5p/hsa-mir-491	0.9982874
hsa-miR-499a-5p/hsa-mir-499a	hsa-miR-499a-5p/hsa-mir-499a	0.9940497
hsa-miR-499a-3p/hsa-mir-499a	hsa-miR-499a-3p/hsa-mir-499a	0.9989261
hsa-miR-500a-3p/hsa-mir-500a	hsa-miR-500a-3p/hsa-mir-500a	0.9849089
hsa-miR-500b-5p/hsa-mir-500b	hsa-miR-500b-5p/hsa-mir-500b	0.8512438
hsa-miR-501-5p/hsa-mir-501	hsa-miR-501-5p/hsa-mir-501	0.9489421
hsa-miR-501-3p/hsa-mir-501	hsa-miR-501-3p/hsa-mir-501	0.9835684
hsa-miR-502-3p/hsa-mir-502	hsa-miR-502-3p/hsa-mir-502	0.9955486
hsa-miR-504-5p/hsa-mir-504	hsa-miR-504-5p/hsa-mir-504	0.9875857
hsa-miR-504-3p/hsa-mir-504	hsa-miR-504-3p/hsa-mir-504	0.9946332
hsa-miR-505-5p/hsa-mir-505	hsa-miR-505-5p/hsa-mir-505	0.9721123
hsa-miR-506-3p/hsa-mir-506	hsa-miR-506-3p/hsa-mir-506	0.9839919
hsa-miR-509-5p/hsa-mir-509-1	hsa-miR-509-5p/hsa-mir-509-1	0.9851144
hsa-miR-509-3p/hsa-mir-509-1	hsa-miR-509-3p/hsa-mir-509-1	0.8674008

hsa-miR-509-5p/hsa-mir-509-2	hsa-miR-509-5p/hsa-mir-509-2	0.9851144
hsa-miR-509-3p/hsa-mir-509-2	hsa-miR-509-3p/hsa-mir-509-2	0.8674008
hsa-miR-509-3p/hsa-mir-509-3	hsa-miR-509-3p/hsa-mir-509-3	0.8674008
hsa-miR-509-3-5p/hsa-mir-509-3	hsa-miR-509-3-5p/hsa-mir-509-3	0.9183285
hsa-miR-5096/hsa-mir-5096	hsa-miR-5096/hsa-mir-5096	0.9900542
hsa-miR-513c-5p/hsa-mir-513c	hsa-miR-513c-5p/hsa-mir-513c	0.9767150
hsa-miR-514a-3p/hsa-mir-514a-1	hsa-miR-514a-3p/hsa-mir-514a-1	0.9835345
hsa-miR-514a-3p/hsa-mir-514a-2	hsa-miR-514a-3p/hsa-mir-514a-2	0.9835345
hsa-miR-514a-3p/hsa-mir-514a-3	hsa-miR-514a-3p/hsa-mir-514a-3	0.9835345
hsa-miR-542-3p/hsa-mir-542	hsa-miR-542-3p/hsa-mir-542	0.9999354
hsa-miR-548ab/hsa-mir-548ab	hsa-miR-548ab/hsa-mir-548ab	0.9289213
hsa-miR-548e-3p/hsa-mir-548e	hsa-miR-548e-3p/hsa-mir-548e	0.9906273
hsa-miR-550a-3-5p/hsa-mir-550a-3	hsa-miR-550a-3-5p/hsa-mir-550a-3	0.9543915
hsa-miR-551b-5p/hsa-mir-551b	hsa-miR-551b-5p/hsa-mir-551b	0.9978948
hsa-miR-551b-3p/hsa-mir-551b	hsa-miR-551b-3p/hsa-mir-551b	0.9950501
hsa-miR-574-3p/hsa-mir-574	hsa-miR-574-3p/hsa-mir-574	0.8396930
hsa-miR-584-5p/hsa-mir-584	hsa-miR-584-5p/hsa-mir-584	0.9824702
hsa-miR-585-3p/hsa-mir-585	hsa-miR-585-3p/hsa-mir-585	0.9967471
hsa-miR-598-5p/hsa-mir-598	hsa-miR-598-5p/hsa-mir-598	0.9957933
hsa-miR-598-3p/hsa-mir-598	hsa-miR-598-3p/hsa-mir-598	0.9995846
hsa-miR-615-3p/hsa-mir-615	hsa-miR-615-3p/hsa-mir-615	0.9881813
hsa-miR-619-5p/hsa-mir-619	hsa-miR-619-5p/hsa-mir-619	0.9933302
hsa-miR-628-5p/hsa-mir-628	hsa-miR-628-5p/hsa-mir-628	0.9944346
hsa-miR-628-3p/hsa-mir-628	hsa-miR-628-3p/hsa-mir-628	0.9284520
hsa-miR-6505-5p/hsa-mir-6505	hsa-miR-6505-5p/hsa-mir-6505	0.9975126
hsa-miR-6505-3p/hsa-mir-6505	hsa-miR-6505-3p/hsa-mir-6505	0.9998677
hsa-miR-6516-5p/hsa-mir-6516	hsa-miR-6516-5p/hsa-mir-6516	0.9989414
hsa-miR-660-5p/hsa-mir-660	hsa-miR-660-5p/hsa-mir-660	0.9971647
hsa-miR-664a-5p/hsa-mir-664a	hsa-miR-664a-5p/hsa-mir-664a	0.9980778
hsa-miR-670-3p/hsa-mir-670	hsa-miR-670-3p/hsa-mir-670	0.9997070
hsa-miR-675-5p/hsa-mir-675	hsa-miR-675-5p/hsa-mir-675	0.9522535
hsa-miR-675-3p/hsa-mir-675	hsa-miR-675-3p/hsa-mir-675	0.9369275
hsa-miR-7151-5p/hsa-mir-7151	hsa-miR-7151-5p/hsa-mir-7151	0.9957197
hsa-miR-7151-3p/hsa-mir-7151	hsa-miR-7151-3p/hsa-mir-7151	0.9937862
hsa-miR-7704/hsa-mir-7704	hsa-miR-7704/hsa-mir-7704	0.9991466
hsa-miR-874-3p/hsa-mir-874	hsa-miR-874-3p/hsa-mir-874	0.9998862
hsa-miR-876-3p/hsa-mir-876	hsa-miR-876-3p/hsa-mir-876	0.9999995
hsa-miR-887-5p/hsa-mir-887	hsa-miR-887-5p/hsa-mir-887	0.9271585
hsa-miR-887-3p/hsa-mir-887	hsa-miR-887-3p/hsa-mir-887	0.9981109
hsa-miR-888-5p/hsa-mir-888	hsa-miR-888-5p/hsa-mir-888	0.9971154
hsa-miR-95-3p/hsa-mir-95	hsa-miR-95-3p/hsa-mir-95	0.9906650
hsa-miR-98-5p/hsa-mir-98	hsa-miR-98-5p/hsa-mir-98	0.9606225
hsa-miR-99a-5p/hsa-mir-99a	hsa-miR-99a-5p/hsa-mir-99a	0.9967850
hsa-miR-99a-3p/hsa-mir-99a	hsa-miR-99a-3p/hsa-mir-99a	0.9983390

[[2]]

	NAME	MEM.SHIP
hsa-miR-106a-5p/hsa-mir-106a	hsa-miR-106a-5p/hsa-mir-106a	0.9952571
hsa-miR-106a-3p/hsa-mir-106a	hsa-miR-106a-3p/hsa-mir-106a	0.8344156
hsa-miR-106b-5p/hsa-mir-106b	hsa-miR-106b-5p/hsa-mir-106b	0.9985628
hsa-miR-124-3p/hsa-mir-124-1	hsa-miR-124-3p/hsa-mir-124-1	0.9052711
hsa-miR-124-3p/hsa-mir-124-2	hsa-miR-124-3p/hsa-mir-124-2	0.9164731
hsa-miR-124-3p/hsa-mir-124-3	hsa-miR-124-3p/hsa-mir-124-3	0.9164731
hsa-miR-1266-5p/hsa-mir-1266	hsa-miR-1266-5p/hsa-mir-1266	0.8523660
hsa-miR-1278/hsa-mir-1278	hsa-miR-1278/hsa-mir-1278	0.9810142
hsa-miR-1285-3p/hsa-mir-1285-1	hsa-miR-1285-3p/hsa-mir-1285-1	0.8841762
hsa-miR-1285-3p/hsa-mir-1285-2	hsa-miR-1285-3p/hsa-mir-1285-2	0.8986315
hsa-miR-1304-5p/hsa-mir-1304	hsa-miR-1304-5p/hsa-mir-1304	0.8788830
hsa-miR-1304-3p/hsa-mir-1304	hsa-miR-1304-3p/hsa-mir-1304	0.9950131
hsa-miR-135b-5p/hsa-mir-135b	hsa-miR-135b-5p/hsa-mir-135b	0.9999422
hsa-miR-135b-3p/hsa-mir-135b	hsa-miR-135b-3p/hsa-mir-135b	0.9966686
hsa-miR-138-5p/hsa-mir-138-1	hsa-miR-138-5p/hsa-mir-138-1	0.9893615
hsa-miR-138-5p/hsa-mir-138-2	hsa-miR-138-5p/hsa-mir-138-2	0.9004179
hsa-miR-148a-5p/hsa-mir-148a	hsa-miR-148a-5p/hsa-mir-148a	0.9477915

```
hsa-miR-155-5p/hsa-mir-155              hsa-miR-155-5p/hsa-mir-155 0.9996695
hsa-miR-15b-5p/hsa-mir-15b              hsa-miR-15b-5p/hsa-mir-15b 0.9449166
hsa-miR-15b-3p/hsa-mir-15b              hsa-miR-15b-3p/hsa-mir-15b 0.9976242
hsa-miR-16-5p/hsa-mir-16-1              hsa-miR-16-5p/hsa-mir-16-1 0.9299175
hsa-miR-16-5p/hsa-mir-16-2              hsa-miR-16-5p/hsa-mir-16-2 0.9342315
hsa-miR-16-2-3p/hsa-mir-16-2          hsa-miR-16-2-3p/hsa-mir-16-2 0.9396065
hsa-miR-17-5p/hsa-mir-17                  hsa-miR-17-5p/hsa-mir-17 0.9916215
hsa-miR-17-3p/hsa-mir-17                  hsa-miR-17-3p/hsa-mir-17 0.9997987
hsa-miR-187-3p/hsa-mir-187              hsa-miR-187-3p/hsa-mir-187 0.9878737
hsa-miR-18a-5p/hsa-mir-18a              hsa-miR-18a-5p/hsa-mir-18a 0.9638298
hsa-miR-18b-5p/hsa-mir-18b              hsa-miR-18b-5p/hsa-mir-18b 0.9957594
hsa-miR-20a-5p/hsa-mir-20a              hsa-miR-20a-5p/hsa-mir-20a 0.9974143
hsa-miR-20a-3p/hsa-mir-20a              hsa-miR-20a-3p/hsa-mir-20a 0.9867819
hsa-miR-20b-5p/hsa-mir-20b              hsa-miR-20b-5p/hsa-mir-20b 0.9884360
hsa-miR-20b-3p/hsa-mir-20b              hsa-miR-20b-3p/hsa-mir-20b 0.9851180
hsa-miR-219a-2-3p/hsa-mir-219a-2 hsa-miR-219a-2-3p/hsa-mir-219a-2 0.8335419
hsa-miR-221-3p/hsa-mir-221              hsa-miR-221-3p/hsa-mir-221 0.9860045
hsa-miR-222-5p/hsa-mir-222              hsa-miR-222-5p/hsa-mir-222 0.8958867
hsa-miR-222-3p/hsa-mir-222              hsa-miR-222-3p/hsa-mir-222 0.9392875
hsa-miR-301b-5p/hsa-mir-301b          hsa-miR-301b-5p/hsa-mir-301b 0.7900298
hsa-miR-302a-5p/hsa-mir-302a          hsa-miR-302a-5p/hsa-mir-302a 0.9814319
hsa-miR-302a-3p/hsa-mir-302a          hsa-miR-302a-3p/hsa-mir-302a 0.9583738
hsa-miR-302b-5p/hsa-mir-302b          hsa-miR-302b-5p/hsa-mir-302b 0.8719594
hsa-miR-302b-3p/hsa-mir-302b          hsa-miR-302b-3p/hsa-mir-302b 0.8427805
hsa-miR-302c-5p/hsa-mir-302c          hsa-miR-302c-5p/hsa-mir-302c 0.9996172
hsa-miR-302c-3p/hsa-mir-302c          hsa-miR-302c-3p/hsa-mir-302c 0.8196741
hsa-miR-302d-5p/hsa-mir-302d          hsa-miR-302d-5p/hsa-mir-302d 0.9972956
hsa-miR-302d-3p/hsa-mir-302d          hsa-miR-302d-3p/hsa-mir-302d 0.9852902
hsa-miR-302e/hsa-mir-302e                hsa-miR-302e/hsa-mir-302e 0.9672854
hsa-miR-3144-3p/hsa-mir-3144          hsa-miR-3144-3p/hsa-mir-3144 0.9998880
hsa-miR-335-3p/hsa-mir-335              hsa-miR-335-3p/hsa-mir-335 0.9678290
hsa-miR-363-3p/hsa-mir-363              hsa-miR-363-3p/hsa-mir-363 0.9945926
hsa-miR-3662/hsa-mir-3662                hsa-miR-3662/hsa-mir-3662 0.9737736
hsa-miR-367-3p/hsa-mir-367              hsa-miR-367-3p/hsa-mir-367 0.9954327
hsa-miR-371a-5p/hsa-mir-371a          hsa-miR-371a-5p/hsa-mir-371a 0.9975460
hsa-miR-373-5p/hsa-mir-373              hsa-miR-373-5p/hsa-mir-373 0.9998690
hsa-miR-373-3p/hsa-mir-373              hsa-miR-373-3p/hsa-mir-373 0.7947026
hsa-miR-3934-5p/hsa-mir-3934          hsa-miR-3934-5p/hsa-mir-3934 0.9368584
hsa-miR-3937/hsa-mir-3937                hsa-miR-3937/hsa-mir-3937 0.9679612
hsa-miR-3939/hsa-mir-3939                hsa-miR-3939/hsa-mir-3939 0.9392306
hsa-miR-4521/hsa-mir-4521                hsa-miR-4521/hsa-mir-4521 0.9994503
hsa-miR-548aq-3p/hsa-mir-548aq      hsa-miR-548aq-3p/hsa-mir-548aq 0.9101887
hsa-miR-548k/hsa-mir-548k                hsa-miR-548k/hsa-mir-548k 0.9968194
hsa-miR-548o-3p/hsa-mir-548o          hsa-miR-548o-3p/hsa-mir-548o 0.9980726
hsa-miR-548o-3p/hsa-mir-548o-2      hsa-miR-548o-3p/hsa-mir-548o-2 0.9980726
hsa-miR-548u/hsa-mir-548u                hsa-miR-548u/hsa-mir-548u 0.9252426
hsa-miR-589-5p/hsa-mir-589              hsa-miR-589-5p/hsa-mir-589 0.9981122
hsa-miR-592/hsa-mir-592                    hsa-miR-592/hsa-mir-592 0.8795448
hsa-miR-618/hsa-mir-618                    hsa-miR-618/hsa-mir-618 0.7613506
hsa-miR-641/hsa-mir-641                    hsa-miR-641/hsa-mir-641 0.9942372
hsa-miR-651-5p/hsa-mir-651              hsa-miR-651-5p/hsa-mir-651 0.9994022
hsa-miR-7-5p/hsa-mir-7-1                  hsa-miR-7-5p/hsa-mir-7-1 0.9998419
hsa-miR-7-5p/hsa-mir-7-2                  hsa-miR-7-5p/hsa-mir-7-2 0.9998929
hsa-miR-7-5p/hsa-mir-7-3                  hsa-miR-7-5p/hsa-mir-7-3 0.9998964
hsa-miR-873-5p/hsa-mir-873              hsa-miR-873-5p/hsa-mir-873 0.7529556
hsa-miR-873-3p/hsa-mir-873              hsa-miR-873-3p/hsa-mir-873 0.8356278
hsa-miR-92b-5p/hsa-mir-92b              hsa-miR-92b-5p/hsa-mir-92b 0.9998848
hsa-miR-93-5p/hsa-mir-93                  hsa-miR-93-5p/hsa-mir-93 0.9762327
hsa-miR-942-5p/hsa-mir-942              hsa-miR-942-5p/hsa-mir-942 0.9970676

[[3]]
                                                              NAME  MEM.SHIP
hsa-miR-105-5p/hsa-mir-105-1          hsa-miR-105-5p/hsa-mir-105-1 0.9954713
hsa-miR-105-5p/hsa-mir-105-2          hsa-miR-105-5p/hsa-mir-105-2 0.9954713
```

hsa-miR-106b-3p/hsa-mir-106b	hsa-miR-106b-3p/hsa-mir-106b	0.7815504
hsa-miR-1243/hsa-mir-1243	hsa-miR-1243/hsa-mir-1243	0.9927474
hsa-miR-1247-3p/hsa-mir-1247	hsa-miR-1247-3p/hsa-mir-1247	0.9992493
hsa-miR-1254/hsa-mir-1254-1	hsa-miR-1254/hsa-mir-1254-1	0.9203939
hsa-miR-1254/hsa-mir-1254-2	hsa-miR-1254/hsa-mir-1254-2	0.9187724
hsa-miR-1255a/hsa-mir-1255a	hsa-miR-1255a/hsa-mir-1255a	0.9999490
hsa-miR-1263/hsa-mir-1263	hsa-miR-1263/hsa-mir-1263	0.9998467
hsa-miR-1270/hsa-mir-1270	hsa-miR-1270/hsa-mir-1270	0.8715520
hsa-miR-1275/hsa-mir-1275	hsa-miR-1275/hsa-mir-1275	0.9997560
hsa-miR-128-1-5p/hsa-mir-128-1	hsa-miR-128-1-5p/hsa-mir-128-1	0.8542037
hsa-miR-1292-5p/hsa-mir-1292	hsa-miR-1292-5p/hsa-mir-1292	0.9995237
hsa-miR-1294/hsa-mir-1294	hsa-miR-1294/hsa-mir-1294	0.9783041
hsa-miR-1306-3p/hsa-mir-1306	hsa-miR-1306-3p/hsa-mir-1306	0.9986577
hsa-miR-130b-5p/hsa-mir-130b	hsa-miR-130b-5p/hsa-mir-130b	0.9996407
hsa-miR-135a-3p/hsa-mir-135a-1	hsa-miR-135a-3p/hsa-mir-135a-1	0.9662552
hsa-miR-139-5p/hsa-mir-139	hsa-miR-139-5p/hsa-mir-139	0.9887601
hsa-miR-139-3p/hsa-mir-139	hsa-miR-139-3p/hsa-mir-139	0.9964222
hsa-miR-1468-5p/hsa-mir-1468	hsa-miR-1468-5p/hsa-mir-1468	0.9964007
hsa-miR-151a-3p/hsa-mir-151a	hsa-miR-151a-3p/hsa-mir-151a	0.9758510
hsa-miR-15a-5p/hsa-mir-15a	hsa-miR-15a-5p/hsa-mir-15a	0.9997654
hsa-miR-191-5p/hsa-mir-191	hsa-miR-191-5p/hsa-mir-191	0.9437344
hsa-miR-191-3p/hsa-mir-191	hsa-miR-191-3p/hsa-mir-191	0.9815634
hsa-miR-1910-5p/hsa-mir-1910	hsa-miR-1910-5p/hsa-mir-1910	0.8136871
hsa-miR-195-5p/hsa-mir-195	hsa-miR-195-5p/hsa-mir-195	0.9899006
hsa-miR-195-3p/hsa-mir-195	hsa-miR-195-3p/hsa-mir-195	0.9819416
hsa-miR-203a-3p/hsa-mir-203a	hsa-miR-203a-3p/hsa-mir-203a	0.9058510
hsa-miR-204-5p/hsa-mir-204	hsa-miR-204-5p/hsa-mir-204	0.9999999
hsa-miR-219a-1-3p/hsa-mir-219a-1	hsa-miR-219a-1-3p/hsa-mir-219a-1	0.8662587
hsa-miR-224-5p/hsa-mir-224	hsa-miR-224-5p/hsa-mir-224	0.8895123
hsa-miR-2277-5p/hsa-mir-2277	hsa-miR-2277-5p/hsa-mir-2277	0.9949854
hsa-miR-25-5p/hsa-mir-25	hsa-miR-25-5p/hsa-mir-25	0.9803098
hsa-miR-25-3p/hsa-mir-25	hsa-miR-25-3p/hsa-mir-25	0.8384923
hsa-miR-27a-5p/hsa-mir-27a	hsa-miR-27a-5p/hsa-mir-27a	0.9972418
hsa-miR-296-5p/hsa-mir-296	hsa-miR-296-5p/hsa-mir-296	0.8109991
hsa-miR-301a-5p/hsa-mir-301a	hsa-miR-301a-5p/hsa-mir-301a	0.9602063
hsa-miR-3074-5p/hsa-mir-3074	hsa-miR-3074-5p/hsa-mir-3074	0.9936698
hsa-miR-3177-3p/hsa-mir-3177	hsa-miR-3177-3p/hsa-mir-3177	0.9999405
hsa-miR-3187-3p/hsa-mir-3187	hsa-miR-3187-3p/hsa-mir-3187	0.9898520
hsa-miR-326/hsa-mir-326	hsa-miR-326/hsa-mir-326	0.8487862
hsa-miR-330-5p/hsa-mir-330	hsa-miR-330-5p/hsa-mir-330	0.9998017
hsa-miR-330-3p/hsa-mir-330	hsa-miR-330-3p/hsa-mir-330	0.9544717
hsa-miR-363-5p/hsa-mir-363	hsa-miR-363-5p/hsa-mir-363	0.9811125
hsa-miR-365b-5p/hsa-mir-365b	hsa-miR-365b-5p/hsa-mir-365b	0.9988875
hsa-miR-3661/hsa-mir-3661	hsa-miR-3661/hsa-mir-3661	0.9750084
hsa-miR-3691-5p/hsa-mir-3691	hsa-miR-3691-5p/hsa-mir-3691	0.9356976
hsa-miR-370-3p/hsa-mir-370	hsa-miR-370-3p/hsa-mir-370	0.9352851
hsa-miR-375/hsa-mir-375	hsa-miR-375/hsa-mir-375	0.9979136
hsa-miR-3907/hsa-mir-3907	hsa-miR-3907/hsa-mir-3907	0.9958933
hsa-miR-3928-3p/hsa-mir-3928	hsa-miR-3928-3p/hsa-mir-3928	0.8711210
hsa-miR-3940-3p/hsa-mir-3940	hsa-miR-3940-3p/hsa-mir-3940	0.9992711
hsa-miR-423-3p/hsa-mir-423	hsa-miR-423-3p/hsa-mir-423	0.9428655
hsa-miR-425-3p/hsa-mir-425	hsa-miR-425-3p/hsa-mir-425	0.9520151
hsa-miR-4326/hsa-mir-4326	hsa-miR-4326/hsa-mir-4326	0.9999255
hsa-miR-454-5p/hsa-mir-454	hsa-miR-454-5p/hsa-mir-454	0.9984501
hsa-miR-454-3p/hsa-mir-454	hsa-miR-454-3p/hsa-mir-454	0.9799187
hsa-miR-4665-5p/hsa-mir-4665	hsa-miR-4665-5p/hsa-mir-4665	0.9866227
hsa-miR-4707-5p/hsa-mir-4707	hsa-miR-4707-5p/hsa-mir-4707	0.9972216
hsa-miR-4741/hsa-mir-4741	hsa-miR-4741/hsa-mir-4741	0.9967946
hsa-miR-489-3p/hsa-mir-489	hsa-miR-489-3p/hsa-mir-489	0.9766416
hsa-miR-493-5p/hsa-mir-493	hsa-miR-493-5p/hsa-mir-493	0.9870351
hsa-miR-497-5p/hsa-mir-497	hsa-miR-497-5p/hsa-mir-497	0.9981270
hsa-miR-505-3p/hsa-mir-505	hsa-miR-505-3p/hsa-mir-505	0.8550113
hsa-miR-5100/hsa-mir-5100	hsa-miR-5100/hsa-mir-5100	0.9970248
hsa-miR-576-3p/hsa-mir-576	hsa-miR-576-3p/hsa-mir-576	0.9959343

```
hsa-miR-582-3p/hsa-mir-582             hsa-miR-582-3p/hsa-mir-582 0.9995644
hsa-miR-597-3p/hsa-mir-597             hsa-miR-597-3p/hsa-mir-597 0.9876876
hsa-miR-625-3p/hsa-mir-625             hsa-miR-625-3p/hsa-mir-625 0.9739104
hsa-miR-629-5p/hsa-mir-629             hsa-miR-629-5p/hsa-mir-629 0.9953671
hsa-miR-629-3p/hsa-mir-629             hsa-miR-629-3p/hsa-mir-629 0.9802861
hsa-miR-653-5p/hsa-mir-653             hsa-miR-653-5p/hsa-mir-653 0.9924738
hsa-miR-653-3p/hsa-mir-653             hsa-miR-653-3p/hsa-mir-653 0.9780757
hsa-miR-6783-3p/hsa-mir-6783         hsa-miR-6783-3p/hsa-mir-6783 0.9890980
hsa-miR-6842-3p/hsa-mir-6842         hsa-miR-6842-3p/hsa-mir-6842 0.9922759
hsa-miR-708-5p/hsa-mir-708             hsa-miR-708-5p/hsa-mir-708 0.9945612
hsa-miR-708-3p/hsa-mir-708             hsa-miR-708-3p/hsa-mir-708 0.9963956
hsa-miR-7641/hsa-mir-7641-1           hsa-miR-7641/hsa-mir-7641-1 0.9147837
hsa-miR-7641/hsa-mir-7641-2           hsa-miR-7641/hsa-mir-7641-2 0.9147837
hsa-miR-767-5p/hsa-mir-767             hsa-miR-767-5p/hsa-mir-767 0.9572567
hsa-miR-769-5p/hsa-mir-769             hsa-miR-769-5p/hsa-mir-769 0.9944529
hsa-miR-7706/hsa-mir-7706               hsa-miR-7706/hsa-mir-7706 0.8146557
hsa-miR-7977/hsa-mir-7977               hsa-miR-7977/hsa-mir-7977 0.9848125
hsa-miR-877-3p/hsa-mir-877             hsa-miR-877-3p/hsa-mir-877 0.8502057
hsa-miR-9-5p/hsa-mir-9-1                 hsa-miR-9-5p/hsa-mir-9-1 0.9810371
hsa-miR-9-3p/hsa-mir-9-1                 hsa-miR-9-3p/hsa-mir-9-1 0.9796678
hsa-miR-9-5p/hsa-mir-9-2                 hsa-miR-9-5p/hsa-mir-9-2 0.9810466
hsa-miR-9-3p/hsa-mir-9-2                 hsa-miR-9-3p/hsa-mir-9-2 0.9796678
hsa-miR-9-5p/hsa-mir-9-3                 hsa-miR-9-5p/hsa-mir-9-3 0.9810408
hsa-miR-9-3p/hsa-mir-9-3                 hsa-miR-9-3p/hsa-mir-9-3 0.9796674
hsa-miR-92a-3p/hsa-mir-92a-1         hsa-miR-92a-3p/hsa-mir-92a-1 0.9610949
hsa-miR-92a-3p/hsa-mir-92a-2         hsa-miR-92a-3p/hsa-mir-92a-2 0.9599375
hsa-miR-92a-2-5p/hsa-mir-92a-2     hsa-miR-92a-2-5p/hsa-mir-92a-2 0.9572441
hsa-miR-92b-3p/hsa-mir-92b             hsa-miR-92b-3p/hsa-mir-92b 0.8023038
hsa-miR-93-3p/hsa-mir-93                 hsa-miR-93-3p/hsa-mir-93 0.9941327
hsa-miR-935/hsa-mir-935                   hsa-miR-935/hsa-mir-935 0.9993773
hsa-miR-941/hsa-mir-941-1               hsa-miR-941/hsa-mir-941-1 0.9179860
hsa-miR-941/hsa-mir-941-2               hsa-miR-941/hsa-mir-941-2 0.9179860
hsa-miR-941/hsa-mir-941-3               hsa-miR-941/hsa-mir-941-3 0.9179860
hsa-miR-941/hsa-mir-941-4               hsa-miR-941/hsa-mir-941-4 0.9179860
hsa-miR-941/hsa-mir-941-5               hsa-miR-941/hsa-mir-941-5 0.9179860
hsa-miR-99b-3p/hsa-mir-99b             hsa-miR-99b-3p/hsa-mir-99b 0.9366968

[[4]]
                                                             NAME  MEM.SHIP
hsa-miR-124-5p/hsa-mir-124-1     hsa-miR-124-5p/hsa-mir-124-1 0.9997054
hsa-miR-124-5p/hsa-mir-124-2     hsa-miR-124-5p/hsa-mir-124-2 0.9997054
hsa-miR-124-5p/hsa-mir-124-3     hsa-miR-124-5p/hsa-mir-124-3 0.9997054
hsa-miR-1251-5p/hsa-mir-1251     hsa-miR-1251-5p/hsa-mir-1251 0.9898011
hsa-miR-1264/hsa-mir-1264           hsa-miR-1264/hsa-mir-1264 0.9997578
hsa-miR-1283/hsa-mir-1283-1       hsa-miR-1283/hsa-mir-1283-1 0.9993196
hsa-miR-1283/hsa-mir-1283-2       hsa-miR-1283/hsa-mir-1283-2 0.9993196
hsa-miR-1298-5p/hsa-mir-1298     hsa-miR-1298-5p/hsa-mir-1298 0.9999837
hsa-miR-1298-3p/hsa-mir-1298     hsa-miR-1298-3p/hsa-mir-1298 0.9991730
hsa-miR-1303/hsa-mir-1303           hsa-miR-1303/hsa-mir-1303 0.9996615
hsa-miR-1323/hsa-mir-1323           hsa-miR-1323/hsa-mir-1323 0.9998760
hsa-miR-135a-5p/hsa-mir-135a-1 hsa-miR-135a-5p/hsa-mir-135a-1 0.9982039
hsa-miR-135a-5p/hsa-mir-135a-2 hsa-miR-135a-5p/hsa-mir-135a-2 0.9981799
hsa-miR-141-5p/hsa-mir-141         hsa-miR-141-5p/hsa-mir-141 0.9983636
hsa-miR-141-3p/hsa-mir-141         hsa-miR-141-3p/hsa-mir-141 0.9962037
hsa-miR-142-3p/hsa-mir-142         hsa-miR-142-3p/hsa-mir-142 0.9822308
hsa-miR-146a-5p/hsa-mir-146a     hsa-miR-146a-5p/hsa-mir-146a 0.7951150
hsa-miR-182-5p/hsa-mir-182         hsa-miR-182-5p/hsa-mir-182 0.9885850
hsa-miR-182-3p/hsa-mir-182         hsa-miR-182-3p/hsa-mir-182 0.9997398
hsa-miR-183-5p/hsa-mir-183         hsa-miR-183-5p/hsa-mir-183 0.9975432
hsa-miR-183-3p/hsa-mir-183         hsa-miR-183-3p/hsa-mir-183 0.9994657
hsa-miR-1911-5p/hsa-mir-1911     hsa-miR-1911-5p/hsa-mir-1911 0.9976385
hsa-miR-1912/hsa-mir-1912           hsa-miR-1912/hsa-mir-1912 0.9956182
hsa-miR-193b-3p/hsa-mir-193b     hsa-miR-193b-3p/hsa-mir-193b 0.7862195
hsa-miR-19a-3p/hsa-mir-19a         hsa-miR-19a-3p/hsa-mir-19a 0.9732501
```

hsa-miR-200a-5p/hsa-mir-200a	hsa-miR-200a-5p/hsa-mir-200a	0.9974718
hsa-miR-200a-3p/hsa-mir-200a	hsa-miR-200a-3p/hsa-mir-200a	0.9964945
hsa-miR-200b-5p/hsa-mir-200b	hsa-miR-200b-5p/hsa-mir-200b	0.9678467
hsa-miR-200b-3p/hsa-mir-200b	hsa-miR-200b-3p/hsa-mir-200b	0.9996657
hsa-miR-203b-3p/hsa-mir-203b	hsa-miR-203b-3p/hsa-mir-203b	0.9809606
hsa-miR-2113/hsa-mir-2113	hsa-miR-2113/hsa-mir-2113	0.9561229
hsa-miR-221-5p/hsa-mir-221	hsa-miR-221-5p/hsa-mir-221	0.9663553
hsa-miR-26a-2-3p/hsa-mir-26a-2	hsa-miR-26a-2-3p/hsa-mir-26a-2	0.8116002
hsa-miR-32-3p/hsa-mir-32	hsa-miR-32-3p/hsa-mir-32	0.8022883
hsa-miR-33b-3p/hsa-mir-33b	hsa-miR-33b-3p/hsa-mir-33b	0.9601303
hsa-miR-367-5p/hsa-mir-367	hsa-miR-367-5p/hsa-mir-367	0.9994169
hsa-miR-371a-3p/hsa-mir-371a	hsa-miR-371a-3p/hsa-mir-371a	0.9462318
hsa-miR-372-5p/hsa-mir-372	hsa-miR-372-5p/hsa-mir-372	0.9641378
hsa-miR-372-3p/hsa-mir-372	hsa-miR-372-3p/hsa-mir-372	0.9676472
hsa-miR-374a-3p/hsa-mir-374a	hsa-miR-374a-3p/hsa-mir-374a	0.9889963
hsa-miR-429/hsa-mir-429	hsa-miR-429/hsa-mir-429	0.9997327
hsa-miR-448/hsa-mir-448	hsa-miR-448/hsa-mir-448	0.9998331
hsa-miR-4517/hsa-mir-4517	hsa-miR-4517/hsa-mir-4517	0.9851468
hsa-miR-4792/hsa-mir-4792	hsa-miR-4792/hsa-mir-4792	0.9821186
hsa-miR-498/hsa-mir-498	hsa-miR-498/hsa-mir-498	0.9990014
hsa-miR-512-5p/hsa-mir-512-1	hsa-miR-512-5p/hsa-mir-512-1	0.9989792
hsa-miR-512-3p/hsa-mir-512-1	hsa-miR-512-3p/hsa-mir-512-1	0.9977604
hsa-miR-512-5p/hsa-mir-512-2	hsa-miR-512-5p/hsa-mir-512-2	0.9989792
hsa-miR-512-3p/hsa-mir-512-2	hsa-miR-512-3p/hsa-mir-512-2	0.9977604
hsa-miR-515-5p/hsa-mir-515-1	hsa-miR-515-5p/hsa-mir-515-1	0.9981368
hsa-miR-515-5p/hsa-mir-515-2	hsa-miR-515-5p/hsa-mir-515-2	0.9981368
hsa-miR-516a-5p/hsa-mir-516a-1	hsa-miR-516a-5p/hsa-mir-516a-1	0.9928698
hsa-miR-516a-5p/hsa-mir-516a-2	hsa-miR-516a-5p/hsa-mir-516a-2	0.9928698
hsa-miR-516b-5p/hsa-mir-516b-1	hsa-miR-516b-5p/hsa-mir-516b-1	0.9994805
hsa-miR-516b-5p/hsa-mir-516b-2	hsa-miR-516b-5p/hsa-mir-516b-2	0.9998413
hsa-miR-517a-3p/hsa-mir-517a	hsa-miR-517a-3p/hsa-mir-517a	0.9997713
hsa-miR-517b-3p/hsa-mir-517b	hsa-miR-517b-3p/hsa-mir-517b	0.9997713
hsa-miR-517c-3p/hsa-mir-517c	hsa-miR-517c-3p/hsa-mir-517c	0.9993979
hsa-miR-518a-3p/hsa-mir-518a-1	hsa-miR-518a-3p/hsa-mir-518a-1	0.9986918
hsa-miR-518a-3p/hsa-mir-518a-2	hsa-miR-518a-3p/hsa-mir-518a-2	0.9986918
hsa-miR-518b/hsa-mir-518b	hsa-miR-518b/hsa-mir-518b	0.9996859
hsa-miR-518c-5p/hsa-mir-518c	hsa-miR-518c-5p/hsa-mir-518c	0.9989465
hsa-miR-518c-3p/hsa-mir-518c	hsa-miR-518c-3p/hsa-mir-518c	0.9998337
hsa-miR-518d-5p/hsa-mir-518d	hsa-miR-518d-5p/hsa-mir-518d	0.9999191
hsa-miR-518e-5p/hsa-mir-518e	hsa-miR-518e-5p/hsa-mir-518e	0.9989884
hsa-miR-518e-3p/hsa-mir-518e	hsa-miR-518e-3p/hsa-mir-518e	0.9996454
hsa-miR-518f-5p/hsa-mir-518f	hsa-miR-518f-5p/hsa-mir-518f	0.9999218
hsa-miR-519a-5p/hsa-mir-519a-1	hsa-miR-519a-5p/hsa-mir-519a-1	0.9990619
hsa-miR-519a-3p/hsa-mir-519a-1	hsa-miR-519a-3p/hsa-mir-519a-1	0.9982282
hsa-miR-519a-3p/hsa-mir-519a-2	hsa-miR-519a-3p/hsa-mir-519a-2	0.9982282
hsa-miR-519b-5p/hsa-mir-519b	hsa-miR-519b-5p/hsa-mir-519b	0.9989884
hsa-miR-519b-3p/hsa-mir-519b	hsa-miR-519b-3p/hsa-mir-519b	0.9992833
hsa-miR-519c-5p/hsa-mir-519c	hsa-miR-519c-5p/hsa-mir-519c	0.9989884
hsa-miR-519c-3p/hsa-mir-519c	hsa-miR-519c-3p/hsa-mir-519c	0.9990896
hsa-miR-519d-3p/hsa-mir-519d	hsa-miR-519d-3p/hsa-mir-519d	0.9993748
hsa-miR-520a-5p/hsa-mir-520a	hsa-miR-520a-5p/hsa-mir-520a	0.9998542
hsa-miR-520a-3p/hsa-mir-520a	hsa-miR-520a-3p/hsa-mir-520a	0.9994067
hsa-miR-520b/hsa-mir-520b	hsa-miR-520b/hsa-mir-520b	0.9986529
hsa-miR-520c-5p/hsa-mir-520c	hsa-miR-520c-5p/hsa-mir-520c	0.9999191
hsa-miR-520c-3p/hsa-mir-520c	hsa-miR-520c-3p/hsa-mir-520c	0.9987643
hsa-miR-520d-5p/hsa-mir-520d	hsa-miR-520d-5p/hsa-mir-520d	0.9994039
hsa-miR-520d-3p/hsa-mir-520d	hsa-miR-520d-3p/hsa-mir-520d	0.9988630
hsa-miR-520e/hsa-mir-520e	hsa-miR-520e/hsa-mir-520e	0.9998158
hsa-miR-520f-3p/hsa-mir-520f	hsa-miR-520f-3p/hsa-mir-520f	0.9992544
hsa-miR-520g-5p/hsa-mir-520g	hsa-miR-520g-5p/hsa-mir-520g	0.9996277
hsa-miR-520g-3p/hsa-mir-520g	hsa-miR-520g-3p/hsa-mir-520g	0.9988455
hsa-miR-520h/hsa-mir-520h	hsa-miR-520h/hsa-mir-520h	0.9987716
hsa-miR-522-5p/hsa-mir-522	hsa-miR-522-5p/hsa-mir-522	0.9989884
hsa-miR-522-3p/hsa-mir-522	hsa-miR-522-3p/hsa-mir-522	0.9991877

```
hsa-miR-523-5p/hsa-mir-523         hsa-miR-523-5p/hsa-mir-523 0.9989884
hsa-miR-523-3p/hsa-mir-523         hsa-miR-523-3p/hsa-mir-523 0.9988879
hsa-miR-524-5p/hsa-mir-524         hsa-miR-524-5p/hsa-mir-524 0.9999714
hsa-miR-524-3p/hsa-mir-524         hsa-miR-524-3p/hsa-mir-524 0.9980128
hsa-miR-525-5p/hsa-mir-525         hsa-miR-525-5p/hsa-mir-525 0.9995832
hsa-miR-526a/hsa-mir-526a-1       hsa-miR-526a/hsa-mir-526a-1 0.9999191
hsa-miR-526a/hsa-mir-526a-2       hsa-miR-526a/hsa-mir-526a-2 0.9999191
hsa-miR-526b-5p/hsa-mir-526b     hsa-miR-526b-5p/hsa-mir-526b 0.9994679
hsa-miR-548ah-5p/hsa-mir-548ah hsa-miR-548ah-5p/hsa-mir-548ah 0.7919416
hsa-miR-548ah-3p/hsa-mir-548ah hsa-miR-548ah-3p/hsa-mir-548ah 0.9697060
hsa-miR-548am-3p/hsa-mir-548am hsa-miR-548am-3p/hsa-mir-548am 0.9123712
hsa-miR-548f-3p/hsa-mir-548f-1 hsa-miR-548f-3p/hsa-mir-548f-1 0.9955869
hsa-miR-548f-3p/hsa-mir-548f-4 hsa-miR-548f-3p/hsa-mir-548f-4 0.9985424
hsa-miR-548p/hsa-mir-548p          hsa-miR-548p/hsa-mir-548p 0.9135189
hsa-miR-556-3p/hsa-mir-556         hsa-miR-556-3p/hsa-mir-556 0.9964808
hsa-miR-561-5p/hsa-mir-561         hsa-miR-561-5p/hsa-mir-561 0.9629218
hsa-miR-7974/hsa-mir-7974          hsa-miR-7974/hsa-mir-7974 0.9962122
hsa-miR-96-5p/hsa-mir-96            hsa-miR-96-5p/hsa-mir-96 0.9999629

[[5]]
                                                        NAME  MEM.SHIP
hsa-miR-107/hsa-mir-107                      hsa-miR-107/hsa-mir-107 0.9221811
hsa-miR-1180-3p/hsa-mir-1180            hsa-miR-1180-3p/hsa-mir-1180 0.9792134
hsa-miR-1227-3p/hsa-mir-1227            hsa-miR-1227-3p/hsa-mir-1227 0.9519243
hsa-miR-1247-5p/hsa-mir-1247            hsa-miR-1247-5p/hsa-mir-1247 0.9905833
hsa-miR-1248/hsa-mir-1248                  hsa-miR-1248/hsa-mir-1248 0.9879350
hsa-miR-125a-3p/hsa-mir-125a            hsa-miR-125a-3p/hsa-mir-125a 0.9772363
hsa-miR-126-5p/hsa-mir-126                hsa-miR-126-5p/hsa-mir-126 0.8015695
hsa-miR-126-3p/hsa-mir-126                hsa-miR-126-3p/hsa-mir-126 0.9548090
hsa-miR-1260a/hsa-mir-1260a              hsa-miR-1260a/hsa-mir-1260a 0.8712928
hsa-miR-1260b/hsa-mir-1260b              hsa-miR-1260b/hsa-mir-1260b 0.9008200
hsa-miR-1273d/hsa-mir-1273d              hsa-miR-1273d/hsa-mir-1273d 0.9797365
hsa-miR-1273g-3p/hsa-mir-1273g        hsa-miR-1273g-3p/hsa-mir-1273g 0.9785359
hsa-miR-128-3p/hsa-mir-128-1            hsa-miR-128-3p/hsa-mir-128-1 0.9898361
hsa-miR-128-3p/hsa-mir-128-2            hsa-miR-128-3p/hsa-mir-128-2 0.9943083
hsa-miR-1291/hsa-mir-1291                  hsa-miR-1291/hsa-mir-1291 0.8468690
hsa-miR-1296-5p/hsa-mir-1296            hsa-miR-1296-5p/hsa-mir-1296 0.9803247
hsa-miR-1306-5p/hsa-mir-1306            hsa-miR-1306-5p/hsa-mir-1306 0.9915009
hsa-miR-1307-3p/hsa-mir-1307            hsa-miR-1307-3p/hsa-mir-1307 0.9796124
hsa-miR-1343-3p/hsa-mir-1343            hsa-miR-1343-3p/hsa-mir-1343 0.9979470
hsa-miR-140-3p/hsa-mir-140                hsa-miR-140-3p/hsa-mir-140 0.9805221
hsa-miR-146b-5p/hsa-mir-146b            hsa-miR-146b-5p/hsa-mir-146b 0.9999527
hsa-miR-149-5p/hsa-mir-149                hsa-miR-149-5p/hsa-mir-149 0.9877905
hsa-miR-188-5p/hsa-mir-188                hsa-miR-188-5p/hsa-mir-188 0.9733501
hsa-miR-1908-5p/hsa-mir-1908            hsa-miR-1908-5p/hsa-mir-1908 0.9913885
hsa-miR-193a-5p/hsa-mir-193a            hsa-miR-193a-5p/hsa-mir-193a 0.9807344
hsa-miR-196a-5p/hsa-mir-196a-1        hsa-miR-196a-5p/hsa-mir-196a-1 0.9730400
hsa-miR-196a-5p/hsa-mir-196a-2        hsa-miR-196a-5p/hsa-mir-196a-2 0.9576564
hsa-miR-197-3p/hsa-mir-197                hsa-miR-197-3p/hsa-mir-197 0.8321653
hsa-miR-199b-5p/hsa-mir-199b            hsa-miR-199b-5p/hsa-mir-199b 0.8988989
hsa-miR-210-3p/hsa-mir-210                hsa-miR-210-3p/hsa-mir-210 0.8450248
hsa-miR-2110/hsa-mir-2110                  hsa-miR-2110/hsa-mir-2110 0.8731308
hsa-miR-26b-3p/hsa-mir-26b                hsa-miR-26b-3p/hsa-mir-26b 0.9458140
hsa-miR-27b-5p/hsa-mir-27b                hsa-miR-27b-5p/hsa-mir-27b 0.7521569
hsa-miR-3065-5p/hsa-mir-3065            hsa-miR-3065-5p/hsa-mir-3065 0.9255444
hsa-miR-30c-2-3p/hsa-mir-30c-2        hsa-miR-30c-2-3p/hsa-mir-30c-2 0.9773211
hsa-miR-3127-3p/hsa-mir-3127            hsa-miR-3127-3p/hsa-mir-3127 0.9817040
hsa-miR-3188/hsa-mir-3188                  hsa-miR-3188/hsa-mir-3188 0.9619329
hsa-miR-320c/hsa-mir-320c-1              hsa-miR-320c/hsa-mir-320c-1 0.7598922
hsa-miR-320c/hsa-mir-320c-2              hsa-miR-320c/hsa-mir-320c-2 0.7623246
hsa-miR-323a-3p/hsa-mir-323a            hsa-miR-323a-3p/hsa-mir-323a 0.9881288
hsa-miR-324-3p/hsa-mir-324                hsa-miR-324-3p/hsa-mir-324 0.9988118
hsa-miR-339-3p/hsa-mir-339                hsa-miR-339-3p/hsa-mir-339 0.7810893
hsa-miR-340-3p/hsa-mir-340                hsa-miR-340-3p/hsa-mir-340 0.9993712
```

hsa-miR-342-5p/hsa-mir-342	hsa-miR-342-5p/hsa-mir-342	0.9718513
hsa-miR-342-3p/hsa-mir-342	hsa-miR-342-3p/hsa-mir-342	0.9147119
hsa-miR-3605-3p/hsa-mir-3605	hsa-miR-3605-3p/hsa-mir-3605	0.7696339
hsa-miR-361-3p/hsa-mir-361	hsa-miR-361-3p/hsa-mir-361	0.8573756
hsa-miR-3613-5p/hsa-mir-3613	hsa-miR-3613-5p/hsa-mir-3613	0.9881144
hsa-miR-3615/hsa-mir-3615	hsa-miR-3615/hsa-mir-3615	0.9810972
hsa-miR-3651/hsa-mir-3651	hsa-miR-3651/hsa-mir-3651	0.9549179
hsa-miR-381-3p/hsa-mir-381	hsa-miR-381-3p/hsa-mir-381	0.9168266
hsa-miR-3909/hsa-mir-3909	hsa-miR-3909/hsa-mir-3909	0.9624546
hsa-miR-409-3p/hsa-mir-409	hsa-miR-409-3p/hsa-mir-409	0.9999970
hsa-miR-421/hsa-mir-421	hsa-miR-421/hsa-mir-421	0.9995660
hsa-miR-423-5p/hsa-mir-423	hsa-miR-423-5p/hsa-mir-423	0.9656020
hsa-miR-4448/hsa-mir-4448	hsa-miR-4448/hsa-mir-4448	0.9897025
hsa-miR-4449/hsa-mir-4449	hsa-miR-4449/hsa-mir-4449	0.8720678
hsa-miR-4488/hsa-mir-4488	hsa-miR-4488/hsa-mir-4488	0.9999991
hsa-miR-455-5p/hsa-mir-455	hsa-miR-455-5p/hsa-mir-455	0.8977847
hsa-miR-455-3p/hsa-mir-455	hsa-miR-455-3p/hsa-mir-455	0.8482420
hsa-miR-4728-3p/hsa-mir-4728	hsa-miR-4728-3p/hsa-mir-4728	0.9513783
hsa-miR-484/hsa-mir-484	hsa-miR-484/hsa-mir-484	0.9805820
hsa-miR-500b-3p/hsa-mir-500b	hsa-miR-500b-3p/hsa-mir-500b	0.9567633
hsa-miR-508-5p/hsa-mir-508	hsa-miR-508-5p/hsa-mir-508	0.8026625
hsa-miR-532-5p/hsa-mir-532	hsa-miR-532-5p/hsa-mir-532	0.9382906
hsa-miR-532-3p/hsa-mir-532	hsa-miR-532-3p/hsa-mir-532	0.8512450
hsa-miR-543/hsa-mir-543	hsa-miR-543/hsa-mir-543	0.9897456
hsa-miR-550a-5p/hsa-mir-550a-1	hsa-miR-550a-5p/hsa-mir-550a-1	0.9991675
hsa-miR-550a-3-5p/hsa-mir-550a-1	hsa-miR-550a-3-5p/hsa-mir-550a-1	0.9991675
hsa-miR-550a-5p/hsa-mir-550a-2	hsa-miR-550a-5p/hsa-mir-550a-2	0.9991675
hsa-miR-550a-3-5p/hsa-mir-550a-2	hsa-miR-550a-3-5p/hsa-mir-550a-2	0.9991675
hsa-miR-574-5p/hsa-mir-574	hsa-miR-574-5p/hsa-mir-574	0.8660486
hsa-miR-625-5p/hsa-mir-625	hsa-miR-625-5p/hsa-mir-625	0.9642176
hsa-miR-642a-5p/hsa-mir-642a	hsa-miR-642a-5p/hsa-mir-642a	0.9802768
hsa-miR-642a-3p/hsa-mir-642a	hsa-miR-642a-3p/hsa-mir-642a	0.9822368
hsa-miR-6511a-3p/hsa-mir-6511a-1	hsa-miR-6511a-3p/hsa-mir-6511a-1	0.9990005
hsa-miR-6511a-3p/hsa-mir-6511a-2	hsa-miR-6511a-3p/hsa-mir-6511a-2	0.9990005
hsa-miR-6511a-3p/hsa-mir-6511a-3	hsa-miR-6511a-3p/hsa-mir-6511a-3	0.9990005
hsa-miR-6511a-3p/hsa-mir-6511a-4	hsa-miR-6511a-3p/hsa-mir-6511a-4	0.9990005
hsa-miR-6511b-3p/hsa-mir-6511b-1	hsa-miR-6511b-3p/hsa-mir-6511b-1	0.9903945
hsa-miR-6511b-3p/hsa-mir-6511b-2	hsa-miR-6511b-3p/hsa-mir-6511b-2	0.9901786
hsa-miR-654-3p/hsa-mir-654	hsa-miR-654-3p/hsa-mir-654	0.9633188
hsa-miR-671-5p/hsa-mir-671	hsa-miR-671-5p/hsa-mir-671	0.8896201
hsa-miR-6808-3p/hsa-mir-6808	hsa-miR-6808-3p/hsa-mir-6808	0.7527699
hsa-miR-6866-5p/hsa-mir-6866	hsa-miR-6866-5p/hsa-mir-6866	0.9957405
hsa-miR-760/hsa-mir-760	hsa-miR-760/hsa-mir-760	0.9691277
hsa-miR-766-5p/hsa-mir-766	hsa-miR-766-5p/hsa-mir-766	0.9897916
hsa-miR-766-3p/hsa-mir-766	hsa-miR-766-3p/hsa-mir-766	0.8049953
hsa-miR-769-3p/hsa-mir-769	hsa-miR-769-3p/hsa-mir-769	0.9087774
hsa-miR-877-5p/hsa-mir-877	hsa-miR-877-5p/hsa-mir-877	0.9133595
hsa-miR-885-5p/hsa-mir-885	hsa-miR-885-5p/hsa-mir-885	0.9778039
hsa-miR-943/hsa-mir-943	hsa-miR-943/hsa-mir-943	0.8011582
