# Supplementary material for: Identification of the miRNAome of early mesoderm progenitor cells and cardiomyocytes derived from human pluripotent stem cells
Source: Sci Rep. 2018 May 23;8:8072. doi: 10.1038/s41598-018-26156-3 (PMC5966391; doi:10.1038/s41598-018-26156-3)
Supplement: Supplementary file 8 — Supplementary file 7 [file 41598_2018_26156_MOESM8_ESM.pdf]

Identification of the miRNAome of early mesoderm progenitor cells and cardiomyocytes derived from human pluripotent stem cells

Ximena Garate[1], Alejandro La Greca[1], Gabriel Neiman[1], Carolina Blüguermann[1], Natalia L. Santín Velazque[1], Lucía N. Moro[1], Carlos Luzzani[1], Elida Scassa[1], Gustavo E. Sevlever[1], Leonardo Romorini[1], and Santiago G. Miriuka[1*]

[1]LIAN-CONICET, FLENI - Ruta 9 km 52.5 - Belen de Escobar, Provincia de Buenos Aires, Argentina
[*]smiriuka@fleni.org.ar

ABSTRACT

miRBase precursor : hsa-mir-302a
Total read count : 458107
hsa-miR-302a-5p read count 409544
hsa-miR-302a-3p read count 44324
remaining reads : 4238

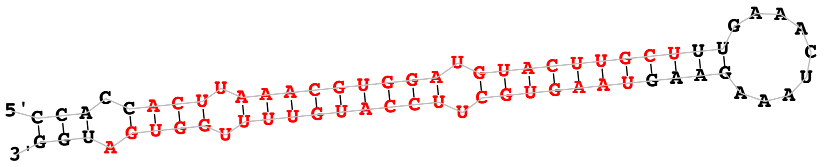

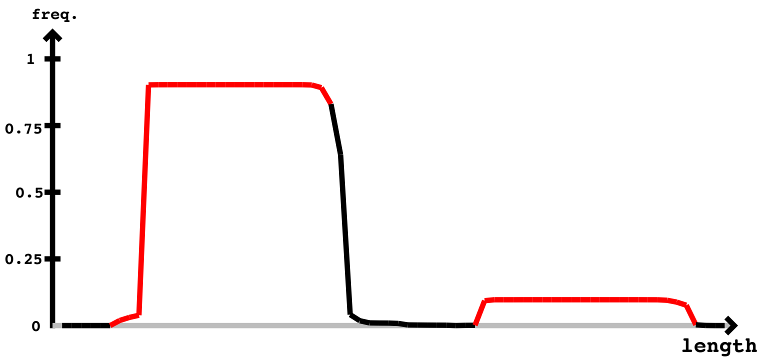

hsa-miR-302a-5p

hsa-miR-302a-3p

5'- ccaccacuuaaacguggauguacuugcuuugaaacuaaagaaguaagugcuuccauguuuuggugaugg -3'	exp		
(((.((((.((((((((.((((((((((.........)))))))))).))))))))).)))).)))	reads	mm	sample
....cacuuaaacguggauguacuug..	1	0	seq
.....acuuaaGcguggauguac...	1	1	seq
.....acuuaaacguggauguac...	25	0	seq
.....acuuaaacguggauguacu..	25	0	seq
.....acuuaaacguggauguacuu...	448	0	seq
.....acuuaaacguggauguaUuu...	1	1	seq
.....acCuaaacguggauguacuu...	1	1	seq
.....acuuUaacguggauguacuu...	1	1	seq
.....Gcuuaaacguggauguacuu...	1	1	seq
.....aAuuaaacguggauguacuu...	1	1	seq
.....aGuuaaacguggauguacuu...	1	1	seq
.....acuuaaGcguggauguacuu...	2	1	seq
.....acuuaaacguggauguacuG...	6	1	seq
.....acuuaaacguggGuguacuu...	4	1	seq
.....acuuaaacAuggauguacuu...	1	1	seq
.....acuuaaacguggaugCacuu...	1	1	seq
.....acuAaaacguggauguacuu...	1	1	seq
.....acuuaaacguggauguacuA...	1	1	seq
.....acuuaaacguggauguacuC...	6	1	seq
.....acuuaaacguggauguGcuu...	1	1	seq
.....acuuaaacgugUauguacuug..	1	1	seq
.....acuuaaacguggGuguacuug..	15	1	seq
.....acuCaaacguggauguacuug..	6	1	seq
.....acuuaaacguAgauguacuug..	1	1	seq
.....acuuaaGcguggauguacuug..	11	1	seq
.....acuuaaUcguggauguacuug..	1	1	seq
.....acAuaaacguggauguacuug..	2	1	seq
.....acuuaaacguggaCguacuug..	6	1	seq
.....acuuaaacAuggauguacuug..	4	1	seq
.....Ucuuaaacguggauguacuug..	2	1	seq
.....acCuaaacguggauguacuug..	8	1	seq
.....acuuaaacguggaGguacuug..	6	1	seq
.....acuuaaacguggauguacuuC..	4	1	seq
.....acuuaaacguggauguaAuug..	1	1	seq

```
     hsa-miR-302a-5p
                                                  hsa-miR-302a-3p
ccaccacuuaaacguggauguacuugcuuugaaacuaaagaaguaagugcuuccauguuuuggugaugg

.....aUuuaaacguggauguacuug............................................   1      1   seq
.....acuuaaacguggauguacAug............................................   2      1   seq
.....acuuaaacguggauguacCug............................................   9      1   seq
.....acuuUaacguggauguacuug............................................   1      1   seq
.....acuuaaacguggauguacuuU............................................   2      1   seq
.....acuuaaacgugAauguacuug............................................   2      1   seq
.....acuuaaaUguggauguacuug............................................   5      1   seq
.....acuuaaacguUgauguacuug............................................   2      1   seq
.....acuuaaacguggauguacuuA............................................   7      1   seq
.....aAuuaaacguggauguacuug............................................   1      1   seq
.....acuuaaacguggauguacuGg............................................   6      1   seq
.....acuuaaacguggUuguacuug............................................   2      1   seq
.....acuuGaacguggauguacuug............................................   10     1   seq
.....acuuaaacguggauAuacuug............................................   2      1   seq
.....acuuaaacgCggauguacuug............................................   8      1   seq
.....acuuaaacguggaAguacuug............................................   1      1   seq
.....acuuaaacguggauguGcuug............................................   7      1   seq
.....acuuaaacguggauguaUuug............................................   3      1   seq
.....aGuuaaacguggauguacuug............................................   3      1   seq
.....acuuaaacguggauUuacuug............................................   1      1   seq
.....acuuaaacguggauguacuug............................................   3066   0   seq
.....acuuaGacguggauguacuug............................................   6      1   seq
.....Gcuuaaacguggauguacuug............................................   12     1   seq
.....acuuaaacguggauguacuCg............................................   13     1   seq
.....acuuaaacguggaugCacuug............................................   8      1   seq
.....acuuaaacgAggauguacuug............................................   1      1   seq
.....acuuaaacguggCuguacuug............................................   1      1   seq
.....acuuaaacguggaGguacuugc...........................................   3      1   seq
.....acuuaaacgCggauguacuugc...........................................   12     1   seq
.....acuuaaacguggauguacuAgc...........................................   1      1   seq
.....acuAaaacguggauguacuugc...........................................   1      1   seq
.....acuuaaacguUgauguacuugc...........................................   6      1   seq
.....acuuaaacAuggauguacuugc...........................................   3      1   seq
.....Gcuuaaacguggauguacuugc...........................................   21     1   seq
.....acuuCaacguggauguacuugc...........................................   1      1   seq
.....acuuGaacguggauguacuugc...........................................   8      1   seq
.....acuuaaGcguggauguacuugc...........................................   7      1   seq
.....acCuaaacguggauguacuugc...........................................   18     1   seq
.....acuuaaacgGggauguacuugc...........................................   1      1   seq
.....acuuaaCcguggauguacuugc...........................................   3      1   seq
.....acuuaaacgugAauguacuugc...........................................   4      1   seq
.....acuuaaacguggauguUcuugc...........................................   1      1   seq
.....acuuaaacguggauguacuGgc...........................................   3      1   seq
.....acuuaGacguggauguacuugc...........................................   12     1   seq
.....acuuaaacgugUauguacuugc...........................................   1      1   seq
.....acuuaaacguggauAuacuugc...........................................   2      1   seq
.....acuuaaacguggauguacuCgc...........................................   8      1   seq
.....acGuaaacguggauguacuugc...........................................   1      1   seq
.....acuuaaacguggGuguacuugc...........................................   21     1   seq
.....aAuuaaacguggauguacuugc...........................................   2      1   seq
.....aGuuaaacguggauguacuugc...........................................   4      1   seq
.....acuuaaacguggaAguacuugc...........................................   3      1   seq
.....acuuaaacguggauguacuuUc...........................................   1      1   seq
.....Ucuuaaacguggauguacuugc...........................................   4      1   seq
.....acuuaaacgAggauguacuugc...........................................   2      1   seq
.....acuuaaacguggauguacuugc...........................................   4673   0   seq
.....acuuaaacguggauguacAugc...........................................   6      1   seq
.....acuuaaacguAgauguacuugc...........................................   3      1   seq
.....acuuaaacguggauguacuuAc...........................................   4      1   seq
.....acuuaaacguggauguaUuugc...........................................   6      1   seq
.....acuuaaacguggaugCacuugc...........................................   4      1   seq
.....aUuuaaacguggauguacuugc...........................................   2      1   seq
.....acuuaaacguggUuguacuugc...........................................   2      1   seq
.....acuuaaacguggaCguacuugc...........................................   16     1   seq
.....acuuaaacguggauguGcuugc...........................................   7      1   seq
.....acuuaaaUguggauguacuugc...........................................   2      1   seq
.....acuuaaacguggauguacuugU...........................................   7      1   seq
.....acuuaaacguggauguacCugc...........................................   11     1   seq
.....acAuaaacguggauguacuugc...........................................   4      1   seq
```

hsa-miR-302a-5p

hsa-miR-302a-3p

ccaccacuuaaacguggauguacuugcuuugaaacuaaagaaguaagugcuuccauguuuuggugaugg

.....Ccuuaaacguggauguacuugc..	2	1	seq
.....acuuaaacguggauguCcuugc..	2	1	seq
.....acuCaaacguggauguacuugc..	10	1	seq
.....acuuaaacCuggauguacuugc..	1	1	seq
.....acuuaaacguggauguacuugcC...	1	1	seq
.....acuuaaacguggauguacuugcu...	22	0	seq
.....acuCaaacguggauguacuugcu...	1	1	seq
.....acuuaaacguggauguacCugcu...	1	1	seq
.....acuuaaacguggauguacuugcG...	1	1	seq
.....acuuaaacguggauguacuugcA...	1	1	seq
.....acuuaaacguggauguacuugcuA..	3	1	seq
.....acCuaaacguggauguacuugcuu..	1	1	seq
.....aUuuaaacguggauguacuugcuu..	1	1	seq
.....acuuaaacguggauguacuugcuuu.......................................	1	0	seq
......cuuaaacguggauguacu...	4	0	seq
......cuuaaacguggauguacuu..	10	0	seq
......cuuaaacguggauguacuug...	200	0	seq
......Uuuaaacguggauguacuug...	1	1	seq
......cuuaaacgGggauguacuug...	1	1	seq
......cCuaaacguggauguacuug...	1	1	seq
......cuuaaacguggGuguacuug...	1	1	seq
......cuuaaacguAgauguacuug...	1	1	seq
......Guuaaacguggauguacuug...	1	1	seq
......cuuaaacguggauguacuAgc..	1	1	seq
......cuuaaacguggauguacuugU..	10	1	seq
......cuuaaacguggauguCcuugc..	1	1	seq
......cuuaaacguggauguacAugc..	2	1	seq
......Auuaaacguggauguacuugc..	2	1	seq
......cuuaaacguggauguacuugc..	4187	0	seq
......cuuaaacgGggauguacuugc..	2	1	seq
......cuuaaacguggauguacCugc..	9	1	seq
......cuuaaacgCggauguacuugc..	3	1	seq
......cuuaaacgugAauguacuugc..	2	1	seq
......cuAaaacguggauguacuugc..	1	1	seq
......Uuuaaacguggauguacuugc..	7	1	seq
......cuuaaacguggauguacGugc..	1	1	seq
......cuuaaacguggaugAacuugc..	1	1	seq
......cuuaaacguggauguUcuugc..	1	1	seq
......cuuaaacguAgauguacuugc..	5	1	seq
......cuuaaacguggaCguacuugc..	13	1	seq
......cGuaaacguggauguacuugc..	1	1	seq
......cuCaaacguggauguacuugc..	4	1	seq
......cuuaaacguggaGguacuugc..	2	1	seq
......cuuaaacguggauguacuuAc..	6	1	seq
......cuuaaacguggauguacuugA..	4	1	seq
......cuuaaaUguggauguacuugc..	7	1	seq
......cuuaaGcguggauguacuugc..	8	1	seq
......cuuaaacguUgauguacuugc..	2	1	seq
......cCuaaacguggauguacuugc..	14	1	seq
......cuuaaCcguggauguacuugc..	1	1	seq
......cuuaaacguggauguGcuugc..	16	1	seq
......cuuaaacgAggauguacuugc..	1	1	seq
......cuuaGacguggauguacuugc..	10	1	seq
......cuuaaacguggauguacuCgc..	12	1	seq
......cuuaaacguggauguaUuugc..	2	1	seq
......cuuaaacguggGuguacuugc..	10	1	seq
......cuuaaacguggauguacuuCc..	1	1	seq
......cuuaaacguggaAguacuugc..	1	1	seq
......cuuaaacguggauguacuGgc..	1	1	seq
......cuuaaacguggaugCacuugc..	11	1	seq
......cuuaaacguCgauguacuugc..	1	1	seq
......Guuaaacguggauguacuugc..	6	1	seq
......cuuGaacguggauguacuugc..	5	1	seq
......cuuaaacAuggauguacuugc..	3	1	seq
......cAuaaacguggauguacuugc..	3	1	seq
......cuuaaacguggauAuacuugc..	6	1	seq
......cuuaaacguggaugAacuugcu...	1	1	seq
......cuuaaacguggauguUcuugcu...	1	1	seq
......cuuaaacguggauguacuugUu...	2	1	seq

hsa-miR-302a-5p

hsa-miR-302a-3p

ccaccacuuaaacguggauguacuugcuuugaaacuaaagaaguaagugcuuccauguuuuggugaugg

......cuuaaacgCggauguacuugcu...	3	1	seq
......cuuaaacguggaAguacuugcu...	1	1	seq
......cuuaaacguggauguacuugcC...	2	1	seq
......cuuaaacguggauguGcuugcu...	1	1	seq
......cuuaaacguggauguacuugcu...	548	0	seq
......cuuaaacguggauguacuugcG...	4	1	seq
......cuuaaacguggaugCacuugcu...	2	1	seq
......cuuaaacguggGuguacuugcu...	1	1	seq
......cuuaaacguggauguacuugAu...	1	1	seq
......cuuaGacguggauguacuugcu...	1	1	seq
......cuuaaGcguggauguacuugcu...	1	1	seq
......cuuaaacguAgauguacuugcu...	2	1	seq
......cuuaaacguggauguacuuAcu...	1	1	seq
......cCuaaacguggauguacuugcu...	2	1	seq
......cGuaaacguggauguacuugcu...	2	1	seq
......cuuaaacguggaCguacuugcu...	4	1	seq
......cAuaaacguggauguacuugcu...	1	1	seq
......cuCaaacguggauguacuugcu...	2	1	seq
......cuuaaaUguggauguacuugcu...	1	1	seq
......cuuaaacguggauguacuugGu...	1	1	seq
......cuuaaacguggauguacuCgcu...	2	1	seq
......cuuaaacguggauguacuugcuA..	8	1	seq
......cuuaGacguggauguacuugcuu..	1	1	seq
......cuuaaacguggauguacuugcuG..	1	1	seq
......cGuaaacguggauguacuugcuu..	1	1	seq
......cuuaaacguggauguacuugcuu..	35	0	seq
......cuuaaacguggauguacuugcuC..	1	1	seq
......cuuaaacguggauguacuugcuuG.......................................	1	1	seq
......cuuaaacguggauguacuugcuuu.......................................	3	0	seq
......cuuaaacguggauguacuugcuuA.......................................	2	1	seq
......cCuaaacguggauguacuugcuuu.......................................	1	1	seq
......cuuaaacguggauguacuugcuuuC......................................	1	1	seq
.......uuaaacguggauguacuu..	3	0	seq
.......uuaaacguggauguacuug...	43	0	seq
.......uuaaacguggauguacCugc..	5	1	seq
.......uuaaacguggauguGcuugc..	3	1	seq
.......uuaaacguggauguacuuCc..	1	1	seq
.......Guaaacguggauguacuugc..	1	1	seq
.......uuaaCcguggauguacuugc..	1	1	seq
.......uuaaacgAggauguacuugc..	1	1	seq
.......uuaaacguCgauguacuugc..	1	1	seq
.......uuaaacguggauguacuuAc..	1	1	seq
.......uuaCacguggauguacuugc..	1	1	seq
.......uuaaacguggaugCacuugc..	1	1	seq
.......uuaaacguggaAguacuugc..	1	1	seq
.......uuaaacguggauguacuugU..	1	1	seq
.......uuaaacgugAauguacuugc..	1	1	seq
.......uuaaacgCggauguacuugc..	2	1	seq
.......uuaaacguggauguacuugc..	904	0	seq
.......uuaaacAuggauguacuugc..	2	1	seq
.......Cuaaacguggauguacuugc..	10	1	seq
.......uuaaacguggGuguacuugc..	4	1	seq
.......uuGaacguggauguacuugc..	2	1	seq
.......uuaaacguAgauguacuugc..	1	1	seq
.......uuaaacguggauguacuAgc..	1	1	seq
.......uuaaacguggauguacuCgc..	2	1	seq
.......uuaaaUguggauguacuugc..	1	1	seq
.......uCaaacguggauguacuugc..	1	1	seq
.......uuaaacguggaugAacuugc..	1	1	seq
.......uuaaacguggaCguacuugc..	6	1	seq
.......uuaGacguggauguacuugc..	1	1	seq
.......uuaaacguggGuguacuugcu...	7	1	seq
.......uuaaacguggauguacGugcu...	1	1	seq
.......uuaaacguggUuguacuugcu...	2	1	seq
.......uuaaacguggauguaUuugcu...	3	1	seq
.......uuaaacgugAauguacuugcu...	1	1	seq
.......uuaaacguggauguacuugcC...	19	1	seq
.......uuaaacguggauguacuuUcu...	1	1	seq
.......uuaaCcguggauguacuugcu...	1	1	seq

hsa-miR-302a-5p

hsa-miR-302a-3p

ccaccacuuaaacguggauguacuugcuuugaaacuaaagaaguaagugcuuccauguuuuggugaugg

.......uuaaacguggauguacuugcG...	11	1	seq
.......uuaaacguggaugAacuugcu...	2	1	seq
.......Guaaacguggauguacuugcu...	2	1	seq
.......uuaaacguggauguacuAgcu...	1	1	seq
.......uCaaacguggauguacuugcu...	9	1	seq
.......uuaaacguggaugCacuugcu...	6	1	seq
.......uuaaacguggaAguacuugcu...	2	1	seq
.......Auaaacguggauguacuugcu...	3	1	seq
.......uuaaacguggauguacuugAu...	3	1	seq
.......uuaaacguggaCguacuugcu...	5	1	seq
.......uuUaacguggauguacuugcu...	2	1	seq
.......uuaaacguggauguacuCgcu...	7	1	seq
.......uuaaacguggauguacuugGu...	2	1	seq
.......uuaaacguggauCuacuugcu...	1	1	seq
.......uAaaacguggauguacuugcu...	2	1	seq
.......uuaaacAuggauguacuugcu...	2	1	seq
.......uuaaacgCggauguacuugcu...	2	1	seq
.......uuaCacguggauguacuugcu...	3	1	seq
.......uuaaacguggauguacuugcA...	6	1	seq
.......uuaaacguggauguacuuAcu...	3	1	seq
.......uuaUacguggauguacuugcu...	1	1	seq
.......uuaaacguggauguacuugUu...	4	1	seq
.......uuaaaUguggauguacuugcu...	2	1	seq
.......uuaaacguUgauguacuugcu...	1	1	seq
.......uuaaacguCgauguacuugcu...	3	1	seq
.......uuaaacguggauguacuugcu...	2389	0	seq
.......uuaaacCuggauguacuugcu...	1	1	seq
.......uuaaacguAgauguacuugcu...	1	1	seq
.......uuaaaAguggauguacuugcu...	3	1	seq
.......uuGaacguggauguacuugcu...	3	1	seq
.......uuaaaGguggauguacuugcu...	1	1	seq
.......uuaaacguggauguGcuugcu...	5	1	seq
.......uuaaacguggauguacCugcu...	10	1	seq
.......uuaaGcguggauguacuugcu...	6	1	seq
.......uuaaacguggauAuacuugcu...	4	1	seq
.......uuaaUcguggauguacuugcu...	2	1	seq
.......uuaaacguggauguacAugcu...	2	1	seq
.......uuaGacguggauguacuugcu...	7	1	seq
.......Cuaaacguggauguacuugcu...	28	1	seq
.......uuaaacguggauguacuugcuC..	1	1	seq
.......Auaaacguggauguacuugcuu..	8	1	seq
.......uuaaacguggauguacuugGuu..	1	1	seq
.......uuaaacguggauguacAugcuu..	1	1	seq
.......uuaGacguggauguacuugcuu..	1	1	seq
.......uuaaacguggauguacuugcuu..	63	0	seq
.......Guaaacguggauguacuugcuu..	1	1	seq
.......uuaaacguggauguacuugcuA..	3	1	seq
.......uuaaacAuggauguacuugcuu..	1	1	seq
.......uuaaacguggauguacuugUuu..	1	1	seq
.......uuaaacguggauguacuugcAu..	1	1	seq
.......uAaaacguggauguacuugcuu..	2	1	seq
.......Cuaaacguggauguacuugcuu..	57	1	seq
.......Cuaaacguggauguacuugcuuu.......................................	3	1	seq
.......uuaaacguggauguacuugcuuu.......................................	6	0	seq
.......uuaaacguggauguacuugcuuA.......................................	10	1	seq
.......uuaaacguggauguacuugcuuug......................................	2	0	seq
.......Cuaaacguggauguacuugcuuug......................................	1	1	seq
.......uuaaacguggauguacuugcuuuga.....................................	1	0	seq
........uaaacgugUauguacuug...	1	1	seq
........uaaacguggauguacuCg...	2	1	seq
........Gaaacguggauguacuug...	1	1	seq
........uaaacguggauguGcuug...	2	1	seq
........uaaacguggauguacuug...	1004	0	seq
........uaaacguggauguacuuC...	2	1	seq
........uaaacguggauguacuGg...	3	1	seq
........uaaacgugAauguacuug...	1	1	seq
........uaaacguggauguacCug...	1	1	seq
........uaaacguggUuguacuug...	1	1	seq
........uaaacguggaugCacuug...	3	1	seq

hsa-miR-302a-5p

hsa-miR-302a-3p

```
ccaccacuuaaacguggauguacuugcuuugaaacuaaagaaguaagugcuuccauguuuuggugaugg

........uaaacguggauAuacuug...........................................   1       1       seq
........uaaacguggaCguacuug...........................................   3       1       seq
........uaaacgAggauguacuug...........................................   2       1       seq
........uGaacguggauguacuug...........................................   3       1       seq
........uaaacguAgauguacuug...........................................   2       1       seq
........uaaacgCggauguacuug...........................................   2       1       seq
........Caaacguggauguacuug...........................................   1       1       seq
........uaaGcguggauguacuug...........................................   1       1       seq
........uaaacguggGuguacuug...........................................   1       1       seq
........uUaacguggauguacuug...........................................   1       1       seq
........uaaacguggauguacAug...........................................   1       1       seq
........uaGacguggauguacuug...........................................   4       1       seq
........uaaacguggauguacuugG..........................................   6       1       seq
........uaaacgugAauguacuugc..........................................   8       1       seq
........uaaacguCgauguacuugc..........................................   6       1       seq
........uaaacguggauguCcuugc..........................................   2       1       seq
........uaaacgAggauguacuugc..........................................   4       1       seq
........uaaacguggauguacuugc..........................................   17129   0       seq
........Aaaacguggauguacuugc..........................................   13      1       seq
........uGaacguggauguacuugc..........................................   65      1       seq
........uaaacguAgauguacuugc..........................................   21      1       seq
........uaaacguggauguacuuAc..........................................   17      1       seq
........uaUacguggauguacuugc..........................................   7       1       seq
........uaCacguggauguacuugc..........................................   5       1       seq
........uaaacgugUauguacuugc..........................................   3       1       seq
........uaaacguggaugAacuugc..........................................   4       1       seq
........uCaacguggauguacuugc..........................................   3       1       seq
........uaaacguggauguacuGgc..........................................   8       1       seq
........uaaacguggaugGacuugc..........................................   8       1       seq
........uUaacguggauguacuugc..........................................   8       1       seq
........uaaacAuggauguacuugc..........................................   20      1       seq
........uaaacguUgauguacuugc..........................................   1       1       seq
........uaaacguggauguacCugc..........................................   43      1       seq
........uaaacguggaugCacuugc..........................................   37      1       seq
........uaaacgCggauguacuugc..........................................   42      1       seq
........uaaacguggauguaUuugc..........................................   10      1       seq
........uaaacUuggauguacuugc..........................................   1       1       seq
........uaaacguggauguUcuugc..........................................   5       1       seq
........uaaacguggauUuacuugc..........................................   1       1       seq
........uaaacguggauguacuuUc..........................................   1       1       seq
........uaaacguggaAguacuugc..........................................   8       1       seq
........uaaacguggauguacuugU..........................................   29      1       seq
........uaaaUguggauguacuugc..........................................   15      1       seq
........uaaacguggauguacAugc..........................................   8       1       seq
........uaaacguggauguacuugA..........................................   3       1       seq
........uaaacguggaGguacuugc..........................................   6       1       seq
........uaaacguggauguacuCgc..........................................   54      1       seq
........uaaacguggaCguacuugc..........................................   55      1       seq
........uaaCcguggauguacuugc..........................................   9       1       seq
........uaaacguggauAuacuugc..........................................   21      1       seq
........Gaaacguggauguacuugc..........................................   19      1       seq
........uaaUcguggauguacuugc..........................................   3       1       seq
........uaaacguggauguGcuugc..........................................   44      1       seq
........uaaacgugCauguacuugc..........................................   1       1       seq
........uaaaGguggauguacuugc..........................................   1       1       seq
........uaaacguggauguacuuCc..........................................   3       1       seq
........uaGacguggauguacuugc..........................................   44      1       seq
........Caaacguggauguacuugc..........................................   40      1       seq
........uaaacgGggauguacuugc..........................................   3       1       seq
........uaaGcguggauguacuugc..........................................   50      1       seq
........uaaacguggUuguacuugc..........................................   8       1       seq
........uaaacguggauguacGugc..........................................   3       1       seq
........uaaacguggauguacuAgc..........................................   4       1       seq
........uaaacCuggauguacuugc..........................................   1       1       seq
........uaaacguggGuguacuugc..........................................   58      1       seq
........uaaacguggauguacuugcG.........................................   366     1       seq
........uaaUcguggauguacuugcu.........................................   14      1       seq
........uaaacguggauguacuugcA.........................................   59      1       seq
........uUaacguggauguacuugcu.........................................   45      1       seq
```

hsa-miR-302a-5p

hsa-miR-302a-3p

ccaccacuuaaacguggauguacuugcuuugaaacuaaagaaguaagugcuuccauguuuuggugaugg

........uaaacgugUauguacuugcu...	22	1	seq
........uaaacguggauguaGuugcu...	4	1	seq
........uaaacguggauguacCugcu...	194	1	seq
........uaaacguggaugCacuugcu...	132	1	seq
........uaaacguggUuguacuugcu...	42	1	seq
........uaaacguggauguacuuAcu...	43	1	seq
........uaaacguggGuguacuugcu...	288	1	seq
........uaaaGguggauguacuugcu...	4	1	seq
........uaaacguggauguacGugcu...	7	1	seq
........uaaacAuggauguacuugcu...	120	1	seq
........uaaGcguggauguacuugcu...	194	1	seq
........uaUacguggauguacuugcu...	24	1	seq
........uaaacguggCuguacuugcu...	12	1	seq
........uaaacCuggauguacuugcu...	3	1	seq
........uaaacgGggauguacuugcu...	10	1	seq
........uaaacguggauguacAugcu...	36	1	seq
........uaaacgCggauguacuugcu...	185	1	seq
........uaaacgAggauguacuugcu...	12	1	seq
........uaaacguCgauguacuugcu...	10	1	seq
........uaaacguggauguGcuugcu...	273	1	seq
........uaaacguggauguacuugUu...	91	1	seq
........uaCacguggauguacuugcu...	15	1	seq
........uaaacguggauguacuugcC...	427	1	seq
........uaaacguggauguacuugcu...	78998	0	seq
........uaaacguggauguCcuugcu...	11	1	seq
........uaaacguggauguacuAgcu...	38	1	seq
........uaaaAguggauguacuugcu...	1	1	seq
........uaaCcguggauguacuugcu...	32	1	seq
........uaaacguUgauguacuugcu...	25	1	seq
........uaaacguggauguUcuugcu...	37	1	seq
........uaaacguggaCguacuugcu...	256	1	seq
........uCaacguggauguacuugcu...	13	1	seq
........uaaacguggauguacuugAu...	42	1	seq
........uaaacguggauUuacuugcu...	3	1	seq
........uaaacguggaGguacuugcu...	14	1	seq
........Gaaacguggauguacuugcu...	81	1	seq
........uaaacguggauguacuCgcu...	230	1	seq
........uaaacgugCauguacuugcu...	4	1	seq
........Aaaacguggauguacuugcu...	63	1	seq
........uaaacUuggauguacuugcu...	8	1	seq
........uGaacguggauguacuugcu...	301	1	seq
........uaaacguggauAuacuugcu...	72	1	seq
........uaaacguggauCuacuugcu...	4	1	seq
........uaaacguggaAguacuugcu...	28	1	seq
........uaaacguggauguacuuCcu...	4	1	seq
........uaaacguggaugAacuugcu...	20	1	seq
........uaaaUguggauguacuugcu...	77	1	seq
........uaaacguggauguacuugGu...	8	1	seq
........uaaacguggauguaUuugcu...	63	1	seq
........uaaacguggauguaAuugcu...	4	1	seq
........uaaacguAgauguacuugcu...	79	1	seq
........uaaacguggauguacuGgcu...	30	1	seq
........uaaacgugAauguacuugcu...	41	1	seq
........Caaacguggauguacuugcu...	173	1	seq
........uaGacguggauguacuugcu...	217	1	seq
........uaaacguggauguacuuUcu...	4	1	seq
........uaaacguggaugGacuugcu...	39	1	seq
........uaaacguggauguacGugcuu..	31	1	seq
........uaaacgugAauguacuugcuu..	169	1	seq
........uaaacguggauguacuugcGu..	242	1	seq
........uCaacguggauguacuugcuu..	44	1	seq
........uaaacguggauguacuugcuA..	203	1	seq
........Aaaacguggauguacuugcuu..	157	1	seq
........uaaacguggaAguacuugcuu..	83	1	seq
........uaaacguggauAuacuugcuu..	228	1	seq
........uGaacguggauguacuugcuu..	939	1	seq
........uaaacguggauguacuugUuu..	326	1	seq
........uUaacguggauguacuugcuu..	121	1	seq
........uaaacgugUauguacuugcuu..	68	1	seq

hsa-miR-302a-5p

hsa-miR-302a-3p

```
ccaccacuuaaacguggauguacuugcuuugaaacuaaagaaguaagugcuuccauguuuuggugaugg

........uaaaAguggauguacuugcuu.........................................   7        1   seq
........uaaacguggGuguacuugcuu.........................................   1021     1   seq
........uaaacguggauguacuugcCu.........................................   624      1   seq
........uaaacguggauguacuugcAu.........................................   150      1   seq
........uaaacguggauCuacuugcuu.........................................   10       1   seq
........uaaacguggauguCcuugcuu.........................................   32       1   seq
........uaaacguggauguaGuugcuu.........................................   2        1   seq
........uaaacguAgauguacuugcuu.........................................   227      1   seq
........Caaacguggauguacuugcuu.........................................   591      1   seq
........uaaacguggauguacuugAuu.........................................   29       1   seq
........uaaacguggauUuacuugcuu.........................................   19       1   seq
........uaaacguggauguacuuCcuu.........................................   15       1   seq
........uaaacguggauguaUuugcuu.........................................   189      1   seq
........uaaacguggauguacuGgcuu.........................................   91       1   seq
........uaaacguggauguacuuAcuu.........................................   186      1   seq
........uaaacguggauguacuCgcuu.........................................   678      1   seq
........uaGacguggauguacuugcuu.........................................   744      1   seq
........uaaacgGggauguacuugcuu.........................................   35       1   seq
........uaaacguggauguacuuUcuu.........................................   19       1   seq
........uaaacguggauguacCugcuu.........................................   684      1   seq
........uaaacguggauguacuAgcuu.........................................   114      1   seq
........uaaacguggauguUcuugcuu.........................................   103      1   seq
........uaaacguggauguacuugcuC.........................................   848      1   seq
........uaaacguggaugCacuugcuu.........................................   465      1   seq
........uaaaGguggauguacuugcuu.........................................   8        1   seq
........uaaacguggauguGcuugcuu.........................................   804      1   seq
........uaaacguggaGguacuugcuu.........................................   26       1   seq
........Gaaacguggauguacuugcuu.........................................   230      1   seq
........uaaacgCggauguacuugcuu.........................................   541      1   seq
........uaaacguUgauguacuugcuu.........................................   69       1   seq
........uaaacUuggauguacuugcuu.........................................   17       1   seq
........uaaGcguggauguacuugcuu.........................................   705      1   seq
........uaaacguggCuguacuugcuu.........................................   28       1   seq
........uaaUcguggauguacuugcuu.........................................   42       1   seq
........uaaacguggaCguacuugcuu.........................................   854      1   seq
........uaaacguggauguacuugcuG.........................................   783      1   seq
........uaaacguggauguaAuugcuu.........................................   15       1   seq
........uaaaUguggauguacuugcuu.........................................   245      1   seq
........uaaacguggaugGacuugcuu.........................................   98       1   seq
........uaaacgugCauguacuugcuu.........................................   19       1   seq
........uaaacAuggauguacuugcuu.........................................   376      1   seq
........uaaacguggauguacuugcuu.........................................   259397   0   seq
........uaaacguggauguacAugcuu.........................................   113      1   seq
........uaaacguCgauguacuugcuu.........................................   27       1   seq
........uaCacguggauguacuugcuu.........................................   40       1   seq
........uaaacCuggauguacuugcuu.........................................   12       1   seq
........uaaCcguggauguacuugcuu.........................................   141      1   seq
........uaUacguggauguacuugcuu.........................................   79       1   seq
........uaaacguggauguacuugGuu.........................................   14       1   seq
........uaaacguggUuguacuugcuu.........................................   109      1   seq
........uaaacgAggauguacuugcuu.........................................   51       1   seq
........uaaacguggaugAacuugcuu.........................................   62       1   seq
........uaaacgugAauguacuugcuuu........................................   12       1   seq
........uaaacguggauguacuugcuuG........................................   53       1   seq
........uaaacguggauguacuugcCuu........................................   28       1   seq
........uaaacguAgauguacuugcuuu........................................   9        1   seq
........uaGacguggauguacuugcuuu........................................   22       1   seq
........uaaacguggauguaUuugcuuu........................................   6        1   seq
........uaaacguggauguacGugcuuu........................................   1        1   seq
........uaaacguggauguacuCgcuuu........................................   31       1   seq
........uaaacguggauguacuugcGuu........................................   4        1   seq
........uaaacguggaugGacuugcuuu........................................   5        1   seq
........uaaacguggUuguacuugcuuu........................................   2        1   seq
........uaaacguggauguCcuugcuuu........................................   2        1   seq
........uaaacguggaCguacuugcuuu........................................   30       1   seq
........uaaacgugUauguacuugcuuu........................................   3        1   seq
........uaaacguggauguacuGgcuuu........................................   1        1   seq
........uaaacgGggauguacuugcuuu........................................   1        1   seq
........uaaCcguggauguacuugcuuu........................................   1        1   seq
```

hsa-miR-302a-5p

hsa-miR-302a-3p

ccaccacuuaaacguggauguacuugcuuugaaacuaaagaaguaagugcuuccauguuuuggugaugg

........uaaacgguggauguaAuugcuuu......................................	1	1	seq
........uaaacgguggauguacuugcuuA......................................	204	1	seq
........uGaacgguggauguacuugcuuu......................................	34	1	seq
........Caaacgguggauguacuugcuuu......................................	21	1	seq
........uaaacgguggCuguacuugcuuu......................................	2	1	seq
........uaaacgguggauguacuugAuuu......................................	1	1	seq
........uaUacgguggauguacuugcuuu......................................	4	1	seq
........uaaacgguggauguacAugcuuu......................................	5	1	seq
........uaaacgguggauguacCugcuuu......................................	24	1	seq
........uCaacgguggauguacuugcuuu......................................	3	1	seq
........uaCacgguggauguacuugcuuu......................................	2	1	seq
........uaaacgguggauguacuugcAuu......................................	3	1	seq
........uaaacgguggauguacuugUuuu......................................	11	1	seq
........uaaacgguggaAguacuugcuuu......................................	4	1	seq
........uaaaUgguggauguacuugcuuu......................................	8	1	seq
........uaaacgguggaugCacuugcuuu......................................	21	1	seq
........uaaacgguggauguacuuAcuuu......................................	8	1	seq
........uaaGcgguggauguacuugcuuu......................................	20	1	seq
........uaaacgguggauguacuugcuGu......................................	11	1	seq
........uaaacgguggauAuacuugcuuu......................................	8	1	seq
........Gaaacgguggauguacuugcuuu......................................	11	1	seq
........uaaacguCgauguacuugcuuu......................................	1	1	seq
........uaaacUuggauguacuugcuuu......................................	2	1	seq
........uaaacgguggauguacuugcuAu......................................	5	1	seq
........uaaacgguggauguacuuUcuuu......................................	1	1	seq
........uaaacgguggauguUcuugcuuu......................................	2	1	seq
........uaaacgguggauguacuuCcuuu......................................	1	1	seq
........uaaacgguggGuguacuugcuuu......................................	30	1	seq
........uaaacAuggauguacuugcuuu......................................	18	1	seq
........uUaacgguggauguacuugcuuu......................................	4	1	seq
........uaaacgguggauguacuugcuCu......................................	9	1	seq
........uaaacgguggauguacuugcuuu......................................	9532	0	seq
........uaaacgguggauguacuAgcuuu......................................	4	1	seq
........uaaacguUgauguacuugcuuu......................................	5	1	seq
........uaaacgguggauguGcuugcuuu......................................	34	1	seq
........Aaaacgguggauguacuugcuuu......................................	5	1	seq
........uaaacgguggaugAacuugcuuu......................................	3	1	seq
........uaaacgguggauguacuugcuuC......................................	98	1	seq
........uaaacgCggauguacuugcuuu......................................	23	1	seq
........uaaacgAggauguacuugcuuu......................................	7	1	seq
........uaaacgguggauguacuugcuCug.....................................	3	1	seq
........uaaacgguggauguacuugcCuug.....................................	13	1	seq
........uaaacgguggauguacCugcuuug.....................................	8	1	seq
........uGaacgguggauguacuugcuuug.....................................	14	1	seq
........uaaacgguggauguacuuAcuuug.....................................	4	1	seq
........uaaacgguggauAuacuugcuuug.....................................	2	1	seq
........uaaacgCggauguacuugcuuug.....................................	6	1	seq
........Aaaacgguggauguacuugcuuug.....................................	2	1	seq
........uaaacgguggaugCacuugcuuug.....................................	2	1	seq
........uaaacgAggauguacuugcuuug.....................................	3	1	seq
........uaaacgguggauguacuugcGuug.....................................	3	1	seq
........uaaacguUgauguacuugcuuug.....................................	2	1	seq
........uaaacgguggauguacuugcuuuC.....................................	29	1	seq
........uaaGcgguggauguacuugcuuug.....................................	9	1	seq
........uaUacgguggauguacuugcuuug.....................................	1	1	seq
........uaaacgguggauguGcuugcuuug.....................................	8	1	seq
........uaaacgguggauguacAugcuuug.....................................	2	1	seq
........uaaacguAgauguacuugcuuug.....................................	4	1	seq
........uaaUcgguggauguacuugcuuug.....................................	2	1	seq
........uaaacgguggauguacGugcuuug.....................................	2	1	seq
........uaaacgguggauguacuugcuuAg.....................................	7	1	seq
........uaaaAgguggauguacuugcuuug.....................................	1	1	seq
........uaaacgguggauguacuugcuuuU.....................................	18	1	seq
........uaaacgguggGuguacuugcuuug.....................................	14	1	seq
........uaaacggugAauguacuugcuuug.....................................	3	1	seq
........uaaacgguggauguacuugAuuug.....................................	1	1	seq
........uaaacgguggauguacuugcuuCg.....................................	13	1	seq
........uaaacgguggauguUcuugcuuug.....................................	3	1	seq
........uaGacgguggauguacuugcuuug.....................................	7	1	seq

```
     hsa-miR-302a-5p
                                        hsa-miR-302a-3p
ccaccacuuaaacguggauguacuugcuuugaaacuaaagaaguaagugcuuccauguuuuggugaugg

........uaaacguggauguacuCgcuuug......................................    8      1     seq
........uaaacguggauguacuugUuug......................................    1      1     seq
........uaaacguggauguacuugcuuGg......................................    5      1     seq
........uaaacguggauguacuuUcuuug......................................    1      1     seq
........uaaacguggauguaUuugcuuug......................................    5      1     seq
........uaaacgugUauguacuugcuuug......................................    2      1     seq
........uaaacguggaugGacuugcuuug......................................    1      1     seq
........uUaacguggauguacuugcuuug......................................    2      1     seq
........uaaacguggauguacuugcuuuA......................................    188    1     seq
........uaaacguggaAguacuugcuuug......................................    1      1     seq
........uaaacguggauguacuugcuGug......................................    1      1     seq
........Caaacguggauguacuugcuuug......................................    7      1     seq
........uaaacguggauguacuAgcuuug......................................    2      1     seq
........uaaacguggauguacuugcuAug......................................    2      1     seq
........uaaacguggauguacuugcAuug......................................    4      1     seq
........uaaacguggaCguacuugcuuug......................................    8      1     seq
........uaaacguggauguacuugcuuug......................................    3256   0     seq
........Gaaacguggauguacuugcuuug......................................    6      1     seq
........uaaCcguggauguacuugcuuug......................................    5      1     seq
........uaaaUguggauguacuugcuuug......................................    5      1     seq
........uaaacAuggauguacuugcuuug......................................    4      1     seq
........uaaacguggauguacuugcuuuUa.....................................    2      1     seq
........uaaacgCggauguacuugcuuuga.....................................    2      1     seq
........uaaacguggauguacuugcuuugC.....................................    3      1     seq
........uaaacguggauguacuugcuuuAa.....................................    24     1     seq
........uaaacguggauguacuugcuuugU.....................................    5      1     seq
........uaaacguggauguacuugcuuuga.....................................    201    0     seq
........uaaGcguggauguacuugcuuuga.....................................    1      1     seq
........uaaacguggauguGcuugcuuuga.....................................    1      1     seq
........uaaacguggauguacuugcuCuga.....................................    2      1     seq
........uaaacguggauguacuugcuuugG.....................................    4      1     seq
........uaaacgugCauguacuugcuuuga.....................................    1      1     seq
........uaaacguggauguacuugcGuuga.....................................    1      1     seq
........uaGacguggauguacuugcuuugaa....................................    3      1     seq
........Caaacguggauguacuugcuuugaa....................................    1      1     seq
........uaaacguggauguacuugcuuuAaa....................................    1      1     seq
........uaaacguggauguacuugcuuugaG....................................    4      1     seq
........uaaacguggauguacuugcuuugaC....................................    1      1     seq
........uaaacguggauguacuugcuuugCa....................................    1      1     seq
........uaaacguggauguacuugcuuugaa....................................    85     0     seq
........uaaacguggauguacuugcuuugGa....................................    1      1     seq
........uaaacguggauguacuugcCuugaa....................................    1      1     seq
........uaaacguggauguacuugcuuugaaa...................................    532    0     seq
........uaaGcguggauguacuugcuuugaaa...................................    1      1     seq
........uaaacguggGuguacuugcuuugaaa...................................    2      1     seq
........uaaacguggauguacuugcGuugaaa...................................    1      1     seq
........uaaacguggauguacCugcuuugaaa...................................    1      1     seq
........Caaacguggauguacuugcuuugaaa...................................    1      1     seq
........uaaacguggaCguacuugcuuugaaa...................................    5      1     seq
........uGaacguggauguacuugcuuugaaa...................................    5      1     seq
........uaaacguggauguacuugcCuugaaa...................................    1      1     seq
........uaaacguggauguacuugcuuugaGa...................................    4      1     seq
........uaaacguggauguacuugcuuugaaC...................................    1      1     seq
........uaaacAuggauguacuugcuuugaaa...................................    1      1     seq
........uaaacguggauguacuugcuuugaaG...................................    2      1     seq
........uaaacguggauguacuugcuuuAaaa...................................    4      1     seq
........uaaacguggauguacuugcuuCgaaa...................................    2      1     seq
........uaaacguggauguacuCgcuuugaaa...................................    1      1     seq
........uaaacguggaAguacuugcuuugaaa...................................    1      1     seq
........uaaacguggauguacuugcuCugaaa...................................    1      1     seq
........uaaacguggauguacuugcuuugGaa...................................    4      1     seq
........uaaacgCggauguacuugcuuugaaac..................................    7      1     seq
........uaaacguggauguGcuugcuuugaaac..................................    5      1     seq
........uaaacguggauguUcuugcuuugaaac..................................    1      1     seq
........uaaacguggauguacCugcuuugaaac..................................    8      1     seq
........uaaacguggauguacuGgcuuugaaac..................................    1      1     seq
........uaGacguggauguacuugcuuugaaac..................................    8      1     seq
........uaaacguggauguacuugcGuugaaac..................................    1      1     seq
........uaaacgugCauguacuugcuuugaaac..................................    1      1     seq
```

hsa-miR-302a-5p

hsa-miR-302a-3p

ccaccacuuaaacguggauguacuugcuuugaaacuaaagaaguaagugcuuccauguuuuggugaugg

........uaaacgugAauguacuugcuuugaaac..................................	1	1	seq
........uaaacguggaCguacuugcuuugaaac..................................	10	1	seq
........uaaacguggUuguacuugcuuugaaac..................................	4	1	seq
........uaUacguggauguacuugcuuugaaac..................................	1	1	seq
........uaaacgugUauguacuugcuuugaaac..................................	3	1	seq
........uaaacguggauguacuugcuuuAaaac..................................	6	1	seq
........uaaacguggauguacuugcuCugaaac..................................	5	1	seq
........uaaacguggauguacuugUuuugaaac..................................	3	1	seq
........uaaacguggauguacuugcuuugaGac..................................	8	1	seq
........uaaacguggauguacuugcuuugaaUc..................................	1	1	seq
........Aaaacguggauguacuugcuuugaaac..................................	1	1	seq
........uaaacguggauguacuugcuuugaCac..................................	1	1	seq
........uaaacguggauguaUuugcuuugaaac..................................	1	1	seq
........uaaacguggauguacuugcuuugaaaA..................................	1	1	seq
........uaaacguggaugAacuugcuuugaaac..................................	1	1	seq
........uaaacgGggauguacuugcuuugaaac..................................	2	1	seq
........uaaacguggauguacuugcuuugaUac..................................	1	1	seq
........uaaaUguggauguacuugcuuugaaac..................................	1	1	seq
........uaaacguggauguacuugcuuugaaac..................................	2380	0	seq
........uaaacguggauguacuugcuuuGaac..................................	9	1	seq
........uaaacguggauguaAuugcuuugaaac..................................	1	1	seq
........uaaacguggauguacuugcCuugaaac..................................	12	1	seq
........Caaacguggauguacuugcuuugaaac..................................	4	1	seq
........uaaacguggauguacuugcuuugaaaU..................................	1	1	seq
........uaaacguggaugGacuugcuuugaaac..................................	3	1	seq
........uaaacguggauguacuCgcuuugaaac..................................	3	1	seq
........uaaacguCgauguacuugcuuugaaac..................................	1	1	seq
........uaaacgAggauguacuugcuuugaaac..................................	1	1	seq
........uaaacguggauguacuugcuuAgaaac..................................	1	1	seq
........uaaacguAgauguacuugcuuugaaac..................................	2	1	seq
........uaaacguggauguacuugcuGugaaac..................................	3	1	seq
........uaaGcguggauguacuugcuuugaaac..................................	5	1	seq
........uaaacguggaugCacuugcuuugaaac..................................	8	1	seq
........uaaacguggauguaGuugcuuugaaac..................................	1	1	seq
........uaaacguggauguacuugcuuugaaGc..................................	15	1	seq
........uaaacguggauguacuugcuuCgaaac..................................	5	1	seq
........uaaacAuggauguacuugcuuugaaac..................................	3	1	seq
........uGaacguggauguacuugcuuugaaac..................................	6	1	seq
........uaaacguggauguacuugcAuugaaac..................................	2	1	seq
........uaaacguggGuguacuugcuuugaaac..................................	8	1	seq
........uaaacguggauguacAugcuuugaaac..................................	1	1	seq
........uGaacguggauguacuugcuuugaaacu.................................	1	1	seq
........uaaacguggauguacAugcuuugaaacu.................................	2	1	seq
........uaaacguggGuguacuugcuuugaaacu.................................	1	1	seq
........uaaacguggauguGcuugcuuugaaacu.................................	1	1	seq
........uaaacguggCuguacuugcuuugaaacu.................................	1	1	seq
........uaaacguggauguacCugcuuugaaacu.................................	1	1	seq
........uaaacguggauguacuugcCuugaaacu.................................	2	1	seq
........uaaacguggauguacuugcuuugaaacu.................................	166	0	seq
........uaaacguggauguacuugcuCugaaacu.................................	1	1	seq
........uaaacguggauguacuugcuuugaGacu.................................	2	1	seq
........uaaacguggauguaUuugcuuugaaacu.................................	1	1	seq
........uaaacguggauguacuugcuuugaaacG.................................	5	1	seq
........uaaacgCggauguacuugcuuugaaacu.................................	1	1	seq
........uaaacguggauguacuugcuuugaaGcu.................................	1	1	seq
........uaaCcguggauguacuugcuuugaaacu.................................	1	1	seq
........uaaacgAggauguacuugcuuugaaacua................................	1	1	seq
........uaaacguggauguacuugcuuuGaacua................................	1	1	seq
........uaaacguggauguacuugcuuCgaaacua................................	1	1	seq
........uaaacguggGuguacuugcuuugaaacua................................	1	1	seq
........uaaacguggauguacuugcuuugaaacua................................	124	0	seq
........uaGacguggauguacuugcuuugaaacuaa...............................	2	1	seq
........uaaacguggauguacuugcuuugaaacuaU...............................	3	1	seq
........uaaacguggauguacuugcuuugaaacuaC...............................	1	1	seq
........uaaacguggauAuacuugcuuugaaacuaa...............................	1	1	seq
........uaaacguggauguacuugcCuugaaacuaa...............................	1	1	seq
........uaaGcguggauguacuugcuuugaaacuaa...............................	1	1	seq
........uaaacguggauguacCugcuuugaaacuaa...............................	1	1	seq
........uaaacguggauguacuugcuuugaaacuaa...............................	90	0	seq

hsa-miR-302a-5p

hsa-miR-302a-3p

ccaccacuuaaacguggauguacuugcuuugaaacuaaagaaguaagugcuuccauguuuuggugaugg

........uaaacguggaAguacuugcuuugaaacuaaa..............................	1	1	seq
........uaaacguggauguacuugcuuugaaacuaaG..............................	1	1	seq
........uaaacguggauguacuugcuuugaaacuaGa..............................	1	1	seq
........uaaacguggauguacuCgcuuugaaacuaaa..............................	1	1	seq
........uaaacguggauguacuugcuuugaaacCaaa..............................	1	1	seq
........uaaacguggauguacuugcuCugaaacuaaa..............................	1	1	seq
........uaaacguggauguacuugcuuugGaacuaaa..............................	1	1	seq
........uaaacguggauguacuugcuuugaaacuaaU..............................	1	1	seq
........uaaacgugUauguacuugcuuugaaacuaaa..............................	1	1	seq
........uaaacguggauguacuugcuuugaaacuaaa..............................	51	0	seq
........uaaacguggauguacuugcuuugaaacuaaGg.............................	3	1	seq
........Gaaacguggauguacuugcuuugaaacuaaag.............................	2	1	seq
........uaaacguggaugGacuugcuuugaaacuaaag.............................	1	1	seq
........uaaacguggauguacuugcuuugaaacuaaag.............................	555	0	seq
........uaaacguggauguacAugcuuugaaacuaaag.............................	1	1	seq
........uaaacguggauguaUuugcuuugaaacuaaag.............................	1	1	seq
........uaaacguggauguacuugcuuugaaacuaaaC.............................	1	1	seq
........uaaacguggauguacuugcuuCgaaacuaaag.............................	1	1	seq
........uaaacguggauguacuugcuuugaaGcuaaag.............................	2	1	seq
........uaaacguggauguacuugcuuugGaacuaaag.............................	2	1	seq
........uaaacguggauguacuugcuuugUaacuaaag.............................	1	1	seq
........uaaacguggauguacuugcuuugaaacuGaag.............................	2	1	seq
........Caaacguggauguacuugcuuugaaacuaaag.............................	4	1	seq
........uaaacguggauguacuugcuuugaaacuaGag.............................	3	1	seq
........uaGacguggauguacuugcuuugaaacuaaag.............................	4	1	seq
........uaaacguggauguacuugcuuugaaUcuaaag.............................	1	1	seq
........uaaacguggauguUcuugcuuugaaacuaaag.............................	1	1	seq
........uaaGcguggauguacuugcuuugaaacuaaag.............................	1	1	seq
........uaaacguggauguacuugcuAugaaacuaaag.............................	1	1	seq
........uaaacguggaCguacuugcuuugaaacuaaag.............................	3	1	seq
........uaaacguggauguacuCgcuuugaaacuaaag.............................	1	1	seq
........uaaacgugUauguacuugcuuugaaacuaaag.............................	1	1	seq
........uaaacguggauguacuugcuuugaGacuaaag.............................	3	1	seq
........uaaacguggauguacuugcuGugaaacuaaag.............................	1	1	seq
........uaaacguggGuguacuugcuuugaaacuaaag.............................	4	1	seq
........uGaacguggauguacuugcuuugaaacuaaag.............................	3	1	seq
........uaaacgCggauguacuugcuuugaaacuaaag.............................	2	1	seq
........uaaacguggauguacuugcuuugaaacuaaaga............................	5	0	seq
........uaaacguggauguacuugcuuugaaacuaaagU............................	1	1	seq
.........Uaacguggauguacuugc..	2	1	seq
.........aaacguggauguacuugc..	10	0	seq
.........aaacguggauguacuugcu...	38	0	seq
.........aaGcguggauguacuugcu...	1	1	seq
.........Gaacguggauguacuugcu...	1	1	seq
.........Uaacguggauguacuugcu...	32	1	seq
.........aaacguggGuguacuugcu...	1	1	seq
.........Uaacguggauguacuugcuu..	69	1	seq
.........aGacguggauguacuugcuu..	1	1	seq
.........aaacguggauguacuugcuu..	66	0	seq
.........Uaacguggauguacuugcuuu.......................................	5	1	seq
.........aaacguggauguacuugcuuu.......................................	2	0	seq
.........Uaacguggauguacuugcuuug......................................	1	1	seq
.........aaacguggauguacuugcuuug......................................	2	0	seq
.........Uaacguggauguacuugcuuugaaa...................................	1	1	seq
.........Uaacguggauguacuugcuuugaaac..................................	1	1	seq
..........aacguggauguacuugcu...	6	0	seq
..........aacguggauguacuugcuu..	17	0	seq
..........aacguggauguacuugcuuu.......................................	2	0	seq
..........aacguggauguacuugcuuug......................................	2	0	seq
...........Ccguggauguacuugcuu..	2	1	seq
...........acguggauguGcuugcuu..	1	1	seq
...........acguggauguacuugcCu..	1	1	seq
...........acguggauguacuAgcuu..	1	1	seq
...........acgGggauguacuugcuu..	1	1	seq
...........acguggauguacuugcuu..	32	0	seq
............cguggauguacuugcuuu.......................................	6	0	seq
............cguggauguacuugcuuug......................................	2	0	seq
............cguggauguacuugcuuuga.....................................	1	0	seq
............cguggauguacuugcuuCgaaac..................................	1	1	seq

hsa-miR-302a-5p

hsa-miR-302a-3p

ccaccacuuaaacguggauguacuugcuuugaaacuaaagaaguaagugcuuccauguuuuggugaugg

............cguggauguacuugcuuugaaac..................................	3	0	seq
.............guggauguacuugcuuug......................................	5	0	seq
.............guggauguacuugcuuuga.....................................	1	0	seq
.............guggauguacuugcuuugaaa...................................	3	0	seq
.............guggauguacuugcuuugGaa...................................	1	1	seq
.............guggauguacuugcuuugaaac..................................	3	0	seq
.............guggauguacuugcuuugaaacua................................	1	0	seq
.............guggauguacuugcuuugaaacuaaag.............................	2	0	seq
..............uggauguacuugcuuugaaac..................................	2	0	seq
..............uggauguacuugcCuugaaac..................................	1	1	seq
..............uggauguacuugcuuugaaacuaaaga............................	1	0	seq
...............ggauguacuugcuuugaaa...................................	1	0	seq
................gauguacuugcuuugaaa...................................	1	0	seq
................gauguacuugcuuugaaacuaaaga............................	1	0	seq
...................guacuugcuuugaaacuaa...............................	1	0	seq
........................ugcuuugaaacuaaagaa...........................	1	0	seq
....aguaagugcuuccauguu..........	1	0	seq
....aguaagugcuuccauguuu.........	9	0	seq
....aguaagugcuuccauguuG.........	0	1	seq
....aguGagugcuuccauguuu.........	0	1	seq
....aguaagugcuuccauguuuu........	15	0	seq
....Uguaagugcuuccauguuuu........	0	1	seq
....aAuaagugcuuccauguuuu........	0	1	seq
....aguaagAgcuuccauguuuug.......	1	1	seq
....aUuaagugcuuccauguuuug.......	1	1	seq
....aguaagugcuuccaugGuug.......	2	1	seq
....aguaagugcuucGauguuuug.......	1	1	seq
....Gguaagugcuuccauguuuug.......	2	1	seq
....aguaagugcCuccauguuuug.......	1	1	seq
....aguaagugcuuccauguuuGg.......	2	1	seq
....aguaagugcuuAcauguuuug.......	1	1	seq
....aguaagugUuuccauguuuug.......	1	1	seq
....aguaGgugcuuccauguuuug.......	1	1	seq
....aguaagugcAuccauguuuug.......	1	1	seq
....Uguaagugcuuccauguuuug.......	2	1	seq
....aguaagugcuuccGuguuuug.......	1	1	seq
....aguaagugcuuccauguuuug.......	297	0	seq
....aguaagugcuuccauguuuugA......	1	1	seq
....aguaagCgcuuccauguuuugg......	1	1	seq
....aguaagugcuuccauguuuugg......	32	0	seq
....aguaagugcuuccauguuuuggA.....	2	1	seq
....aAuaagugcuuccauguuuuggu.....	1	1	seq
....aAuaagugcuuccauguuuuggug....	8	1	seq
....aAuaagugcuuccauguuuuggugа...	1	1	seq
....guaagugcuuccauguuC.........	0	1	seq
....Cuaagugcuuccauguuu.........	0	1	seq
....guaagugcuuccauguuuug.......	3	0	seq
....guaagugcuuccauguuuugg......	14	0	seq
....Auaagugcuuccauguuuugg......	1	1	seq
....guaagugcCuccauguuuugg......	1	1	seq
....Cuaagugcuuccauguuuuggu.....	6	1	seq
....Auaagugcuuccauguuuuggu.....	2	1	seq
....Uuaagugcuuccauguuuuggug....	2	1	seq
....Cuaagugcuuccauguuuuggug....	20	1	seq
....Auaagugcuuccauguuuuggug....	2	1	seq
....Cuaagugcuuccauguuuuggugа...	2	1	seq
....uaagugcuuccauAuuuu........	0	1	seq
....uaagugcuuccauguuuu........	24	0	seq
....uaagugcuuccauguuuCg.......	2	1	seq
....uaagugcuuccauguuuGg.......	2	1	seq
....uaagugcCuccauguuuug.......	2	1	seq
....uaagugcuuccaCguuuug.......	2	1	seq
....uaagugcuuUcauguuuug.......	1	1	seq
....uaagugcuuccauguuCug.......	2	1	seq
....uaagugcuuccGuguuuug.......	1	1	seq
....uaagugcuuccaGguuuug.......	1	1	seq
....uaagugcuuccauguuuAg.......	267	1	seq
....uaagugcuuccauguuuug.......	186	0	seq
....uaagugcuCccauguuuug.......	1	1	seq

```
                   hsa-miR-302a-5p
                                                  hsa-miR-302a-3p
ccaccacuuaaacguggauguacuugcuuugaaacuaaagaaguaagugcuuccauguuuuggugaugg

...........................................uaagugcuuccauguuuuU.......     0       1       seq
...........................................uaagugcuuccauguAuug.......     1       1       seq
...........................................uGagugcuuccauguuuug.......     1       1       seq
...........................................uaagugcuucUauguuuugg......     3       1       seq
...........................................uaagugcuuccCuguuuugg......     1       1       seq
...........................................uaagugcCuccauguuuugg......     7       1       seq
...........................................Caagugcuuccauguuuugg......     4       1       seq
...........................................uaagugAuuccauguuuugg......     1       1       seq
...........................................uaagugcuuccauguuuAgg......     2       1       seq
...........................................uaagugcuuccauguuuugA......     2       1       seq
...........................................uaagugcuuccauguuuugg......     2851    0       seq
...........................................uaagugcuuccauAuuuugg......     2       1       seq
...........................................uaagugcuuccaugGuuugg......     4       1       seq
...........................................uaaAugcuuccauguuuugg......     2       1       seq
...........................................uaGgugcuuccauguuuugg......     11      1       seq
...........................................uaUgugcuuccauguuuugg......     1       1       seq
...........................................uaagAgcuuccauguuuugg......     2       1       seq
...........................................uaaguAcuuccauguuuugg......     4       1       seq
...........................................uaagugcuuccGuguuuugg......     5       1       seq
...........................................uaagugcuuccauguuGugg......     1       1       seq
...........................................uaagugcuuccaugCuuugg......     4       1       seq
...........................................uaagugcuCccauguuuugg......     13      1       seq
...........................................uaagugcuuccauguuuuCg......     7       1       seq
...........................................uaagugUuuccauguuuugg......     5       1       seq
...........................................uaagugcuuccauguuuugU......     3       1       seq
...........................................uaagugcuAccauguuuugg......     3       1       seq
...........................................uaagugcAuccauguuuugg......     1       1       seq
...........................................uaagugcuuccauguuuuUg......     81      1       seq
...........................................uaagugcuuccaCguuuugg......     6       1       seq
...........................................uaagugcuuUcauguuuugg......     5       1       seq
...........................................uaagugcuuccauguuCugg......     6       1       seq
...........................................Gaagugcuuccauguuuugg......     3       1       seq
...........................................uaagugcuuccaGguuuugg......     2       1       seq
...........................................uaagugcuuccauguuuGgg......     6       1       seq
...........................................uaagugcuuccauguuuCgg......     15      1       seq
...........................................uaagugcuuccauguCuugg......     8       1       seq
...........................................uaagugcGuccauguuuugg......     1       1       seq
...........................................uaagCgcuuccauguuuugg......     4       1       seq
...........................................uaagugcuuccauguuuugC......     6       1       seq
...........................................uaagugcuuccauguAuugg......     3       1       seq
...........................................uaagugcuucAauguuuugg......     3       1       seq
...........................................uGagugcuuccauguuuugg......     14      1       seq
...........................................Gaagugcuuccauguuuuggu.....     1       1       seq
...........................................uaagugcuuccaGguuuuggu.....     2       1       seq
...........................................uaagugcuCccauguuuuggu.....     11      1       seq
...........................................uaagAgcuuccauguuuuggu.....     1       1       seq
...........................................uaagCgcuuccauguuuuggu.....     11      1       seq
...........................................uaagugcuucUauguuuuggu.....     6       1       seq
...........................................uaagugcAuccauguuuuggu.....     1       1       seq
...........................................uaagugcuuccauguuuuggG.....     103     1       seq
...........................................uaagugcuuAcauguuuuggu.....     1       1       seq
...........................................uaagugcuAccauguuuuggu.....     7       1       seq
...........................................Aaagugcuuccauguuuuggu.....     5       1       seq
...........................................uaagugcGuccauguuuuggu.....     1       1       seq
...........................................uaUgugcuuccauguuuuggu.....     4       1       seq
...........................................uaagugcuuccauguuuuUgu.....     1       1       seq
...........................................uaagugcuuccauguuCuggu.....     11      1       seq
...........................................uaagugcuuccaugGuuuggu.....     4       1       seq
...........................................uaagugUuuccauguuuuggu.....     3       1       seq
...........................................uaagugcuuccauguuuAggu.....     3       1       seq
...........................................uaagugcuuccaugCuuuggu.....     18      1       seq
...........................................uaagugcuuccauguGuuggu.....     3       1       seq
...........................................uaagugcuuccauguuuugCu.....     1       1       seq
...........................................uaaguAcuuccauguuuuggu.....     1       1       seq
...........................................uaagugcuGccauguuuuggu.....     4       1       seq
...........................................uaagugcuuccauguuuuggC.....     173     1       seq
...........................................uaagugcuuUcauguuuuggu.....     8       1       seq
...........................................uaagugcuuccauguuuuCgu.....     1       1       seq
...........................................uUagugcuuccauguuuuggu.....     2       1       seq
```

```
     hsa-miR-302a-5p
                                           hsa-miR-302a-3p
ccaccacuuaaacguggauguacuugcuuugaaacuaaagaaguaagugcuuccauguuuuggugaugg

...........................................uaagugcuuccauguuAuggu.....    1           1           seq
...........................................uaGgugcuuccauguuuuggu.....    21          1           seq
...........................................uaagugcuuccauguuuugAu.....    3           1           seq
...........................................uGagugcuuccauguuuuggu.....    19          1           seq
...........................................uaagugcuuccaCguuuuggu.....    8           1           seq
...........................................uaagugcuuccGuguuuuggu.....    10          1           seq
...........................................uaagugcuuccauguuuCggu.....    11          1           seq
...........................................uaagugcuuccauguuuugUu.....    2           1           seq
...........................................Caagugcuuccauguuuuggu.....    13          1           seq
...........................................uaagugcCuccauguuuuggu.....    18          1           seq
...........................................uaagugcuuccauguuuGggu.....    8           1           seq
...........................................uaagugcuuccauguuuuggu.....    4723        0           seq
...........................................uaagugcuuccauguuGuggu.....    1           1           seq
...........................................uaagugcuuccCuguuuuggu.....    1           1           seq
...........................................uaagugcuuccauguAuuggu.....    1           1           seq
...........................................uaagugcuuccauAuuuuggu.....    3           1           seq
...........................................uaagugcuuccauguCuuggu.....    9           1           seq
...........................................uaagugcuuccauguuuuggA.....    11          1           seq
...........................................uaaAugcuuccauguuuuggu.....    6           1           seq
...........................................Gaagugcuuccauguuuuggug....    24          1           seq
...........................................uaagugcuuccauguuuugCug....    3           1           seq
...........................................uaagugcuuccaugGuuuggug....    24          1           seq
...........................................uaUgugcuuccauguuuuggug....    15          1           seq
...........................................uaagugcuuccauguCuuggug....    66          1           seq
...........................................uaagugcuuccauguuuugAug....    24          1           seq
...........................................uaagugcuucAauguuuuggug....    8           1           seq
...........................................Aaagugcuuccauguuuuggug....    21          1           seq
...........................................uaagugcuuccGuguuuuggug....    71          1           seq
...........................................uUagugcuuccauguuuuggug....    21          1           seq
...........................................uaagugcuuccauguuuugUug....    2           1           seq
...........................................uaagugcuuccauguuCuggug....    36          1           seq
...........................................uaagugcuuccauCuuuuggug....    1           1           seq
...........................................uaaguUcuuccauguuuuggug....    3           1           seq
...........................................uaagugUuuccauguuuuggug....    26          1           seq
...........................................uCagugcuuccauguuuuggug....    4           1           seq
...........................................uaagugcuuccauguuuuggCg....    141         1           seq
...........................................uaagugcuucUauguuuuggug....    31          1           seq
...........................................uaagugcGuccauguuuuggug....    3           1           seq
...........................................uaagugcuuUcauguuuuggug....    55          1           seq
...........................................uaagugcuuAcauguuuuggug....    3           1           seq
...........................................uaagugcuuccaGguuuuggug....    13          1           seq
...........................................uaagugcuuccauguuuugguC....    12          1           seq
...........................................uaagugcuCccauguuuuggug....    107         1           seq
...........................................uaGgugcuuccauguuuuggug....    89          1           seq
...........................................uaagugcuuccaugCuuuggug....    52          1           seq
...........................................uaagugcuuccauguuuGggug....    90          1           seq
...........................................uaagugcuuccaugAuuuggug....    6           1           seq
...........................................uaagugcuuccaAguuuuggug....    6           1           seq
...........................................uaagugAuuccauguuuuggug....    1           1           seq
...........................................uaagugcuuccauguuuuggug....    28838       0           seq
...........................................uaagugcuuccauguGuuggug....    4           1           seq
...........................................uaagugcuuccauguAuuggug....    3           1           seq
...........................................uaagugcuuccauguuuuAgug....    844         1           seq
...........................................uaagugcuuccauguuuCggug....    45          1           seq
...........................................uaagugcuuccaCguuuuggug....    78          1           seq
...........................................uaagugcuuccauguuuugguU....    11          1           seq
...........................................uaagugcAuccauguuuuggug....    9           1           seq
...........................................uaaguAcuuccauguuuuggug....    52          1           seq
...........................................uaagugcuuccUuguuuuggug....    7           1           seq
...........................................uaagugcCuccauguuuuggug....    94          1           seq
...........................................uaagugcuAccauguuuuggug....    21          1           seq
...........................................uaagugcuuccauAuuuuggug....    15          1           seq
...........................................uGagugcuuccauguuuuggug....    97          1           seq
...........................................uaagugcuuccauguuuAggug....    10          1           seq
...........................................uaagugcuuccauUuuuuggug....    2           1           seq
...........................................Caagugcuuccauguuuuggug....    45          1           seq
...........................................uaagugcuuccauguuuuUgug....    14          1           seq
...........................................uaCgugcuuccauguuuuggug....    3           1           seq
...........................................uaagGgcuuccauguuuuggug....    1           1           seq
```

```
     hsa-miR-302a-5p
                                           hsa-miR-302a-3p
ccaccacuuaaacguggauguacuugcuuugaaacuaaagaaguaagugcuuccauguuuuggugaugg

...........................................uaagugcuuGcauguuuuggug....    1     1     seq
...........................................uaagugcuucGauguuuuggug....    2     1     seq
...........................................uaagCgcuuccauguuuuggug....    77    1     seq
...........................................uaagugcuuccauguuuugguA....    578   1     seq
...........................................uaagAgcuuccauguuuuggug....    8     1     seq
...........................................uaagugcuuccauguuuuggAg....    27    1     seq
...........................................uaaguCcuuccauguuuuggug....    6     1     seq
...........................................uaagugcuuccauguuGuggug....    1     1     seq
...........................................uaagugcuGccauguuuuggug....    16    1     seq
...........................................uaagugcuuccauguuAuggug....    5     1     seq
...........................................uaaAugcuuccauguuuuggug....    17    1     seq
...........................................uaagugcuuccauguuuuggGg....    338   1     seq
...........................................uUagugcuuccauguuuuggugа...    1     1     seq
...........................................uaaguAcuuccauguuuuggugа...    2     1     seq
...........................................uaagugcuuccauguuCuggugа...    4     1     seq
...........................................uaagCgcuuccauguuuuggugа...    1     1     seq
...........................................uaagugcuuccauguuuuggCga...    2     1     seq
...........................................uaagugcuuccGuguuuuggugа...    1     1     seq
...........................................uaagugcuuccauguuuuggugа...    972   0     seq
...........................................uaagugcuuccauguuuuggugC...    5     1     seq
...........................................uGagugcuuccauguuuuggugа...    2     1     seq
...........................................uaagugcuucUauguuuuggugа...    1     1     seq
...........................................uaagugcuuccauguuuCgguga...    4     1     seq
...........................................uaagugcuuccauAuuuuggugа...    1     1     seq
...........................................uaGgugcuuccauguuuuggugа...    3     1     seq
...........................................uaagugcuuccauguuuugguAa...    4     1     seq
...........................................uaagugcCuccauguuuuggugа...    1     1     seq
...........................................uaagugcuuccaGguuuuggugа...    1     1     seq
...........................................uaagugcuuccauguuuuggGga...    2     1     seq
...........................................uaagugcuuccauguuuuAguga...    1     1     seq
...........................................uaagugcuuccauguuuuggugU...    10    1     seq
...........................................uaagugcuuccauguuuuggAga...    1     1     seq
...........................................uaagugcuuccaugCuuuggugа...    5     1     seq
...........................................uaagugcuuccauguuuugAuga...    2     1     seq
...........................................uaagugcuuccaCguuuuggugа...    1     1     seq
...........................................uaagugcuCccauguuuuggugа...    8     1     seq
...........................................uaagugUuuccauguuuuggugа...    2     1     seq
...........................................uaagugcuuUcauguuuuggugа...    2     1     seq
...........................................uaUgugcuuccauguuuuggugа...    1     1     seq
...........................................uaagugcuuccauguuuuggugG...    9     1     seq
...........................................uaagugcuuccauguCuuggugа...    3     1     seq
...........................................uaagAgcuuccauguuuuggugа...    1     1     seq
...........................................uaagugcuuccauguuAuggugа...    2     1     seq
...........................................uaaAugcuuccauguuuuggugа...    1     1     seq
...........................................uaagugcuuccauguuuuggugaG..    1     1     seq
...........................................uaagugcuuccauguuuuAgugau..    1     1     seq
...........................................uaagugcuuccauguuuuggugaA..    140   1     seq
...........................................uaagugcuuccauguuuuggugau..    4     0     seq
...........................................uaagugcuuccauguuuuggugUu..    1     1     seq
...........................................uaagugcuuccauguuuuggugaC..    4     1     seq
............................................aagugcuuccauguuuAg.......    3     1     seq
............................................aagugcuuccauguuuug.......    8     0     seq
............................................Uagugcuuccauguuuugg......    4     1     seq
............................................aagugcuuccauguuuuUg......    1     1     seq
............................................aagugcuuccauguuuugg......    20    0     seq
............................................aagugcuuccauguuuuggG.....    3     1     seq
............................................Cagugcuuccauguuuuggu.....    1     1     seq
............................................aagugAuuccauguuuuggu.....    1     1     seq
............................................aagugcuuccauguuCuggu.....    1     1     seq
............................................aagugcuuccauguuuuggu.....    60    0     seq
............................................aGgugcuuccauguuuuggu.....    1     1     seq
............................................aagugcuuccauguuuuggC.....    3     1     seq
............................................aagugcuuccauguAuuggug....    1     1     seq
............................................aagugcuAccauguuuuggug....    2     1     seq
............................................Cagugcuuccauguuuuggug....    1     1     seq
............................................aagugcuuccauguuuugguC....    1     1     seq
............................................aagugcuuccauguuuAggug....    1     1     seq
............................................Uagugcuuccauguuuuggug....    6     1     seq
............................................aagugcuuccauguuuuggCg....    8     1     seq
```

```
     hsa-miR-302a-5p
                                            hsa-miR-302a-3p
ccaccacuuaaacguggauguacuugcuuugaaacuaaagaaguaagugcuuccauguuuugguguaugg

............................................aagugcuCccauguuuuggug....    4       1       seq
............................................aagugcuuccauguuuugCug....    1       1       seq
............................................aagugcuuUcauguuuuggug....    2       1       seq
............................................aagugcuuccauguuuGggug....    6       1       seq
............................................aagugcuuccauguuuuggug....    1259    0       seq
............................................aagugcuuccauguuuuggGg....    17      1       seq
............................................aagugcuuGcauguuuuggug....    1       1       seq
............................................Gagugcuuccauguuuuggug....    6       1       seq
............................................aagugcuuAcauguuuuggug....    2       1       seq
............................................aagCgcuuccauguuuuggug....    2       1       seq
............................................aagugcuuccaCguuuuggug....    3       1       seq
............................................aagugcuuccauguuuuAgug....    17      1       seq
............................................aagugcuuccauguuGuggug....    1       1       seq
............................................aagugAuuccauguuuuggug....    1       1       seq
............................................aagugUuuccauguuuuggug....    1       1       seq
............................................aUgugcuuccauguuuuggug....    1       1       seq
............................................aagugcuuccauguuuugguA....    6       1       seq
............................................aagugcuuccauguuCuggug....    4       1       seq
............................................aagugcuuccauguGuuggug....    1       1       seq
............................................aagugcuuccauguuuugAug....    1       1       seq
............................................aGgugcuuccauguuuuggug....    6       1       seq
............................................aagugcuuccaugCuuuggug....    1       1       seq
............................................aagugcuuccauguCuuggug....    4       1       seq
............................................aagugcCuccauguuuuggug....    2       1       seq
............................................aaAugcuuccauguuuuggug....    1       1       seq
............................................aagugcuuccGuguuuuggug....    3       1       seq
............................................aagugcuuccauguuuCggug....    9       1       seq
............................................aagugcuuccauUuuuuggug....    1       1       seq
............................................aagugcuuccauguuuuggugU...    1       1       seq
............................................aagugcuuccauguuuuggugG...    3       1       seq
............................................aagugcuuccauguuuuggugua...    59      0       seq
............................................aagugcuCccauguuuuggugua...    1       1       seq
............................................Gagugcuuccauguuuuggugua...    1       1       seq
............................................aagugcuuccauguuuugguguaA..    10      1       seq
.............................................agugcuuccauguuuuggu.....    1       0       seq
.............................................agugcuuccauguuuuggug....    12      0       seq
.............................................agugcuuccauguuuuAgug....    0       1       seq
.............................................aguAcuuccauguuuuggug....    1       1       seq
.............................................agugcuuccauguuuGggug....    1       1       seq
.............................................agugcuuccauguuuuggCg....    1       1       seq
.............................................agugcuuccauguuuuAguga...    1       1       seq
..............................................gugcuuccauguuuuggu.....    1       0       seq
..............................................gugcuuccauguuuuggGg....    1       1       seq
..............................................gugcuuccauguuuuggugua...    1       0       seq
...............................................ugcuuccauguuuuggug....    4       0       seq
...............................................ugcuuccauguuuugguAa...    1       1       seq
................................................gcuuccauguuuuggugua...    1       0       seq
```
